# Small-molecule probe for IBD risk variant GPR65 I231L alters cytokine signaling networks through positive allosteric modulation

**DOI:** 10.1126/sciadv.adn2339

**Published:** 2024-07-19

**Authors:** Ilona Neale, Clark Reddy, Zher Yin Tan, Bihua Li, Partha P. Nag, Joshua Park, Jihye Park, Kimberly L. Carey, Daniel B. Graham, Ramnik J. Xavier

**Affiliations:** ^1^ Broad Institute of MIT and Harvard, Cambridge, MA 02142, USA.; ^2^ Department of Chemistry and Chemical Biology, Harvard University, Cambridge, MA 02138, USA.; ^3^ Center for Computational and Integrative Biology, Massachusetts General Hospital, Harvard Medical School, Boston, MA 02114, USA.; ^4^ Department of Molecular Biology, Massachusetts General Hospital, Harvard Medical School, Boston, MA 02114, USA.

## Abstract

The proton-sensing heterotrimeric guanine nucleotide–binding protein–coupled receptor GPR65 is expressed in immune cells and regulates tissue homeostasis in response to decreased extracellular pH, which occurs in the context of inflammation and tumorigenesis. Genome-wide association studies linked *GPR65* to several autoimmune and inflammatory diseases such as multiple sclerosis and inflammatory bowel disease (IBD). The loss-of-function GPR65 I231L IBD risk variant alters cellular metabolism, impairs protective tissue functions, and increases proinflammatory cytokine production. Hypothesizing that a small molecule designed to potentiate GPR65 at subphysiological pH could decrease inflammatory responses, we found positive allosteric modulators of GPR65 that engage and activate both human and mouse orthologs of the receptor. We observed that the chemical probe BRD5075 alters cytokine and chemokine programs in dendritic cells, establishing that immune signaling can be modulated by targeting GPR65. Our investigation offers improved chemical probes to further interrogate the biology of human GPR65 and its clinically relevant genetic variants.

## Teaser

Structure-activity relationship study identifies small molecules that induce signaling in autoimmunity risk variant GPR65 I231L.

## INTRODUCTION

Autoimmune diseases are pathologically heterogeneous chronic conditions characterized by aberrant immune regulation and loss of tissue homeostasis. These diseases are modulated by the convergence of several environmental and genetic factors, which obscures our understanding of disease onset and progression. In the past decade, advances in sequencing technology have enabled large-scale genome-wide association studies (GWAS) and fine-mapping that facilitate the identification of genetic determinants of disease susceptibility and the deconvolution of complex host immunomodulatory pathways. The accelerated discovery of disease-associated genes raises the challenge of matching coding variants to functions. While knock-out and overexpression models provide insights into overall gene function, they are limited in their ability to characterize variant phenotypes at higher resolution. Mechanistic details of disease-associated variants can be interrogated more rigorously with a chemistry-based approach. Furthermore, therapeutic hypotheses can be evaluated by designing molecules that mimic or reverse the effects of protective and risk variants in mouse models. Drug mechanisms rooted in genetic evidence, particularly for genes prioritized by GWAS and fine-mapping, have greater success progressing through clinical development (*1*, *2*). Leveraging available genetic data is therefore advantageous to inform the development of previously unexplored therapeutic hypotheses. Moreover, improvements in medicinal chemistry and synthesis techniques provide additional opportunities for modulating target gene functions and protein–protein interactions through precise small-molecule design.

GPR65 is a proton-activated heterotrimeric GTP-binding protein (G protein)–coupled receptor (GPCR) expressed primarily on myeloid and lymphoid immune cells (*3*–*7*) that has been implicated through genetics in multiple autoimmune and inflammatory diseases. GWAS have identified numerous variants in the *GPR65* locus that are in linkage disequilibrium with each other and associated with multiple sclerosis (*8*) and inflammatory bowel disease (IBD) (*9*–*11*). *GPR65* variants have also been linked to susceptibility and severity of ankylosing spondylitis (*12*) and atopic dermatitis (*13*). GPR65 has been shown to play a protective role in mouse models of acute lung injury, where GPR65 deficiency increases lung damage and neutrophil infiltration (*14*). GPR65 deficiency also exacerbates disease in collagen antibody-induced arthritis models (*15*), yet loss of GPR65-expressing CD4^+^ T cells protects against autoimmune encephalomyelitis in Rag1^−/−^ mice (*16*, *17*).

Expression of GPR65 has been shown to be critical for innate host defense (*18*–*20*), and loss of GPR65 exacerbates inflammatory pathology in *Citrobacter rodentium* and T cell transfer models of colitis (*18*, *19*). Although inhibition of pH-sensing family members GPR4 and GPR68 has been reported to improve outcomes in murine colitis models (*21*, *22*), only GPR65 has annotated genetic variants that correlate with IBD. Fine-mapping of the *GPR65* locus identified the missense coding mutation Ile231Leu (I231L) as an IBD risk variant with a reported allele frequency of 9.2% in individuals with European ancestry (*23*) and an enriched frequency of 20.6% in East Asian populations (*24*). Functional studies show that GPR65 I231L is a loss-of-function variant resulting in decreased receptor signaling (*18*, *19*, *25*). Investigations into GPR65 signaling have shown that GPR65 expressed on the plasma membrane can traffic to the endosome and signal constitutively (*26*, *27*). In addition to reduced signaling, cells expressing the variant I231L display aberrant lysosomal morphology and defective degradation associated with impaired clearance of intracellular bacterial pathogens (*18*, *25*). In addition, loss of GPR65 affects tissue-protective T cell responses through altered cellular metabolism and impaired cytokine production in T helper 17 (T_H_17) and T_H_22 cells (*17*, *19*, *28*). Rag1^−/−^ mice reconstituted with GPR65 I231L or GPR65 knockout (KO) CD4^+^ T cells demonstrate more severe colitis phenotypes compared to those reconstituted with GPR65 wild-type (WT) CD4^+^ T cells (*19*). Suppression of inflammatory cytokine production in macrophages has been shown to be mediated by GPR65 via the Gα_s_/cyclic adenosine monophosphate (cAMP)/cAMP-dependent protein kinase A signaling pathway (*29*). As a pH-sensing regulator of immune integrity, developing a small-molecule activator of GPR65 could restore tissue homeostasis with unique potential to target the acidified niches present during inflammation (*30*, *31*).

Here, we identified two new molecules, BRD5075 and BRD5080, as potent activators of GPR65. After screening a library of drug-like molecules for the ability to activate GPR65, we used a structure-activity relationship (SAR) approach to seek improved potency of the top hit, BRD2813, and enhance selectivity for both mouse and human receptor orthologs. The resulting molecules induced GPR65-specific signaling, measured by increased cAMP activity and G protein recruitment. Our interrogation of the signal transduction pathway revealed that these molecules are positive allosteric modulators (PAMs)—molecules that bind receptors and alter responses to orthosteric ligands by inducing conformational changes in the orthosteric site or the receptor itself (*32*)—and therefore active at decreased pH, where GPR65 is partially activated by proton concentration. In dendritic cells, we found that treatment with BRD5075 was sufficient to modulate the expression of proinflammatory cytokines and chemokines. Using key insights from genetics, our investigation identified small-molecule probes to establish a proof of concept that PAMs can potentiate GPR65 and the risk variant I231L to rebalance inflammatory responses.

## RESULTS

### BRD2813 activates human GPR65 through positive allosteric modulation

Studying the role of GPR65 and the I231L variant in tissue homeostasis in greater detail requires the ability to modulate receptor signaling, which can be achieved by targeting GPR65 with small molecules. The published agonist for GPR65 and current literature standard, BTB09089, induces increased cAMP production through the Gα_s_-coupled signal transduction pathway in murine macrophages and T cells (*33*) (fig. S1A), but its activity on human-derived cells remains poorly characterized. We developed a cell-based assay to measure cAMP production in preparation for a high-throughput screen for potential GPR65-activating molecules. Stable cell lines were created using CRISPR to knock out GPR65 in WT HeLa cells and then were reconstituted with WT human GPR65 (hGPR65), WT mouse GPR65 (mGPR65), or empty vector through lentiviral transduction. Forskolin, which activates adenylyl cyclase, was used as a positive control for increasing intracellular cAMP levels independent of GPR65 signaling. The assay was optimized at pH 7.1, around the 20% effective concentration of the hGPR65 pH response curve (fig. S1B), to detect hits that potentiate GPR65 below physiological pH but over background cAMP levels produced by proton concentration and to capture the activity of agonists as well as PAMs.

**Fig. 1.**
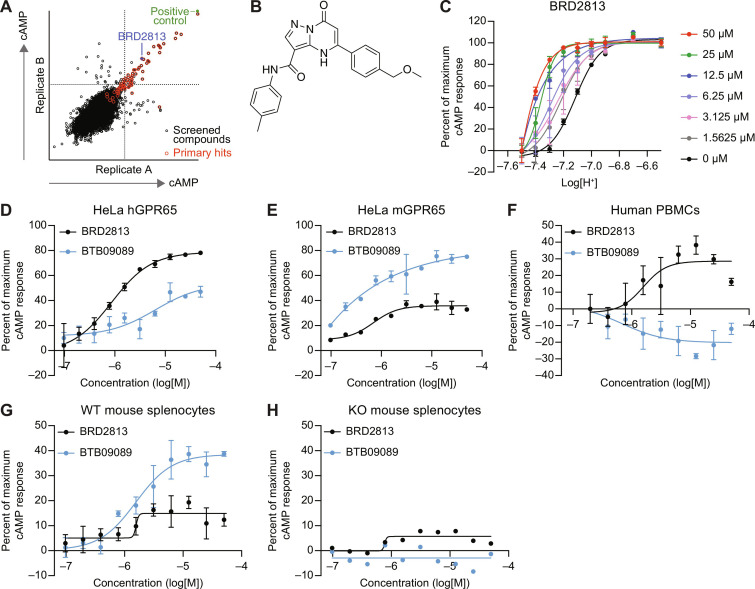
BTB09089 and BRD2813 induce species-selective GPR65-dependent cAMP production. (**A**) Replicate plot from a high-throughput screen of 21,000 compounds showing relative cAMP production per compound for each of two technical replicates after 30-min stimulation of GPR65 KO HeLa cells reconstituted with hGPR65 at pH 7.1. (**B**) Chemical structure of BRD2813. (**C**) pH-dependent cAMP production in the presence of 0 to 50 μM BRD2813, showing positive allosteric modulation behavior. pH curves were normalized to pH 6.6 for maximum response and 7.5 for minimum cAMP response (mean ± SD, *n* = 2). (**D** and **E**) cAMP response after stimulation with 0 to 50 μM BTB09089 or BRD2813 in GPR65 KO HeLa cells reconstituted with (D) hGPR65 and (E) mGPR65 at pH 7.2 (mean ± SD, *n* = 2). (**F**) cAMP production in human PBMCs after 30-min stimulation with 0 to 50 μM BTB09089 or BRD2813 at pH 7.2 (mean ± SD, *n* = 2). (**G** and **H**) GPR65-dependent cAMP induction after 30-min stimulation with 0 to 50 μM BTB09089 or BRD2813 in (G) GPR65 WT and (H) GPR65 KO mouse splenocytes at pH 7.2 (mean ± SD, *n* = 3). The percent of maximum cAMP response was calculated relative to treatment with 100 μM forskolin and dimethyl sulfoxide. Data represent at least two independent experiments.

The initial screen was conducted in hGPR65-expressing cells with a commercial collection of 21,000 drug-like small molecules selected to maximize chemical diversity. We identified 70 primary hits that induced cAMP production (Fig. 1A) and validated one compound, BRD2813 (Fig. 1B). This molecule was the only primary hit that showed dose-dependent activity in hGPR65-expressing cells without inducing cAMP production in control cells (fig. S1C and rows 85 to 88 in data S1). Treating hGPR65-expressing cells with increasing concentrations of BRD2813 potentiated GPR65 activity by orthosteric proton liganding, causing a leftward shift in the pH response curve of GPR65 that is consistent with positive allosteric modulation (Fig. 1C). GPR65 was maximally active at pH 6.8, and the compound showed the greatest effect between pH 7.1 and 7.3 (fig. S1D). BRD2813 produced a greater cAMP response in HeLa cells expressing hGPR65 than the current standard, BTB09089 (Fig. 1D). Conversely, BTB09089 was more effective in HeLa cells expressing the mouse ortholog (Fig. 1E). When assayed in primary cells, BRD2813 outperformed BTB09089 in human peripheral blood mononuclear cells (PBMCs) (Fig. 1F), but BTB09089 induced higher cAMP production than BRD2813 in a GPR65-dependent manner in splenocytes isolated from GPR65 WT and KO mice (Fig. 1, G and H). hGPR65 and mGPR65 have a sequence homology of 78%, which supports the notion that these receptors display differences in small-molecule binding (fig. S1E). The observed difference in species specificity of these molecules prompted the development of improved compounds capable of probing the function of GPR65 in both human- and murine-derived models.

### BRD2813 analogs activate hGPR65 and mGPR65

**Fig. 2.**
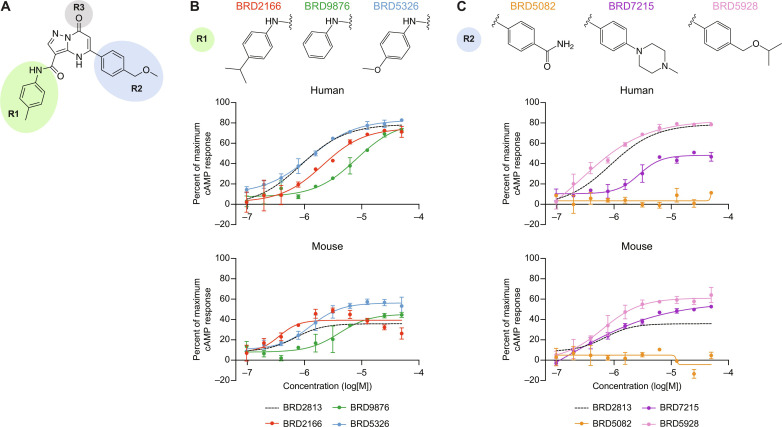
SAR study of BRD2813 yields several GPR65-specific analogs that robustly activate both hGPR65 and mGPR65. (**A**) Chemical structure of BRD2813 with R groups modified in the SAR study highlighted. (**B**) Examples of R1 modifications and resulting changes in cAMP production relative to BRD2813 in hGPR65- and mGPR65-expressing HeLa cells at pH 7.2 (mean ± SD, *n* = 2). (**C**) Examples of R2 modifications and resulting changes in cAMP production relative to BRD2813 in hGPR65- and mGPR65-expressing HeLa cells at pH 7.2 (mean ± SD, *n* = 2). The percent of maximum cAMP response was calculated relative to 100 μM forskolin and DMSO. Data represent at least two independent experiments.

BRD2813 served as the basis for a SAR study because of its GPR65-dependent induction of cAMP and its superior activity on hGPR65 compared to BTB09089. BRD2813 consists of an oxo-pyrazolopyrimidine core decorated with *N*-aryl carboxamide and aryl substituents at the C-3 and C-5 positions (R1 and R2 respectively in Fig. 2A) and a ketone at the C-7 position (R3 in Fig. 2A) We focused on improving compound activity toward mGPR65 while maintaining activity for hGPR65 by varying these three positions. The resulting 112 analogs (both commercially procured and chemically synthesized) were assessed for activity and species specificity using the cAMP assay (data S2). We generated a new suite of WT HeLa cell lines stably expressing hGPR65, variant hGPR65 (hGPR65 I231L), mGPR65, human GPR4 (hGPR4), human GPR68 (hGPR68), or green fluorescent protein (GFP) to test the analogs going forward and examine whether they promiscuously affected the activity of related pH-sensing receptors. After confirming that WT and GFP-expressing HeLa cells showed no pH-dependent change in cAMP activity (fig. S2A) and that hGPR65 and mGPR65 have comparable pH sensitivity (fig. S2B), the BRD2813 analogs were screened for activity in both hGPR65 and mGPR65 cell lines.

**Fig. 3.**
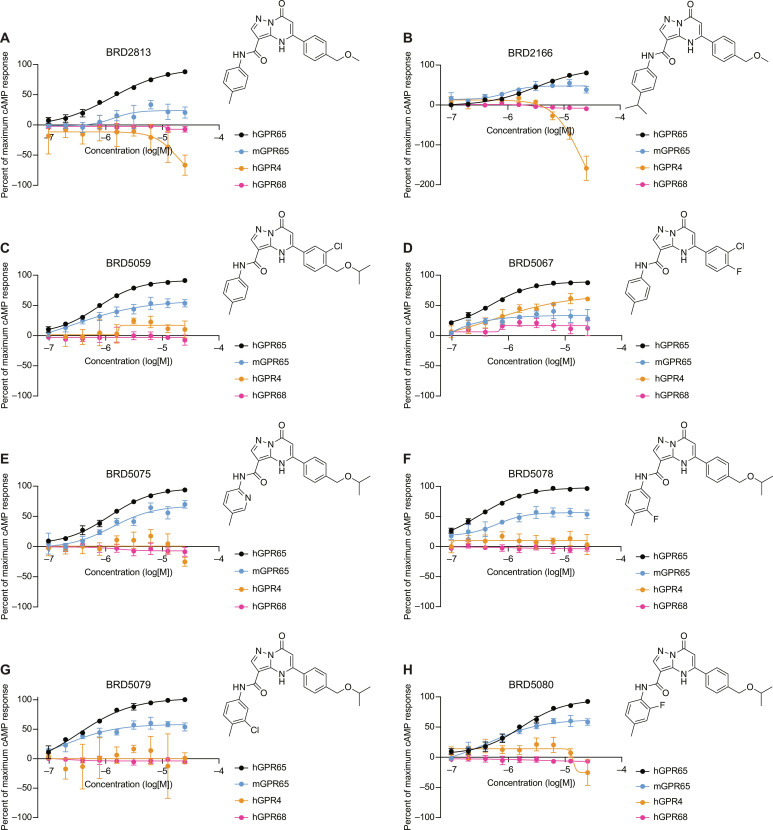
Specificity of prioritized molecules for GPR65. (**A** to **H**) cAMP production in HeLa cells expressing hGPR65, mGPR65, hGPR4, or hGPR68 at pH 7.2 (mean ± SD, *n* = 3). The percent of maximum cAMP response was normalized to pH 6.6 and DMSO. Data represent at least two independent experiments.

The cAMP assay revealed diverse activity profiles, with some compounds solely activating either hGPR65 or mGPR65 and a few that activated both receptors with high potency (data S2). Overall, the SAR campaign revealed the following observations. Changes to the ketone at C-7 (R3 in Fig. 2A) caused a substantial decrease or attenuation of activity; thus, further modifications to this group were not explored (data S2). Changes to the *para*-methyl group on *N*-aryl carboxamide substituents at C-3 yielded little improvement in hGPR65 activity compared to the parent molecule BRD2813 (Fig. 2B). Removal of the *para*-methyl group (BRD9876) was detrimental to potency, while swapping in an isopropyl (BRD2166) or a methoxy (BRD5326) group on the ring had little effect on cAMP production (Fig. 2B). The decreased potency of BRD9876 was also observed when assayed in mGPR65-expressing cells, while a slight improvement in mGPR65 activity was seen with BRD5326 compared to BRD2813 (Fig. 2B). Substitutions at the C-5 aryl ring had more varied consequences. The benzamide in BRD5082 was not tolerated by either receptor and eliminated all cAMP activity (Fig. 2C). Relative to BRD2813, the activity of BRD7215 produced contrasting effects on the GPR65 orthologs, decreasing cAMP production in hGPR65-expressing cells yet increasing activity in mGPR65-expressing cells (Fig. 2C). Lipophilic substitutions such as in BRD8716 and BRD7102 (rows 45 and 48; data S2) improved potency but had little effect on total cAMP production. One particularly strong analog, BRD5928, induced high hGPR65 activity while also substantially increasing cAMP production by mGPR65 (Fig. 2C). Last, several compounds were designed with the same *p*-alkylisopropoxy phenyl group at C-5 as in BRD5928 with variations at the ortho- and meta-positions of the *N*-aryl ring of the carboxamide at C-3. This approach yielded compounds BRD5059, BRD5075, BRD5078, BRD5079, and BRD5080, which along with BRD2166 and BRD5067, comprised a list of prioritized molecules that induced at least 40% cAMP activity with a submicromolar median effective concentration in both hGPR65 and mGPR65 (Fig. 3, A to H, data S2, and Supplementary Text).

We next interrogated the specificity of these compounds using the cell lines expressing hGPR4 and hGPR68, with pH 6.6 serving as a universal positive control for inducing cAMP production. BRD2813 and the prioritized analogs almost exclusively activated hGPR65 and mGPR65 with the exception of BRD5067, which additionally induced cAMP in the hGPR4 cell line (Fig. 3, A to H, and data S3). Among the prioritized molecules, BRD5059, BRD5075, BRD5078, BRD5079, and BRD5080 demonstrated the highest activity for hGPR65 and mGPR65 at 1.56 μM (fig. S3A). At the highest concentration tested, BRD2813 and BRD2166 caused a decrease in cAMP relative to dimethyl sulfoxide (DMSO) in the hGPR4 cells, suggesting an inhibitory effect that was not pursued further (Fig. 3, A and B).

We further tested the prioritized molecules in the hGPR65 I231L–expressing cell lines at pH 7.2 to confirm that these molecules activate the variant receptor. We found that the results from the hGPR65-expressing cells were recapitulated in the variant-expressing cells and again observed that these molecules outperform BTB09089 (fig. S2, C to E). A closer examination of the cAMP data showed that the hGPR65 I231L–expressing cells have a lower baseline of cAMP production at pH 7.2 than the hGPR65-expressing cells despite the cell lines having similar GPR65 expression (fig. S2, F to H), which is consistent with previous reports (*18*, *19*). In the predicted structure of GPR65, the I231L variant is located on transmembrane helix 6 toward the region where G protein is recruited, which may be affecting G protein interactions and signaling (fig. S2I).

Standard physical properties assessment of these prioritized compounds revealed that BRD5075 was the most soluble analog in phosphate buffer (116 μM), followed by BRD5080 (13.6 μM) (data S3). The rest of the prioritized analogs had suboptimal solubility falling below the measurable range of the assay. BRD5075 and BRD5080 had respective lipophilicity measurements (logD) of 3.57 and 3.62, while the logD of the other compounds again fell outside the measurable window of the assay (data S3). BRD5075 and BRD5080 also had the highest cAMP response against mGPR65 in HeLa cells, making these two compounds the most attractive molecular probe candidates to potentiate both hGPR65 and mGPR65 orthologs in further studies (Fig. 3, E and H, and data S3).

### Observed G protein recruitment of GPR65 PAMs

While BRD5075 and BRD5080 induced GPR65-dependent cAMP production, this step in the signal transduction pathway is downstream of the initial liganding event. To confirm that these compounds were interacting with the GPR65 receptor, we sought an approach that would demonstrate G protein recruitment. Mini G (mG) proteins are engineered cytosolic G proteins designed to imitate Gα_s_, Gα_i_, Gα_q_, and Gα_12_ proteins that can be used in conjunction with a split-luciferase reporter to measure GPCR activation, whereby ligand-receptor binding recruits cytosolic mG, and the interaction between mG and the GPCR produces luminescence (*34*). We used Expi293F cells coexpressing a GPCR fused to SmBiT and an mGs fused to LgBiT because our molecules induced cAMP production, which is consistent with the notion that they activate GPR65 through Gα_s_-coupled signaling. We used β-2 adrenergic receptor (ADRB2), a well-characterized Gα_s_-coupled receptor, to confirm that the assay measured canonical GPCR signaling pathways. Signaling in cells coexpressing ADRB2-SmBiT and LgBiT-mGs was activated by isoproterenol, a known ADRB2 agonist, but no change in luminescence was observed in response to hydrochloric acid (HCl), showing that decreasing the pH did not affect the integrity of the assay (fig. S3B).

**Fig. 4.**
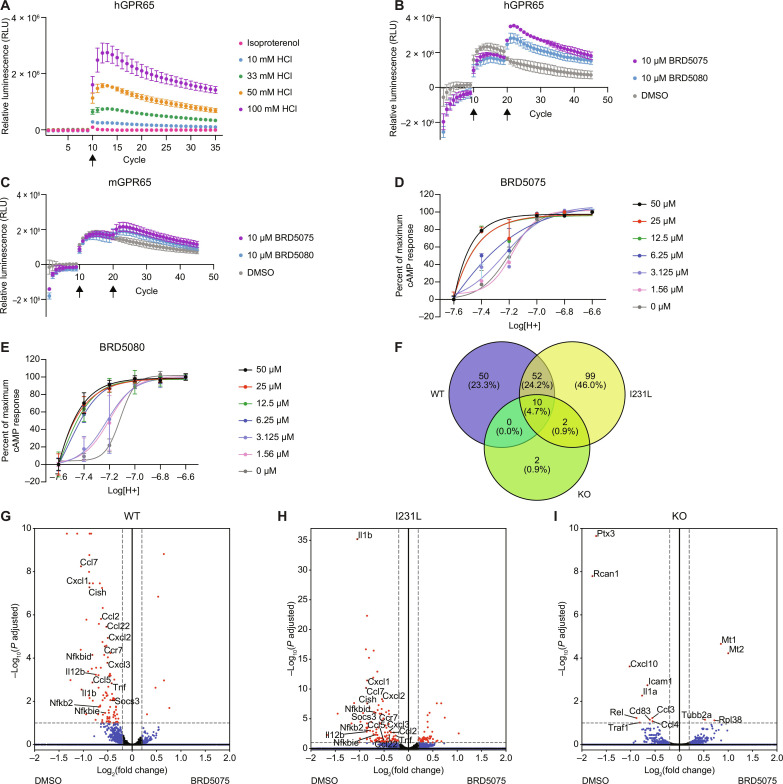
PAMs BRD5075 and BRD5080 demonstrate target engagement with hGPR65 and mGPR65. (**A**) pH-dependent increase in LgBiT-mGs recruitment to hGPR65-SmBiT in Expi293T cells in response to increasing concentrations of HCl added at cycle 10 (mean ± SD, *n* = 3). (**B** and **C**) Increased LgBiT-mGs recruitment with 10 μM BRD5075 or BRD5080 in Expi293T cells expressing (B) hGPR65-SmBiT and (C) mGPR65-SmBiT. pH was decreased at cycle 10 with 25 mM HCl, and compound was added at cycle 20. Data were normalized to cells treated with H_2_O at cycle 10 followed by DMSO at cycle 20 (mean ± SD, *n* = 3). (**D** and **E**) pH-dependent cAMP production in the presence of 0 to 50 μM (D) BRD5075 and (E) BRD5080, showing positive allosteric modulation of GPR65 in hGPR65-expressing HeLa cells. pH curves were normalized to pH 6.6 for maximum response and 7.6 for minimum (mean ± SD, *n* = 4). Data represent at least two independent experiments. (**F**) Venn diagram of significant DEGs in BMDCs isolated from WT, I231L, and KO mice treated with BRD5075 compared to DMSO at pH 7.2 (*n* = 3 technical replicates, one mouse per genotype). (**G** to **I**) Differential expression of genes in (G) WT, (H) I231L, and (I) KO BMDCs treated with BRD5075 compared to DMSO at pH 7.2. Red dots are significant DEGs defined by *P*_adj_ < 0.1 and log_2_(fold change) > |0.2|. Blue dots indicate DEGs that meet the threshold for fold change but not significance. Green dots indicate significant DEGs that do not meet the threshold for fold change. Black dots are DEGs that do not meet either threshold. 1 cycle = 90 s.

To confirm the assay was suitable to measure pH-sensitive GPCR responses, we stimulated cells coexpressing hGPR65-SmBiT and LgBiT-mGs with various concentrations of HCl. Decreasing the pH in this manner produced a robust dose response in luminescence, confirming that lowering pH causes mGs to be recruited to hGPR65 (Fig. 4A). Treating hGPR65-expressing cells with isoproterenol did not elicit a change in luminescence (Fig. 4A). We also observed that hGPR65 was able to recruit mGsi and mGsq and to a small extent mG12 proteins in response to low pH, indicating that GPR65 may also signal through the Gα_i_, Gα_q_, and Gα_12/13_ pathways (fig. S3, C to E), which has been suggested in other studies (*25*, *35*). We concluded that the observed change in luminescence from mGs recruitment to hGPR65 was due to pH-dependent activation of the receptor.

We further developed the assay to accommodate treatment with both PAMs and agonists by first spiking in either HCl or water to control whether GPR65 was already partially activated by pH, followed by the addition of compound. The original set of seven prioritized analogs, along with BRD2813 and BTB09089, were assayed for hGPR65 and mGPR65 potentiation. This assay reiterated the agonist behavior of BTB09089 toward mGPR65, with increases in luminescence independent of pH changes, and again demonstrated its limited activity toward hGPR65 (fig. S4, A and B). Of the prioritized analogs, only BRD5059, BRD5075, and BRD5080 showed engagement of both hGPR65 and mGPR65 (Fig. 4, B and C, and fig. S4, C to H). These three compounds showed activity when preceded by the addition of HCl but not water, providing evidence that these molecules are GPR65 PAMs (fig. S4, C to H), and again demonstrated specificity for GPR65 by failing to elicit responses in cells expressing hGPR4-SmBiT (fig. S4, I to K). Furthermore, BRD5075 and BRD5080 were able to recruit G protein in cells expressing the variant hGPR65 I231L (fig. S5, A and B). We also observed a dose-dependent effect of BRD5075 on mGs recruitment, indicating that signaling can be titrated by compound concentration (fig. S5C). Measuring cAMP production in hGPR65-expressing cells in response to different concentrations of BRD5075 and BRD5080 over a pH range of 6.6 to 7.6 further supported that these molecules are PAMs (Fig. 4, D and E), though the mechanism of allostery and whether they are efficacy or affinity driven remains to be explored. BRD5075 and BRD5080 were not cytotoxic to hGPR65-expressing cells over a 72-hour incubation period, with only a slight decrease in cell viability at 50 μM (fig. S5, D and E). Given that BRD5075 and BRD5080 exhibited mG protein recruitment for both human and mouse orthologs of GPR65 and had favorable solubility and logD values, we concluded that these were the most promising molecules for eliciting effects in primary cells and could be used at higher concentrations; however, molecules such as BRD5078 and BRD5079 and other analogs (data S2) that showed strong activity for hGPR65 may be useful tool compounds in future functional assays.

### BRD5075 regulates GPR65-dependent inflammatory cytokine and chemokine expression

We next sought to characterize the immunomodulatory consequences of treating primary immune cells with the GPR65-specific PAM BRD5075. GPR65-dependent cytokine regulation has previously been reported in antigen-presenting cells such as macrophages and bone marrow–derived dendritic cells (BMDCs), characterized by an increase in inflammatory cytokines in response to GPR65 deficiency (*19*, *29*, *33*). We hypothesized that potentiating GPR65 would decrease inflammatory cytokine production and performed RNA sequencing to study the effect of BRD5075 on BMDCs derived from GPR65 WT, I231L, and KO mice. We found that several cytokines and inflammatory regulators were down-regulated following treatment with BRD5075. After 2 hours of costimulation with zymosan to induce an inflammatory response and either 20 μM BRD5075 or DMSO at pH 7.2, the vast majority of significant differentially expressed genes (DEGs) were observed in WT or I231L but not KO cells treated with BRD5075, suggesting changes that are GPR65 dependent (Fig. 4F). Across all three genotypes, the cellular stress–related proteins *Mt1* and *Mt2* were up-regulated, which could be indicative of a GPR65-independent toxicity effect from impurities in the compound.

Among the genes with the highest increases in expression, *Fos* was up-regulated in WT BMDCs treated with BRD5075 (data S4). The Fos family of DNA binding proteins are CREB targets (*36*, *37*), supporting that the transcriptional effects of BRD5075 are mediated through the Gα_s_ signaling pathway in a GPR65-dependent manner. Cells treated with BRD5075 also displayed a suppression of inflammatory pathways compared to cells treated with DMSO (Fig. 4, G to I). In both WT and I231L BMDCs, genes for inflammatory cytokines (*Il12b*, *Il1b*, and *Tnf*) and chemokines (*Ccl7*, *Ccl2*, *Ccl22*, *Cxcl1*, *Cxcl2*, and *Cxcl3*) were down-regulated (Fig. 4, G and H). Complementary to decreased *Tnf* expression, two tumor necrosis factor (TNF) superfamily receptors, *Tnfrsf9* and *Tnfrsf26*, and the TNFα-induced apoptotic regulator, *Tnfaip8*, were down-regulated in WT BMDCs (data S4). Gene expression of several nuclear factor κB pathway regulators were also affected in both WT and I231L cells, including *Nfkbid*, *Nfkbie*, *Nfkbia*, and *Nfkb2*. Additional inflammatory signaling mediators, such as *Socs3* and *Cish*, were down-regulated as a result of BRD5075 treatment. Overall, genes associated with the interleukin-1 (IL-1), IL-12, and TNF pathways were suppressed upon addition of BRD5075 in a GPR65-dependent manner (Fig. 4, G to I).

Last, we investigated whether TNF secretion could be modulated by BRD5075 in human monocyte-derived dendritic cells (MDDCs). In MDDCs stimulated with lipopolysaccharide (LPS), we observed a strong inhibitory effect of low pH on TNF secretion and further TNF suppression upon addition of 5 μM and 20 μM BRD5075 (fig. S5F), though the effect at 5 μM did not reach statistical significance. Together, these results provide evidence that GPR65 can be targeted with a PAM to modulate cytokine and chemokine signaling networks and further establish GPR65 as a mediator of inflammatory signals with clinical relevance.

## DISCUSSION

GPCRs represent the largest family of receptors targeted for drug development (*38*–*40*). With more than 800 GPCRs in the human genome, these receptors have evolved to detect a wide array of ligands—from ions and photons to neurotransmitters, lipids, and hormones—and therefore function as key regulators of adaptive responses triggered by changes in the extracellular environment. The diversity of GPCR signaling pathways and the variety of ligands they recognize present challenges and opportunities in small-molecule pharmacology. Previous studies of the druggable GPCRome have used a combination of functional assays, computational approaches, and engineered yeast-based screens to identify ligands of GPCRs (*34*, *39*, *41*–*43*). In addition to displaying broad ligandability, GPCRs are highly druggable by diverse agonists, inverse agonists, and allosteric modulators that can have positive and negative effects on downstream signaling. These different modes of pharmacologic intervention offer flexibility in building an arsenal of small molecules to tune the activity of GPCRs. This is especially favorable in the context of genetics-guided targeting strategies, where the desired effect depends on whether a genetic variant impairs or augments receptor activity. The IBD risk variant GPR65 I231L impairs receptor function in macrophages, dendritic cells, and T cells, suggesting that an agonist or PAM could be used to tune receptor signaling toward the WT phenotype (*17*–*19*, *28*, *29*).

GPR65 plays an important role in innate immunity and regulating homeostasis in response to changes in the extracellular environment, particularly tissue acidification that occurs in inflammation and tumorigenesis (*30*, *31*). GPR65 confers tissue-protective properties in the context of IBD, and loss of function such as in the I231L variant leads to more severe disease phenotypes (*19*, *25*). Therefore, potentiating GPR65 activity with agonists or PAMs may reduce risk of developing colitis-associated colorectal cancer (*44*). On the other hand, the elevated levels of GPR65 expressed across several cancer types combined with the acidosis present in solid tumors may point to a role of this receptor in the tumor microenvironment (*45*, *46*). GPR65 has also been linked to intratumoral exhausted CD8^+^ T cells, which suggests that developing GPR65 inhibitors or negative allosteric modulators may be clinically relevant in cancer immunotherapy (*45*, *46*). The *GPR65* locus contains many variants other than I231L that have been associated across several autoimmune diseases including atopic dermatitis, multiple sclerosis, and IBD (*8*, *9*, *13*, *35*). Developing small molecules to target GPR65 provides an opportunity to understand how these variants function within different diseases in a way that is not possible through knockout models. An agonist may not affect receptor activity in exactly the opposite manner as knocking out the receptor nor act through the same mechanism as the endogenous ligand would on the WT receptor. Instead it is more informative to develop tool compounds such as PAMs to engage the receptor and monitor the resulting downstream effects.

In this study, we found chemical probes that activate GPR65 through positive allosteric modulation by way of the Gα_s_ signal transduction pathway. In surveying the chemical space of BRD2813, we found that subtle modifications to the structure were sufficient to cause changes in potency and selectivity. Allosteric binding sites of GPCRs have been shown to be less conserved than the orthosteric site, and many published cases of allosteric ligands have species differences (*32*, *47*). BRD5075 and BRD5080 exhibited robust induction of cAMP activity and recruited G protein for the WT and variant hGPR65 as well as for the mouse ortholog. The current literature standard BTB09089 had a diminished effect on hGPR65 in both assays. Hence, our PAMs provide a substantial advantage over BTB09089 for a single molecule that can be used to probe the function of GPR65 in both human and murine models of disease. Such probes are also valuable for elucidating cell biology of GPR65 signaling. Membrane trafficking is important to consider in the context of GPCR activation, and recent studies of GPR65 biology have aimed to understand the dynamics of GPR65 signaling and internalization (*26*, *27*). The effect of the I231L variant on these aspects of GPR65 signaling remains to be elucidated, but developing tool compounds to perturb GPR65 signaling could be valuable in these investigations.

Using PAMs as therapeutic agents has been proven clinically effective; benzodiazepines and brexanolone, the first FDA-approved drug for treating postpartum depression, act as PAMs for the GABA_A_ receptor (*48*–*50*). While agonists constantly engage the receptor, PAMs enhance the activity of a receptor under conditions when the endogenous ligand is present, thus retaining control mechanisms enforced by endogenous physiology. In this way, we propose that potentiating GPR65 with a PAM could target immune populations in the acidified inflammatory niche to down-modulate multiple cytokines and chemokines, rather than completely blocking a single cytokine with a neutralizing antibody. We demonstrated that when used at a pH where GPR65 is active, a GPR65-specific chemical probe can reduce the inflammatory signals that are produced by BMDCs, notably TNF and IL-12/23, which represent two key cytokines that are implicated in IBD and targeted by biologic therapies (*51*–*53*). Combining insights from genetics with advancing chemistry methods presents an empirical approach to discerning the functions of variants in disease-associated loci and has the potential to shape genetics-based drug discovery.

### Significance

Here, we present a strategy for using insights from human genetics to direct a chemical probe design for testing therapeutic hypotheses. Several disease-associated *GPR65* variants have been identified across different populations, making this GPCR an attractive potential target for therapeutic intervention. Of the identified single-nucleotide polymorphisms (SNPs) associated with IBD, the rs3742704 (I231L) SNP is the only coding variant. We developed tool compounds that potentiate GPR65 function and can be used to explore the biology of loss-of-function variants, including the GPR65 I231L variant that confers risk of IBD. We show that GPR65 can be targeted with PAMs to increase receptor signaling and elicit genotype-dependent changes in gene expression of inflammatory cytokine signaling networks.

## MATERIALS AND METHODS

### Experimental model and subject details

#### Mice

All mouse experiments complied with relevant ethical regulations and were performed according to protocol number 2003 N000158 approved by the Institutional Animal Care and Use Committee at the Massachusetts General Hospital. All mice were maintained in specific pathogen–free conditions and cohoused in the same room at the Massachusetts General Hospital’s animal facility maintained on a 12-hour light:dark cycle with a room temperature (RT) of 21°C and relative humidity of 30 to 70%. Mouse experiments were conducted with age-matched 6- to 8-week-old male C57BL/6 mice that were homozygous for GPR65 WT, GPR65 I231L, or GPR65 KO. The number of mice used in each experiment is provided in the figure legends.

#### Murine BMDCs

BMDCs were differentiated from bone marrow from in Dulbecco’s modified Eagle’s medium (DMEM) [10% heat-inactivated fetal bovine serum (FBS), 1X GlutaMAX, 1% penicillin-streptomycin] containing recombinant murine granulocyte-macrophage colony-stimulating factor (rmGM-CSF) (20 ng/ml; Peprotech, 315-03) for 7 days, supplemented with additional DMEM + rmGM-CSF on day 3. Cells were then scraped and seeded at 1 × 10^5^ cells per well in a 96-well microplate and allowed to adhere overnight.

#### Human PBMCs and monocytes

Human blood samples were collected from healthy donors at the Blood Donor Center at Massachusetts General Hospital in compliance with all relevant ethical regulations and according to protocol 2018P001504 approved by the Mass General Brigham Institutional Review Board. Donors provided informed written consent. Human PBMCs were isolated by density gradient separation using Ficoll-Paque PLUS (VWR- GE Life Sciences, catalog no. 17144002), as previously described (*54*). Isolated PBMCs were incubated overnight in DMEM (10% FBS, 0.2% sodium bicarbonate, 1X Glutamax) at 37°C, 5% CO_2_. Human monocytes were isolated from an unpurified Buffy Coat using RosetteSep human monocyte enrichment cocktail (StemCell Technologies, 15068) and frozen same day in FBS with 10% DMSO.

### Method details

#### cAMP detection in 21K primary screen

The Lance *Ultra* cAMP Kit (PerkinElmer, TRF0264) was used for cAMP detection, and included the following reagents: *ULight* anti-cAMP antibody, Eu-cAMP tracer, 7.5% BSA Stabilizer solution, and cAMP detection buffer. Stimulation buffer was prepared as described by the kit manufacturer as follows: Hanks buffered saline solution (HBSS, Life Technologies, 14025-126) containing 0.5 mM IBMX (3-isobutyl-1-methylxanthine, Sigma-Aldrich, I7018) and buffered with 1 M Hepes (15630-080). Stimulation buffer was then adjusted to pH 7.1 using 12 N HCl. HeLa cells were lifted using Versene and then suspended at 2.5 × 10^5^ cells/ml in stimulation buffer. Cells were then plated at 20 μl per well (5000 cells per well) in white, opaque OptiPlate 384-well MicroPlates (PerkinElmer, 6007299) using a Thermo Fisher Scientific Combi liquid dispenser. Compounds, stored at 5 mM in DMSO for the primary screen, or in a range of concentrations from 39 μM to 20 mM for dose curves, were transferred by the CyBio CyBi-Well Vario pin transfer tool with 100-nl pins (V&P FP1NS50H, 50-nl slot, 0.457-mm diameter, hydrophobic coated), into each assay well, 100 nl per well for a final assay concentration of 25 μM for the primary screen, or 195 nM to 100 μM for dose curves. Assay plates were then incubated at RT for 30 min. Eu-cAMP tracer was diluted 1:33 into detection buffer and the final solution then distributed into assay wells, 5 μl per well. *ULight* anti-cAMP antibody was diluted 1:100 into detection buffer, then distributed into assay wells, also 5 μl per well. Assay plates were then incubated at RT in the dark for 1 hour. Eu-cAMP tracer and Förster resonance energy transfer (FRET) emissions were then read using a PerkinElmer EnVision Multimode plate reader (ENVISION21040010), exciting each well at 320 nm [UV2 (TRF) 320 filter] and reading emissions at 615 nm (Europium 615 filter) and 665 nm (APC665 filter). Data analysis was performed using Genedata Screener and GraphPad Prism.

#### HeLa CRISPR GPR65 KO cell lines

HeLa KO cell lines were generated using the px330 plasmid CRISPR system as previously described (*18*). Briefly, HeLa cells were transfected with a Cas9 vector containing a gene-specific single-guide RNA to target the GPR65 coding exon. Forty-eight to 72 hours after transfection, single clones were obtained through limiting dilution and then sequenced to validate the KO. Reconstituted cell lines were produced by transducing KO cells with empty lentivirus or lentivirus containing hGPR65 or mGPR65. After 48 hours, cells were treated with puromycin (3 μg/ml; InvivoGen, ant-pr-1) and resistant cells were used in experiments.

#### cAMP detection in mouse splenocytes

cAMP was measured in cells using the Lance *Ultra* cAMP Kit. Spleens were harvested from GPR65 WT and GPR65 KO mice, homogenized and passed through a 70-μm cell strainer into harvest buffer [PBS, 0.5% bovine serum albumin (BSA), and 2 mM EDTA]. Cells were then centrifuged, washed with harvest buffer, and resuspended in red blood cell lysis buffer for 10 min at RT. Cells were counted and resuspended in assay buffer (25-ml DMEM, 0.5-ml 7.5% sodium bicarbonate, and 333-μl 7.5% BSA stabilizer) and adjusted to pH 7.2 with 1 M NaOH. Splenocytes were added to a white opaque OptiPlate 384-well microplates at a density of 100,000 cells per well in 20 μl. Compounds were added into the wells using a pin transfer tool, and plates were incubated at 37°C, 5% CO_2_ for 40 min. Each detection reagent (5 μl) was added to the plate and incubated at RT in the dark for 1 hour. Eu-cAMP tracer and FRET emissions were read using a PerkinElmer EnVision Multimode plate reader (ENVISION21040010). Data analysis was performed using GraphPad Prism.

#### cAMP detection in human PBMCs

cAMP was measured in cells using the Lance *Ultra* cAMP Kit. Compound dose curves were prepared in an assay buffer, which was made according to the manufacturer’s guidelines using DMEM instead of HBSS. Assay buffer was adjusted to desired pH using 1 M HCl or NaOH. PBMCs were collected, centrifuged, and resuspended in assay buffer. Dose curves and cells were added to a white opaque OptiPlate (PerkinElmer, 6007290) at a density of 20,000 cells per well. After incubation for 30 min, detection reagents were prepared according to the manufacturer’s guidelines and added to each well before incubating for 1 hour in the dark at RT. Eu-cAMP tracer and FRET emissions were read using a PerkinElmer EnVision Multimode plate reader (ENVISION21040010). Data analysis was performed using GraphPad Prism.

#### HeLa expression cell lines

HeLa stable expression cell lines were produced by lentiviral transduction. Briefly, lentiviral plasmids for each GPCR with a C-terminal V5 Tag (GKPIPNPLLGLDST) were made in a pLX317 backbone using competent *Escherichia coli* (New England Biolabs, C3050H). Lenti-X 293 T (Takara, 632810) cells were transfected with the lentiviral plasmids to produce virus. Low-passage HeLa cells (American Type Culture Collection, CCL-2) were then transduced with lentivirus. After 48 hours, HeLa cells were cultured under selection with puromycin (3 μg/ml) and allowed to recover and expand before storing stocks in liquid nitrogen.

#### cAMP detection assay in HeLa expression cell lines

cAMP was measured in cells using the Lance *Ultra* cAMP Kit. HeLa cells were cultured under puromycin (3 μg/ml) selection. One day before the experiment, cells were seeded in T75 flasks and allowed to adhere overnight. On the day of the experiment, cells were washed twice with Dulbecco’s PBS and lifted using prewarmed 1× versene solution (Gibco, 15040066). Cells were counted in HBSS (Gibco, 14025092) and then resuspended in stimulation buffer prepared according to the manufacturer’s instructions and adjusted to pH 7.2 using 1 M sodium hydroxide. Cells were added to the plate at a density of 1500 cells per well in white opaque 384-well assay ready microplates containing 100 nl of 10 mM compound in DMSO. Cells were stimulated for 30 min at RT, then lysed and incubated with detection reagents for 1 hour in the dark at RT. Plates were read on BMG Labtech PHERAstar FSX (excitation, 337 nm; emission, 620 and 665 nm). Data analysis was performed using GraphPad Prism.

#### Protein quantification

Cell lysates were treated with PNGaseF (New England Biolabs, P0704S) and run on a 4 to 20% polyacrylamide gel (Bio-Rad, 4561096) with a protein standard (Bio-Rad, 1610375) at 130 V for 35 min. The gel was transferred to a nitrocellulose membrane using an Invitrogen iBlot 2 Gel Transfer Device. Membranes were blocked for 1 hour (Li-Cor, 927-70001) and incubated with primary antibodies overnight at 4°C (Vinculin: Cell Signaling Technology, E1E9V, 1/1000; V5: Cell Signaling Technology, D3H8Q, 1/5000). Membranes were washed with TBS-T and then incubated for 1 hour with a secondary antibody in the dark at RT (Li-Cor, 926-32211, 1/10,000). Membranes were washed again in TBS-T and then imaged on Li-Cor Odyssey CLx and analyzed using the Image Studio software from Li-Cor. Data analysis was performed using GraphPad Prism.

#### Nano-BiT target engagement assay

Target engagement was measured in Expi293F (Gibco, 100044202) cells using the Nano-Glo Live Cell Assay System (Promega, N2011). Nano-BiT plasmids were cloned from GPR65-, GPR4-, and GPR68-Tango plasmids. GPR65-Tango (Addgene plasmid # 66370; http://n2t.net/addgene:66370; RRID:Addgene_66370), GPR4-Tango (Addgene plasmid # 66358; http://n2t.net/addgene:66358; RRID:Addgene_66358), and GPR68-Tango (Addgene plasmid # 66371; http://n2t.net/addgene:66371; RRID:Addgene_66371) were gifts from Bryan Roth. Expi293F cells were transfected 1 day before kinetics with 500 ng of each plasmid using ExpiFectamine 293 Transfection Kit (Gibco, A14525). After 24 hours, cells were collected, washed, and resuspended in room temperature 0.1% PBS-BSA, adjusted to pH 7.4 using 1 M NaOH. Cells were transferred to white opaque 96-well microplates (100 μl per well, PerkinElmer, 6005290), followed by addition of the Nano-Glo Live Cell Reagent (25 μl per well). Baseline luminescence was measured for 10 cycles (1 cycle = 90 s) before adding compounds. All luminescence measurements were made on BMG Labtech PHERAstar FSX. Data analysis was performed using GraphPad Prism.

#### Preparation of pH-stable DMEM for BMDC and MDDC stimulation

Different media were prepared on the basis of target pH. pH 6.6: DMEM [10% FBS, 1% penicillin/streptomycin (p/s), 20 mM BES (MilliporeSigma, B4554), 1X Glutamax (Gibco, 35050061), 3.75 mM sodium bicarbonate, and 41.25 mM NaCl]. pH 7.2: DMEM (10% FBS, 1% p/s, 20 mM BES, 1X Glutamax, 7.5 mM sodium bicarbonate, and 36.5 mM NaCl). pH 7.6: DMEM 10% FBS, 1% p/s, 20 mM BES, 1X Glutamax, and 44 mM sodium bicarbonate. Media was allowed to equilibrate overnight at 37°C, 5% CO_2_ in a vented conical tube, and then adjusted to desired pH immediately before the stimulation using 1 M NaOH.

#### Cytotoxicity assay

WT HeLa cells expressing hGPR65 were seeded at 2000 cells per well in 384-well plates. Compounds were tested in 10-point dose, starting at 50 μM. Cells were incubated with compounds for 72 hours at 37°C, 5% CO_2_. Cytotoxicity was measured using CellTiter-Glo Luminescent Cell Viability Assay (Promega, G7572) according to the manufacturer’s protocol. Luminescence measurements were made on BMG Labtech PHERAstar FSX. Data analysis was performed using GraphPad Prism.

#### RNA sequencing of BMDCs

BMDCs were stimulated with different compounds and pH conditions while simultaneously challenged with zymosan (5 μg/ml; Invivogen, tlrl-zyn) for 2 hours. The treatment conditions were as follows: (i) pH 7.2 + DMSO, (ii) pH 7.2 + 20 μM BRD5075, (iii) pH 7.6 + DMSO, and (iv) pH 7.6 + 5 μM PGE2 (Tocris, 2296). Cells were lysed with a lysis/binding buffer (Invitrogen, A33562).

Libraries were prepared using a modified SmartSeq2 protocol. RNA lysates were purified with Dynabeads mRNA Direct Purification kit (Life Technologies, 61011), followed by reverse transcription with Maxima Reverse Transcriptase (Thermo Fisher Scientific, EP0742) and whole transcription amplification (WTA) with KAPA HotStart HiFi 2x ReadyMix (Kapa Biosystems, KK2601). WTA products were purified with Ampure XP beads (Beckman Coulter, A63880). RNA sequencing libraries were constructed using Nextera XT DNA Library Preparation kit (Illumina, FC-131-1096). Samples were purified with Ampure XP, pooled in equal quantities, then run on 2% E-gel EX (Invitrogen, G402022). Extracted library from ~250 to 800 bps with gel extraction kit (Zymo Research, D4008). The libraries were sequenced on an Illumina NextSeq 500 instrument with NextSeq 500/550 High Output v2 kit (Illumina, 20024906) and the following parameters: paired End: Index1 8, Index2 8, Read1 38, Read 2 38.

BCL files were converted to fastq using Bcl2Fastq. Fastq reads were aligned to the mouse reference genome mm10 using the STAR aligner (*55*), and counts assigned to genes using the RSEM package (*56*). The R/Bioconductor package DESeq2 (*57*) was used to identify differentially expressed (DE) genes. The data were not normalized or pre-processed in any way, as DESeq2 expects raw un-normalized counts as input. Genes with a Benjamini and Hochberg–corrected *P* value < 0.1 were considered statistically significant.

#### Cytokine production in human MDDCs

Frozen human monocytes were thawed and allowed to recover overnight in DMEM [10% FBS, 1% p/s, IL-4 (50 ng/ml; Peprotech, 200-04), and GM-CSF (50 ng/ml; Peprotech, 300-03)] at 37°C and 5% CO_2_. The next day, media was changed to DMEM [10% FBS, 1% p/s, IL-4 (100 ng/ml), and GM-CSF (100 ng/ml)], and cells were differentiated over 7 days with a 50% media change every other day. On day 7, cells were seeded at 25,000 cells per well, and pH-stable DMEM was prepared as described above. On day 8, MDDCs were costimulated with LPS (100 ng/ml; Invivogen, tlrl-3pelps) and 0, 5, or 20 μM BRD5075 at pH 6.6, 7.2, and 7.6 for 3 hours. Supernatants were collected and TNF production was measured by ELISA (Invitrogen, 88-7346-88). Plates were read on BMG Labtech PHERAstar FSX. Data analysis was performed using GraphPad Prism.

### Quantification and statistical analysis

Statistical analysis was done using Prism (GraphPad Software) or Excel (Microsoft Office). Data represent mean ± SD.

## Supplementary Materials

### This PDF file includes:

Supplementary Text Figs. S1 to S5 Legends for data S1 to S4 References
