# Supplementary material for: Small-molecule probe for IBD risk variant GPR65 I231L alters cytokine signaling networks through positive allosteric modulation
Source: Sci Adv. 2024 Jul 19;10(29):eadn2339. doi: 10.1126/sciadv.adn2339 (PMC11259170; doi:10.1126/sciadv.adn2339)
Supplement: Supplementary file 1 — Supplementary Text Figs. S1 to S5 Legends for data S1 to S4 References [file sciadv.adn2339_sm.pdf]

Science Advances
AAAS

Supplementary Materials for

Small-molecule probe for IBD risk variant GPR65 I231L alters cytokine signaling networks through positive allosteric modulation

Ilona Neale *et al.*

Corresponding author: Ramnik J. Xavier, xavier@molbio.mgh.harvard.edu

Sci. Adv. **10**, eadn2339 (2024)
DOI: 10.1126/sciadv.adn2339

The PDF file includes:

Supplementary Text
Figs. S1 to S5
Legends for data S1 to S4
References

Other Supplementary Material for this manuscript includes the following:

Data S1 to S4

Supplementary Text

Chemistry Information

General methods

All air or moisture sensitive reactions were performed under positive pressure of nitrogen with oven dried glassware. Chemical reagents and anhydrous solvents were obtained from commercial sources and used as-is.

Flash chromatography purifications were performed using a CombiFlash Rf system (Biotage Isolera One) with pre-packed 40-60 µm Silica Gel (60 Å mesh) columns. Preparative purification was performed on a Gilson 281. The column used was a Phenomenex luna C18 150*25mm*10um at a flow rate of 25 mL/min. NMR spectra were measured on a Bruker AVANCE NEO 400MHz. Proton chemical shifts are reported in ppm (δ) referenced to the NMR solvent. Data are reported as follows: chemical shifts, multiplicity (s = singlet, d = doublet, t = triplet, dd = doublet of doublets, q=quartet, dt = doublet of triplets, ddd= doublet of doublet of doublets, m = multiplet), coupling constant(s) in Hz, integration. All NMR data were collected at 25°C. LC-MS was performed on a SHIMADZU LC-20AD Kinetex® EVO C18 2.1x30mm 5um. HPLC was performed on a SHIMADZU LC-20AD Kinetex C18 LC Column 3.0X50mm,2.6um. High resolution LC-MS (HRMS) was performed on an Agilent 1290+6545 QTOF in ESI+ mode.

BRD2813

Synthetic Scheme for BRD2813

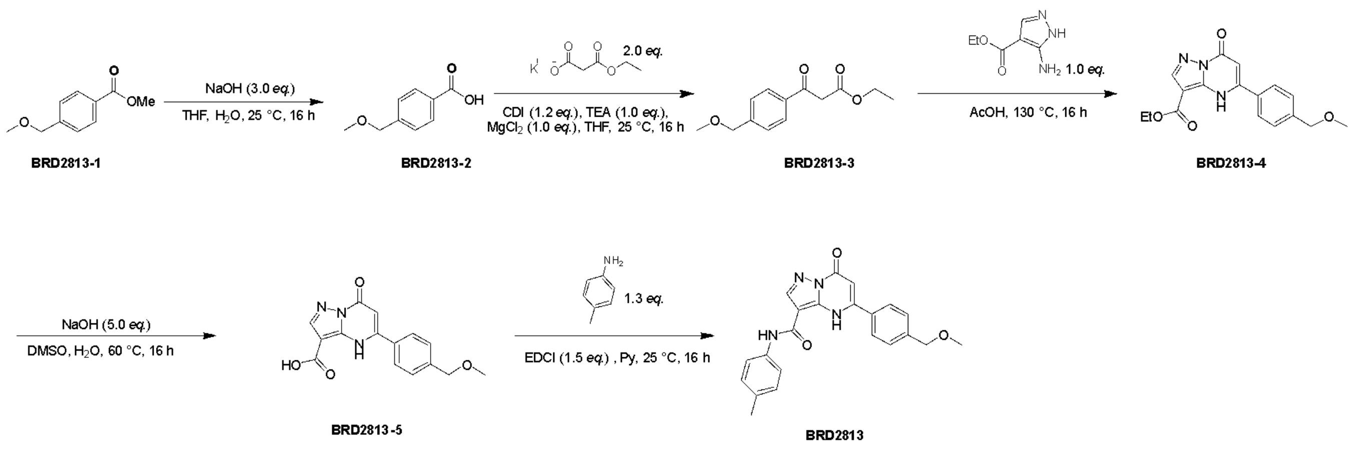

1.1 Procedure for preparation of BRD2813-2

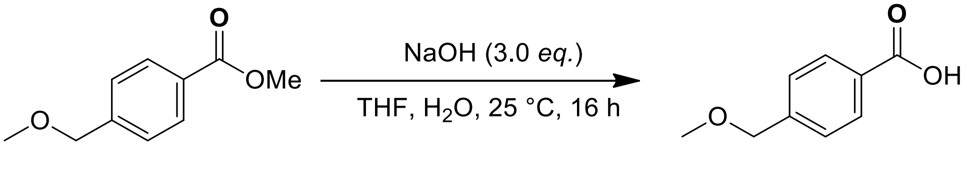

BRD2813-1 **BRD2813-2**

To a solution of methyl 4-(methoxymethyl)benzoate **BRD2813-1** (5.0 g, 27.75 mmol, 1.0 *eq.*) in THF (30 mL) and H_2O (10 mL), NaOH (3.33 g, 83.24 mmol, 3.0 *eq.*) was added. The mixture was stirred at 25 °C for 16 hours. The reaction mixture was concentrated under reduced pressure to remove THF. The residue was diluted with H_2O (30 mL) and adjusted to pH 3 by 5N HCl, filtered and the filter cake was washed with H_2O (100 mL) to give 4-(methoxymethyl)benzoic acid **BRD2813-2** (3.5 g, 21.06 mmol, 75.91% yield) as a white solid.

^{1}H NMR (400 MHz, DMSO-d_6) δ 12.90 (s, 1H), 7.92 (d, J = 8.0 Hz, 2H), 7.43 (d, J = 8.0 Hz, 2H), 4.49 (s, 2H), 3.32 (s, 3H).

1.2 Procedure for preparation of BRD2813-3

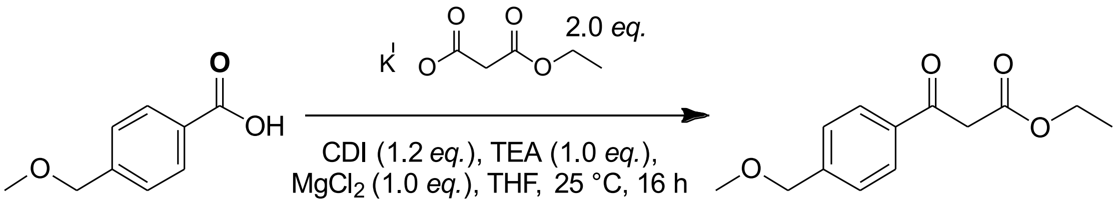

BRD2813-2 **BRD2813-3**

To a solution of 4-(methoxymethyl)benzoic acid **BRD2813-2** (3.0 g, 18.05 mmol, 1.0 *eq.*) in THF (30 mL), CDI (3.51 g, 21.66 mmol, 1.2 *eq.*) was added and the resulting mixture was stirred at 25 °C for 1 hr, before the addition of potassium 3-ethoxy-3-oxo-propanoate (6.15 g, 36.11 mmol, 2.0 *eq.*), $MgCl_2$ (1.72 g, 18.05 mmol, 740.90 uL, 1.0 *eq.*) and TEA (1.83 g, 18.05 mmol, 2.51 mL, 1.0 *eq.*). The resulting mixture was stirred at 25 °C for 15 hours. The reaction mixture was then filtered and the filtrate was concentrated. The residue was purified by flash silica gel chromatography (ISCO®; 80 g SepaFlash® Silica Flash Column, Eluent of 0~8% Ethyl acetate/Petroleum ether gradient @ 80 mL/min) to give ethyl 3-[4-(methoxymethyl)phenyl]-3-oxo-propanoate **BRD2813-3** (2.6 g, 11.00 mmol, 60.96% yield), obtained as colorless oil.

^{1}H NMR (400 MHz, DMSO-d_6) δ 7.94 (d, J = 8.0 Hz, 2H), 7.47 (d, J = 8.0 Hz, 2H), 4.50 (s, 2H), 4.17 - 4.08 (m, 4H), 3.32 (s, 3H), 1.17 (t, J = 7.2 Hz, 3H).

1.3 Procedure for preparation of BRD2813-4

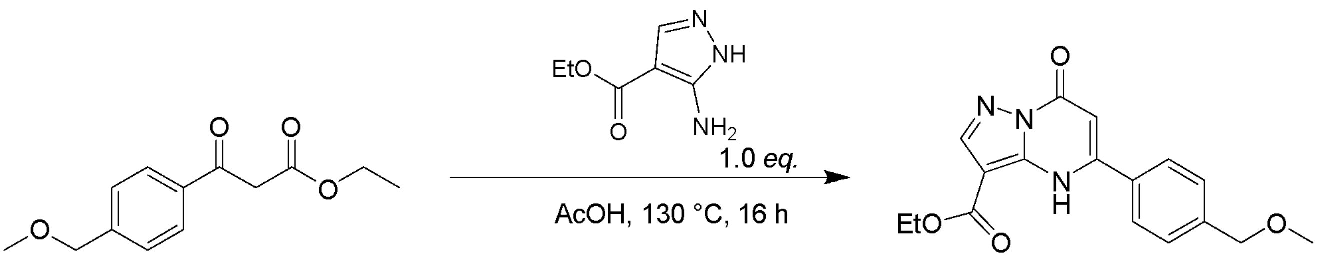

BRD2813-3 **BRD2813-4**

To a solution of ethyl 3-[4-(methoxymethyl)phenyl]-3-oxo-propanoate **BRD2813-3** (2.5 g, 10.58 mmol, 1.0 *eq.*) in AcOH (15 mL), ethyl 5-amino-1H-pyrazole-4-carboxylate (1.64 g, 10.58 mmol, 1.0 *eq.*) was added. The mixture was stirred at 130 °C for 16 hours. The reaction mixture was diluted with H_2O (100 mL), and EA (100 mL) was added. The mixture was extracted with EA 200 mL (100 mL × 2). The combined organic layers were dried over Na_2SO_4, filtered and concentrated under reduced pressure. The resulting residue was purified by flash silica gel chromatography (ISCO®; 40 g SepaFlash® Silica Flash Column, Eluent of 10~60% Ethyl acetate/Petroleum ether gradient @ 80 mL/min) to give ethyl 7-hydroxy-5-[4-(methoxymethyl)phenyl]pyrazolo[1,5-a]pyrimidine-3-carboxylate **BRD2813-4** (700 mg, 2.14 mmol, 20.21% yield) as a yellow oil.

^{1}H NMR (400 MHz, DMSO-d_6) δ 11.67 (s, 1H), 8.26 (s, 1H), 7.78 (d, J = 8.4 Hz, 2H), 7.52 (d, J = 8.0 Hz, 2H), 6.27 (s, 1H), 4.52 (s, 2H), 4.31 (q, J = 7.0 Hz, 2H), 3.34 (s, 3H), 1.34 (t, J = 7.2 Hz, 3H).

1.4 Procedure for preparation of BRD2813-5

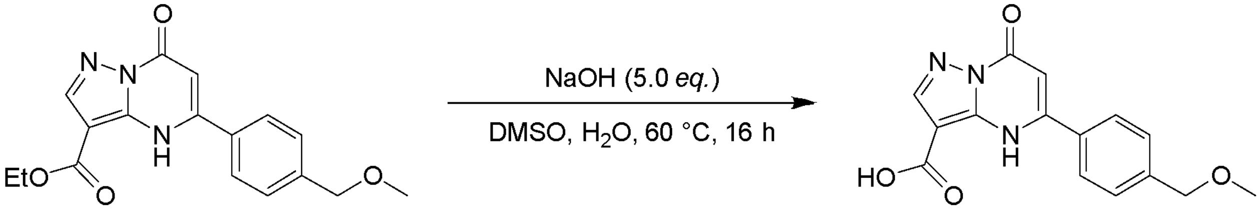

BRD2813-4 **BRD2813-5**

To a solution of ethyl 7-hydroxy-5-[4-(methoxymethyl)phenyl]pyrazolo[1,5-a]pyrimidine-3-carboxylate **BRD2813-4** (700 mg, 2.14 mmol, 1.0 *eq.*) in DMSO (2 mL) and H_2O (1 mL), NaOH (427.67 mg, 10.69 mmol, 5 *eq.*) was added and the mixture was stirred at 60 °C for 16 hours. The mixture was diluted with H_2O (3 mL) and adjusted to pH 3 by 5N HCl, filtered and washed with H_2O (10 mL), and concentrated under reduced pressure to give 7-hydroxy-5-[4-(methoxymethyl)phenyl]pyrazolo[1,5-a]pyrimidine-3-carboxylic acid **BRD2813-5** (530 mg, 1.77 mmol, 82.81% yield) as a light yellow solid.

^{1}H NMR (400 MHz, DMSO-d_6) δ 12.88 (s, 1H), 11.56 (s, 1H), 8.22 (s, 1H), 7.77 (d, J = 8.0 Hz, 2H), 7.51 (d, J = 8.0 Hz, 2H), 6.25 (s, 1H), 4.51 (s, 2H), 3.33 (s, 2H).

1.5 Procedure for preparation of BRD2813

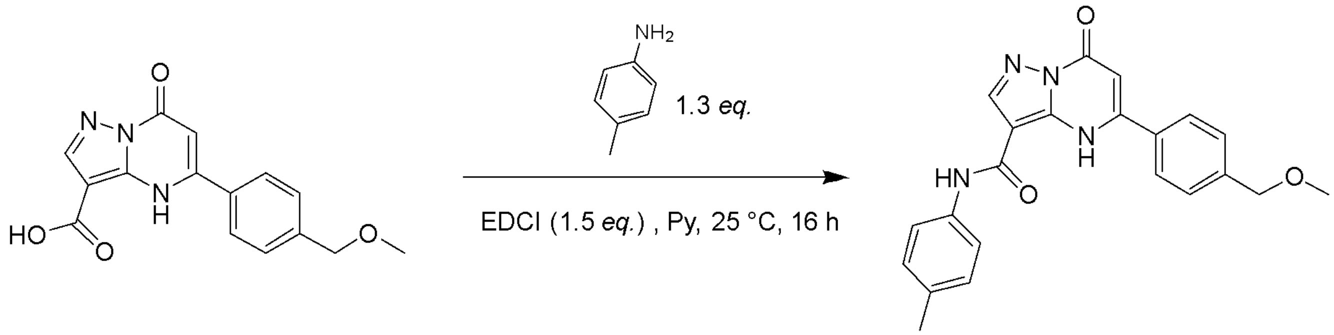

BRD2813-5 **BRD2813**

To a solution of 7-hydroxy-5-[4-(methoxymethyl)phenyl]pyrazolo[1,5-a]pyrimidine-3-carboxylic acid **BRD2813-5** (250 mg, 835.34 umol, 1.0 *eq.*) and 4-methylaniline (116.36 mg, 1.09 mmol, 119.59 uL, 1.3 *eq.*) in Py (2 mL), EDCI (240.20 mg, 1.25 mmol, 1.5 *eq.*) was added. The mixture was stirred at 25 °C for 16 hours. The reaction mixture was quenched by addition of H_2O **(**30 mL), and then filtered. The residue was purified by prep-HPLC (column: Welch Xtimate C18 150×25mm×5um;mobile phase: [water (ammonia hydroxide v/v)-ACN];B%: 14%-44%,2min) to give 7-hydroxy-5-[4-(methoxymethyl)phenyl]-N-(p-tolyl)pyrazolo[1,5-a]pyrimidine-3-carboxamide **BRD2813** (51.84 mg, 131.49 umol, 15.74% yield, 98.52% purity) as an off-white solid.

LCMS: t_R = 0.452 min, m/z: 389.2 $[M+H]^+$.

^{1}H NMR (400 MHz, DMSO-d$_6$) δ 11.01 (s, 1H), 8.22-8.17 (m, 1H),7.98 (d, J = 5.6 Hz, 2H), 7.60 (d, J = 8.4 Hz, 2H), 7.48 (d, J = 8.0 Hz, 2H), 7.16 (d, J = 8.0 Hz, 2H), 6.94 (s, 1H), 6.15 (s, 1H), 4.51 (s, 2H), 3.36 (s, 3H), 2.29 (s, 3H)

^{13}C NMR (100 MHz, DMSO-d$_6$) δ 160.67, 155.71, 149.70, 143.49, 141.99, 141.26, 136.15, 132.61, 130.60, 129.13, 127.90, 129.08, 120.11, 100.514, 96.57, 72.99, 57.77, 20.48

HRMS $C_{22}H_{20}N_4O_3$; calculated $(M+Na)^+$ = 411.1433; observed $(M+Na)^+$ = 411.1429

HPLC purity: 98.521%

BRD2166

Synthetic Scheme for BRD2166

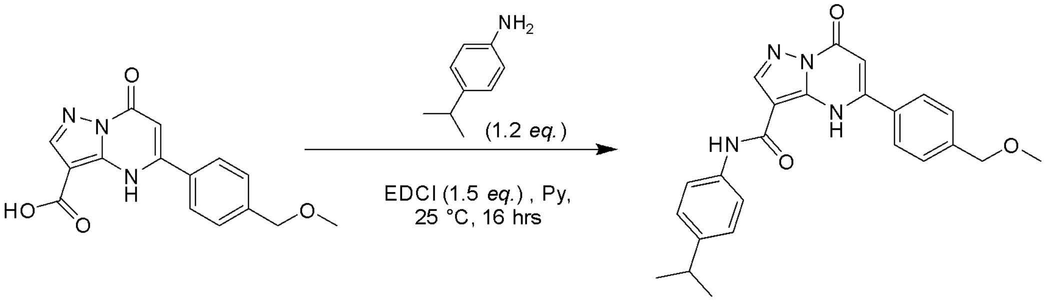

BRD2813-5 **BRD2166**

1.1 Procedure for preparation of BRD2166

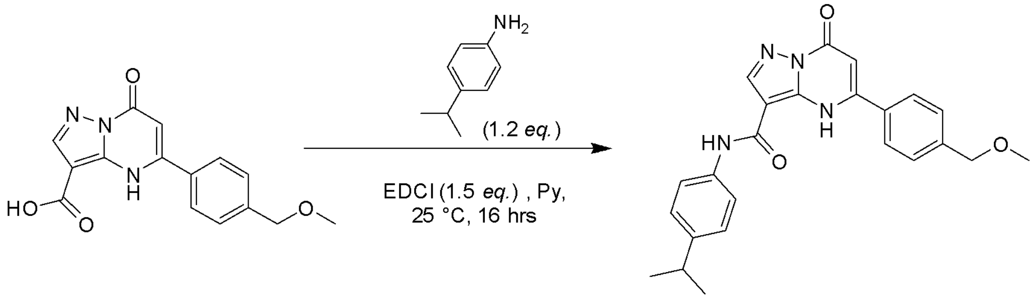

BRD2813-5 **BRD2166**

To a solution of 7-hydroxy-5-[4-(methoxymethyl)phenyl]pyrazolo[1,5-a]pyrimidine-3-carboxylic acid **BRD2813-5** (350 mg, 1.17 mmol, 1.0 *eq.*) and 4-isopropylaniline (189.74 mg, 1.40 mmol, 199.73 uL, 1.2 *eq.*) in Py (6 mL), EDCI (336.28 mg, 1.75 mmol, 1.5 *eq.*) was added. The mixture was stirred at 25 °C for 16 hours. The reaction mixture was quenched by adding H_2O (30.0 mL) at 25 °C, and then diluted with EA 30.0 mL and extracted with 150.0 mL EA (50.0 mL × 3). The combined organic layers were washed with brine 100.0 mL (50.0 mL × 2), dried over Na_2SO_4, filtered and concentrated under reduced pressure. The residue was purified by prep-HPLC (column: Phenomenex C_{18} 250×50mm×10um;mobile phase: [water (ammonia hydroxide v/v)-ACN];B%: 15%) to give 7-hydroxy-N-(4-isopropylphenyl)-5-[4-(methoxymethyl)phenyl] pyrazolo[1,5-a]pyrimidine-3-carboxamide **BRD2166** (247.42 mg, 580.13 umol, 49.61% yield, 97.65% purity) as a yellow solid.

LCMS: t_R = 0.513 min, m/z: 417.4 $[M+H]^+$.

^{1}H NMR (400 MHz, DMSO-*d₆*) δ 10.47 (s, 1H), 8.33 (s, 1H), 7.94 (d, *J* = 4.8 Hz, 2H), 7.64 (d, *J* = 8.4 Hz, 2H), 7.51 (d, *J* = 8.0 Hz, 2H), 7.23 (d, *J* = 8.4 Hz, 2H), 7.09 (s, 1H), 6.19 (s, 1H), 4.52 (s, 2H), 3.37 (s, 3H), 3.10 (s, 3H), 3.10-2.86 (m, 1H), 1.23 (d, *J* = 6.8 Hz, 6H).

^{13}C NMR (101 MHz, DMSO-d_6) δ 160.94, 157.64, 156.93, 155.77, 149.82, 143.54, 142.05, 139.33, 137.80, 136.43, 130.63, 127.79, 126.61, 120.03, 118.55, 100.86, 96.58, 92.00, 73.12, 57.70, 32.89, 23.99.

HRMS $C_{24}H_{24}N_4O_3$; calculated $(M+H)^+$ = 417.1921; observed $(M+H)^+$ = 417.1925

HPLC purity: 97.652%

BRD5928

Synthetic Scheme for BRD5928

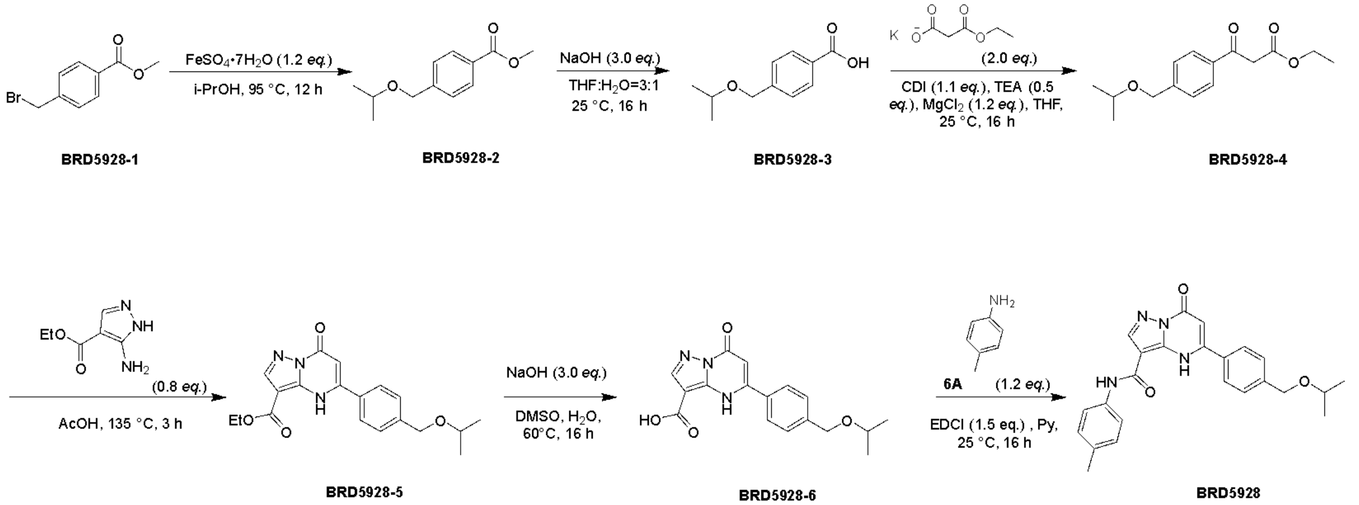

1.1 Procedure for preparation of BRD5928-2

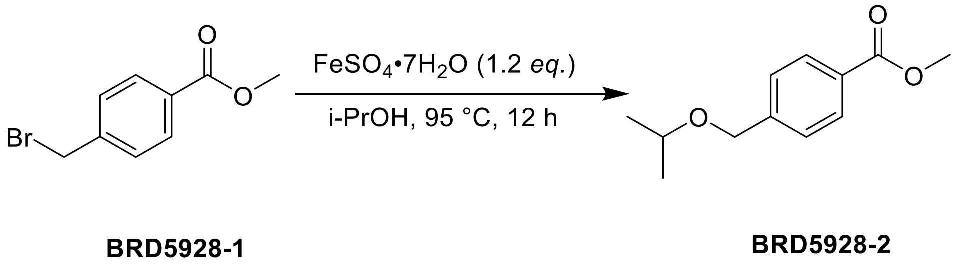

To a solution of methyl, 4-(bromomethyl) benzoate **BRD5928-1** (40.0 g, 174.62 mmol, 1.0 *eq.*) in i-PrOH (300 mL) $FeSO_4 \cdot 7H_2O$ (58.26 g, 209.54 mmol, 1.2 *eq.*) was added. The mixture was refluxed at 95 °C for 12 hours. The reaction mixture was filtered under reduced pressure to remove residue and concentrated the solvent to give a residue. The residue was purified by flash silica gel chromatography (ISCO®; 330 g SepaFlash® Silica Flash Column, Eluent of 0~8% Ethyl acetate/Petroleum ether gradient @ 120 mL/min) to give methyl 4-(isopropoxymethyl) benzoate **BRD5928-2** (21.5 g, 103.24 mmol, 59.12% yield) as colorless oil.

^{1}H NMR (400 MHz, DMSO-d_6) δ 7.94 (d, J = 8.0 Hz, 2H), 7.46 (d, J = 8.4 Hz, 2H), 4.55 (s, 2H), 3.85 (s, 3H), 3.69 - 3.63 (m, 1H), 1.16 (d, J = 6.0 Hz, 6H).

1.2 Procedure for preparation of BRD5928-3

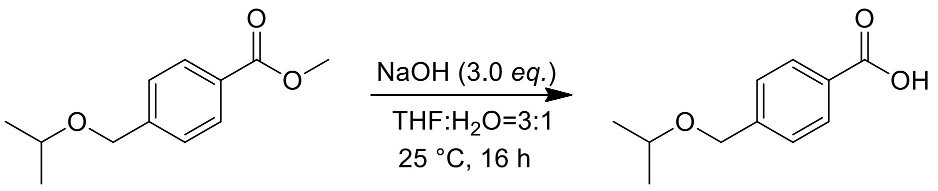

BRD5928-2 **BRD5928-3**

To a solution of methyl 4-(isopropoxymethyl) benzoate **BRD5928-2** (21.5 g, 103.24 mmol, 1.0 *eq.*) in THF (300 mL) and H_2O (100 mL), NaOH (12.39 g, 309.72 mmol, 3.0 *eq.*) was added. The mixture was stirred at 25 °C for 16 hours. The mixture was diluted with H_2O (50 mL), then the solution was adjusted to pH 3 using 1M HCl. The precipitated solids were filtered to give 4-(isopropoxymethyl) benzoic acid **BRD5928-3** (20 g, 102.97 mmol, 99.74% yield) as a yellow solid.

1.3 Procedure for preparation of BRD5928-4

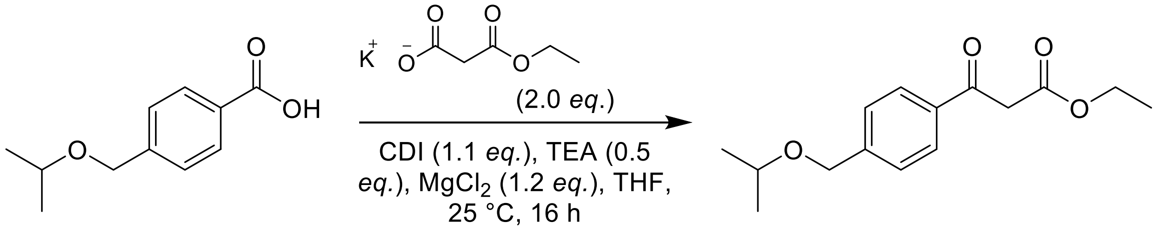

BRD5928-3 **BRD5928-4**

A mixture of 4-(isopropoxymethyl) benzoic acid **BRD5928-3** (5.0 g, 25.74 mmol, 1.0 *eq.*) and CDI (5.01 g, 30.89 mmol, 1.1 *eq.*) in THF (50 mL) was stirred at 25 °C for 1 hour. $MgCl_2$ (2.94 g, 30.89 mmol, 1.27 mL, 1.2 *eq.*), TEA (1.30 g, 12.87 mmol, 1.79 mL, 0.5 *eq.*) and potassium 3-ethoxy-3-oxo-propanoate (6.57 g, 38.61 mmol, 2.0 *eq.*) were then added into the solution and stirred for 15 hours at 25°C. The reaction mixture was quenched by addition of brine (30 mL) and extracted with EA (50 mL × 2). The combined organic layers were dried over anhydrous Na_2SO_4 and concentrated under reduced pressure to give a residue. The residue was purified by flash silica gel chromatography (ISCO®; 40 g SepaFlash® Silica Flash Column, Eluent of 0~20% Ethyl acetate/Petroleum ether gradient @ 100 mL/min) to give ethyl 3-[4-(isopropoxymethyl) phenyl]-3-oxo-propanoate **BRD5928-4** (1.0 g, 3.78 mmol, 14.70% yield) as colorless oil.

^{1}H NMR (400 MHz, DMSO-*d*$_6$) δ 7.93 (d, *J* = 8.0 Hz, 2H), 7.47 (d, *J* = 8.4 Hz, 2H), 4.55 (s, 2H), 4.17 (s, 2H), 4.11 (q, *J* = 7.2 Hz, 2H), 3.69 - 3.64 (m, 1H), 1.19 - 1.14 (m, 9H).

1.4 Procedure for preparation of BRD5928-5

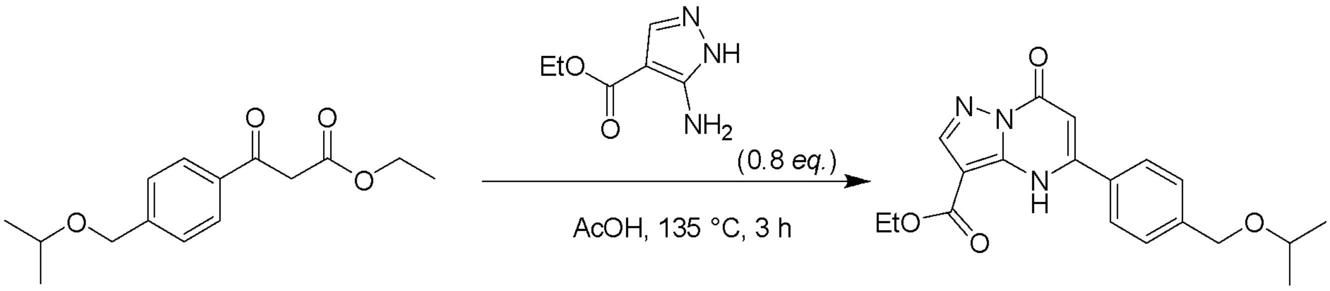

BRD5928-4 **BRD5928-5**

A solution of ethyl 3-[4-(isopropoxymethyl)phenyl]-3-oxo-propanoate **BRD5928-4** (300 mg, 1.14 mmol, 1.0 *eq.*) and ethyl 3-amino-1*H*-pyrazole-4-carboxylate (140.88 mg, 908.00 umol, 0.8 *eq.*) in AcOH (0.5 mL) was stirred at 135 °C for 3 hours. After cooling to room temperature, EA (100 mL) and H_2O (100mL) were added, and reaction mixture was extracted with EA (100 mL × 2). The combined organic layers were dried over anhydrous Na_2SO_4, filtered and concentrated under reduced pressure to give a residue. The residue was purified by flash silica gel chromatography (ISCO®; 40 g SepaFlash® Silica Flash Column, Eluent of 0~50% Ethyl acetate/Petroleum ether gradient @ 80 mL/min) to give ethyl 7-hydroxy-5-[4-(isopropoxymethyl) phenyl] pyrazolo [1, 5-a] pyrimidine-3-carboxylate **BRD5928-5** (80 mg, 225.11 umol, 19.83% yield) as a yellow oil.

^{1}H NMR (400 MHz, DMSO-*d*$_6$) δ 11.67 (s, 1H), 8.22 (s, 1H), 7.82 (d, *J* = 8.0 Hz, 2H), 7.50 (d, *J* = 8.0 Hz, 2H), 6.25 (s, 1H), 4.56 (s, 2H), 4.34 - 4.27 (m, 2H), 3.72 - 3.64 (m, 1H), 1.35 (t, *J* = 7.2 Hz, 3H), 1.18 (d, *J* = 6.0 Hz, 6H)

1.5 Procedure for preparation of BRD5928-6

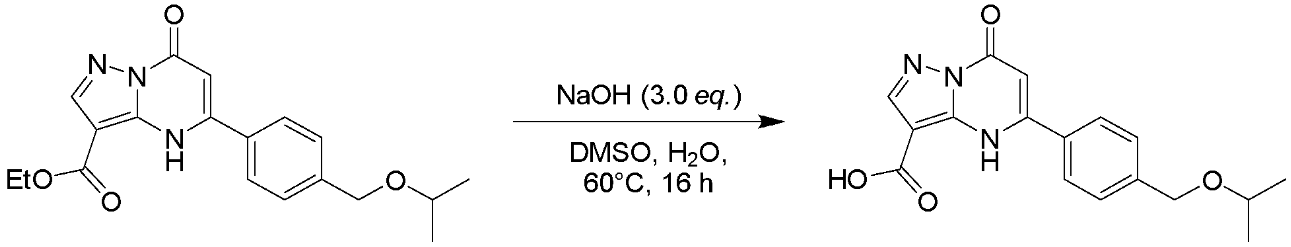

BRD5928-5 **BRD5928-6**

To a solution of ethyl 7-hydroxy-5-[4-(isopropoxymethyl)phenyl]pyrazolo[1,5-a]pyrimidine-3-carboxylate **BRD5928-5** (1.0 g, 2.81 mmol, 1.0 *eq.*) in DMSO (7.5 mL) and H_2O (2.5 mL), NaOH (337.64 mg, 8.44 mmol, 3.0 *eq.*) was added. The mixture was stirred at 60 °C for 16 hours . The mixture was diluted with H_2O (20 mL) and adjusted to pH 3 by 1M HCl. The precipitated solids are filtered to give 7-hydroxy-5-[4-(isopropoxymethyl)phenyl]pyrazolo[1,5-a]pyrimidine-3-carboxylic acid **BRD5928-6** (686 mg, 2.10 mmol, 74.48% yield) as a white solid.

1.6 Procedure for preparation of BRD5928

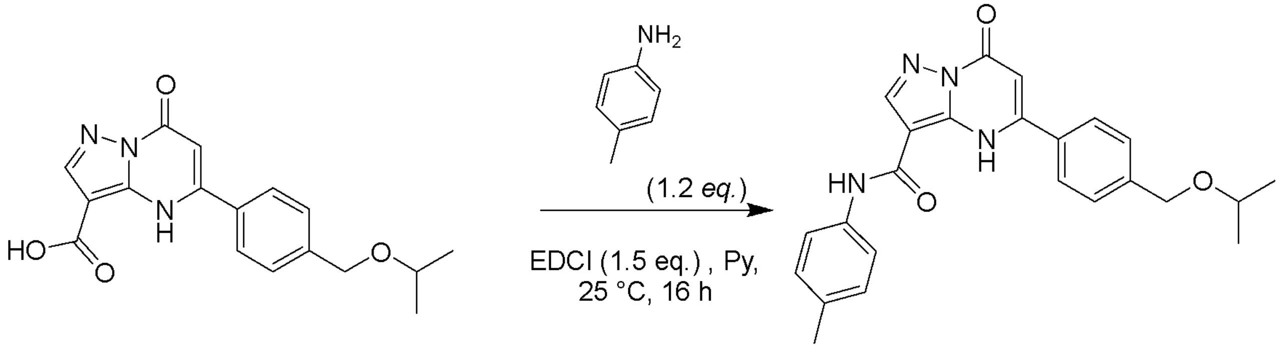

BRD5928-6 **BRD5928**

To a solution of 7-hydroxy-5-[4-(isopropoxymethyl) phenyl] pyrazolo [1, 5-a] pyrimidine-3-carboxylic acid **BRD5928-6** (200 mg, 611.00 umol, 1.0 *eq.*), 4-methylaniline (78.56 mg, 733.20 umol, 80.74 uL, 1.2 *eq.*) in pyridine (3 mL), EDCI (175.69 mg, 916.50 umol, 1.5 *eq.*) was added. The mixture was stirred at 25 °C for 16 hours. The reaction mixture was filtered and the solvents of the filtrate was removed under reduced pressure to give a residue. The residue was purified by prep-HPLC (column: Welch Xtimate C18 150 × 25mm × 5um; mobile phase: [water (NH_3H_2O)-ACN]; B%: 15%-45%, 8min) to give 7-hydroxy-5-[4-(isopropoxymethyl)phenyl]-N-(p-tolyl)pyrazolo[1,5-a]pyrimidine-3-carboxamide **BRD5928** (71.78 mg, 169.35 umol, 27.72% yield, 98.26% purity) as a pink solid.

LCMS: t_R = 0.476 min, m/z: 417.1 $[M+H]^+$.

^{1}H NMR (400 MHz, DMSO-d_6) δ 11.01 (s, 1H), 8.04 (d, J = 6.0 Hz, 2H), 7.60 (d, J = 7.6 Hz, 2H), 7.47 (d, J = 6.8 Hz, 1H), 7.21 - 7.16 (m, 2H), 7.08 (s, 1H), 6.95 (s, 1H), 6.12 (s, 1H), 4.54 (s, 2H), 3.71 - 3.67 (m, 1H), 2.28 (s, 3H), 1.18 (d, J = 6.0 Hz, 6H)

^{13}C NMR (101 MHz, DMSO) δ 161.61, 146.78, 145.23, 142.72, 140.98, 131.82, 130.96, 129.88, 128.55, 127.99, 126.95, 124.84, 118.98, 101.58, 92.31, 70.98, 69.17, 22.55, 20.92.

HRMS $C_{24}H_{24}N_4O_3$; calculated $(M+H)^+$ = 417.1921; observed $(M+H)^+$ = 417.1926

HPLC purity: 99.379%

BRD5059

Synthetic Scheme for BRD5059

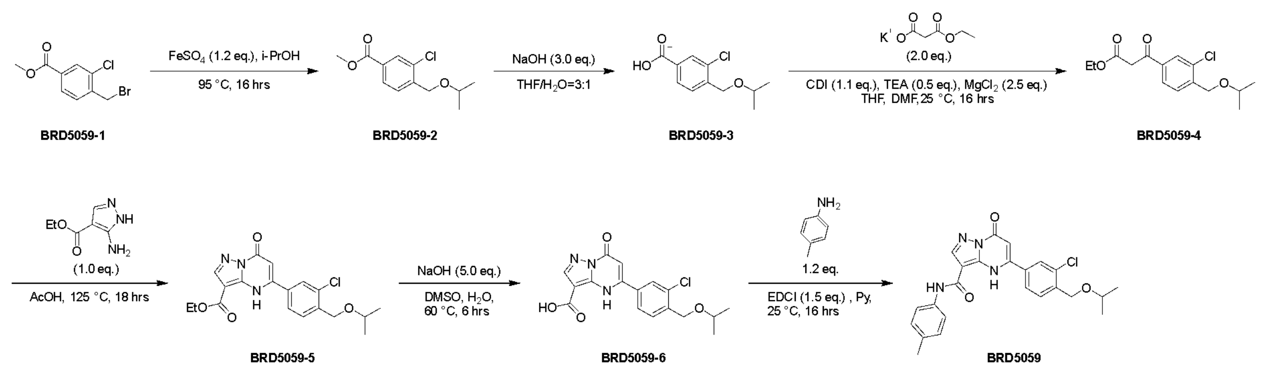

1.1 Procedure for preparation of BRD5059-2

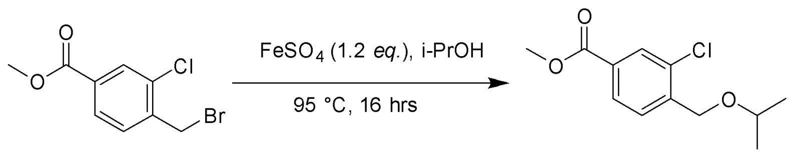

BRD5059-1 **BRD5059-2**

To a solution of methyl 4-(bromomethyl)-3-chloro-benzoate **BRD5059-1** (4.00 g, 15.18 mmol, 1.0 *eq.*) in i-PrOH (200 mL), $FeSO_4 \cdot 7H_2O$ (5.06 g, 18.22 mmol, 1.2 *eq.*) was added. The mixture was stirred at 95 °C for 16 hours. The reaction mixture was added into H_2O (25 mL), and then diluted with EA 50 mL and extracted with EA 100 mL (50 mL × 2). The combined organic layers were dried over Na_2SO_4, filtered and concentrated under reduced pressure to give a residue. The crude product was purified by reversed-phase HPLC (0.1 % FA condition) to give methyl 3-chloro-4-(isopropoxymethyl) benzoate **BRD5059-2** (1.00 g, 4.12 mmol, 27.14% yield) as colorless oil.

^{1}H NMR (400 MHz, DMSO-*d_6*) δ 7.93 - 7.90 (m, 2H), 7.64 (d, *J* = 8.0 Hz, 1H), 4.57 (s, 2H), 3.86 (s, 3H), 3.76 - 3.67 (m, 1H), 1.18 (d, *J* = 6.0 Hz, 6H)

1.2 Procedure for preparation of BRD5059-3

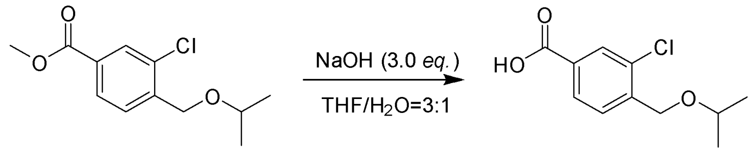

BRD5059-2 **BRD5059-3**

To a solution of methyl 3-chloro-4-(isopropoxymethyl) benzoate **BRD5059-2** (980.0 mg, 4.04 mmol, 1.0 *eq.*) in THF (12 mL) and H_2O (4 mL), NaOH (484.52 mg, 12.11 mmol, 3.0 *eq.*) was added. The mixture was stirred at 25°C for 16 hours. The reaction mixture was concentrated under reduced pressure, after which H_2O (5.0 ml) was added and the solution was adjusted to pH 3~4 with 1M HCl. The resulting suspension was filtered to give 3-chloro-4-(isopropoxymethyl) benzoic acid **BRD5059-3** (930.0 mg, 4.03 mmol, 99.71% yield, 99 % purity) as a white solid.

^{1}H NMR (400 MHz, DMSO-d_6) δ 13.26 (s, 1H), 7.92 - 7.89 (m, 2H), 7.63 (d, J = 7.6 Hz, 1H), 4.58 (s, 2H), 3.76-3.70 (m, 1H), 1.19 (d, J = 6.4 Hz, 6H).

1.3 Procedure for preparation of BRD5059-4

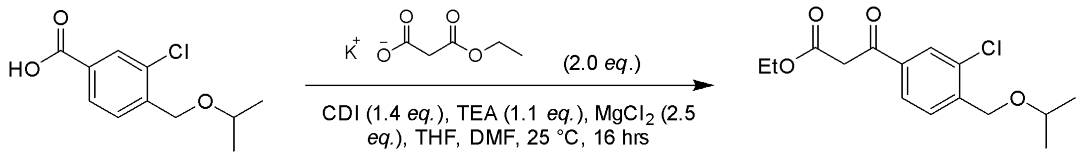

BRD5059-3 **BRD5059-4**

To a solution of 3-chloro-4-(isopropoxymethyl) benzoic acid **BRD5059-3** (900.0 mg, 3.94 mmol, 1.0 *eq.*) in THF (15.0 mL) and DMF (3.0 mL), CDI (893.45 mg, 5.51 mmol, 1.4 *eq.*) and $MgCl_2$ (562.09 mg, 5.90 mmol, 242.28 uL, 1.5 *eq.*) were added, the mixture was stirred at 25 °C for 4 hours under N_2. Potassium 3-ethoxy-3-oxo-propanoate (1.34 g, 7.87 mmol, 2.0 *eq.*), $MgCl_2$ (374.73 mg, 3.94 mmol, 161.52 uL, 1.0 *eq.*) and TEA (438.08 mg, 4.33 mmol, 602.59 uL, 1.1 *eq.*) were added, and the mixture was stirred at 25 °C for 12 hours under N_2. The reaction mixture was added into H_2O (25.0 mL), diluted with EA (50.0 mL) and extracted with EA 150.0 mL (50.0 mL × 3). The combined organic layers were dried over Na_2SO_4, filtered and concentrated under reduced pressure to give a residue. The residue was purified by flash silica gel chromatography (ISCO®; 25.0 g SepaFlash® Silica Flash Column, Eluent of 0~8% Ethyl acetate/Petroleum ether gradient @ 60 mL/min) to give 3-[3-chloro-4-(isopropoxymethyl) phenyl]-3-oxo-propanoate **BRD5059-4** (946.0 mg, 3.09 mmol, 78.60% yield, 97.7% purity) as light yellow oil.

^{1}H NMR (400 MHz, DMSO-d_6) δ 7.96 (d, J = 1.6 Hz, 1H), 7.93 – 7.90 (m, 1H), 7.66 (d, J = 8.0 Hz, 1H), 4.58 (s, 2H), 4.21 (s, 2H), 4.12 (q, J = 7.2 Hz, 2H), 3.76 - 3.71 (m, 1H), 1.19 - 1.17 (m, 9H).

1.4 Procedure for preparation of BRD5059-5

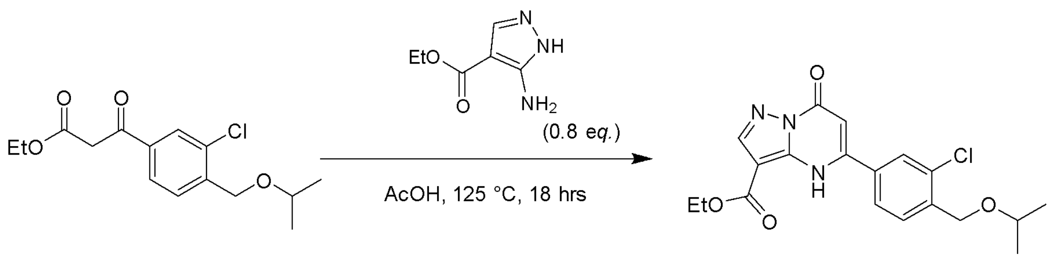

BRD5059-4 **BRD5059-5**

Ethyl 3-[3-chloro-4-(isopropoxymethyl)phenyl]-3-oxo-propanoate **BRD5059-4** (940.0 mg, 3.15 mmol, 1.0 *eq.*) and ethyl 5-amino-1H-pyrazole-4-carboxylate (390.53 mg, 2.52 mmol, 0.8 *eq.*) were dissolved in AcOH (10.0 mL) and was stirred at 125 °C for 18 hours under N_2. The reaction mixture was added into H_2O (25.0 mL), and then diluted with EA 50.0 mL and extracted with EA 150.0 mL (50.0 mL × 3). The combined organic layers were dried over Na_2SO_4, filtered and concentrated under reduced pressure to give a residue. The residue was purified by flash silica gel chromatography (ISCO®; 25.0 g SepaFlash® Silica Flash Column, Eluent of 0~36 % Ethyl acetate/Petroleum ether gradient @ 80 mL/min) to give 5-[3-chloro-4-(isopropoxymethyl)phenyl]-7-hydroxy-pyrazolo[1,5-a]pyrimidine-3-carboxylate **BRD5059-5** (400.0 mg, 1.03 mmol, 32.61% yield) as black brown oil.

1.5 Procedure for preparation of BRD5059-6

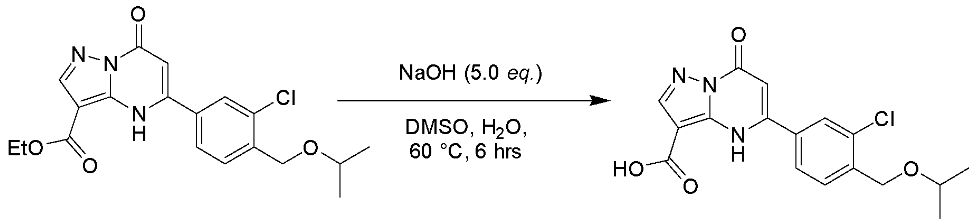

BRD5059-5 **BRD5059-6**

To a solution of ethyl 5-[3-chloro-4-(isopropoxymethyl) phenyl]-7-hydroxy-pyrazolo [1, 5-a]pyrimidine-3-carboxylate **BRD5059-5** (380.0 mg, 974.78 umol, 1.0 *eq.*) in DMSO (2.0 mL) and H_2O (2.0 mL), NaOH (194.94 mg, 4.87 mmol, 5.0 *eq.*) was added. The mixture was stirred at 60 °C for 6 hours. H_2O (5.0 ml) was added to the reaction mixture, and the resulting mixture was adjusted to pH 3~4 with 1M HCl. The reaction mixture was concentrated under reduced pressure to give 5-[3-chloro-4-(isopropoxymethyl)phenyl]-7-hydroxy-pyrazolo[1,5-a]pyrimidine-3-carboxylic acid **BRD5059-6** (75.0 mg, 181.81 umol, 18.65% yield, 87.7% purity) as a yellow solid.

^{1}H NMR (400 MHz, DMSO-d_6) δ 12.89 (s, 1H), 11.79 (s, 1H), 8.23 (s, 1H), 7.84 (d, J = 1.6 Hz, 1H), 7.75 – 7.73 (m, 1H), 7.67 - 7.64 (m, 1H), 6.27 (s, 1H), 4.60 (s, 2H), 3.77 – 3.71 (m, 1H), 1.20 (d, J = 6.0 Hz, 6H)

1.6 Procedure for preparation of BRD5059

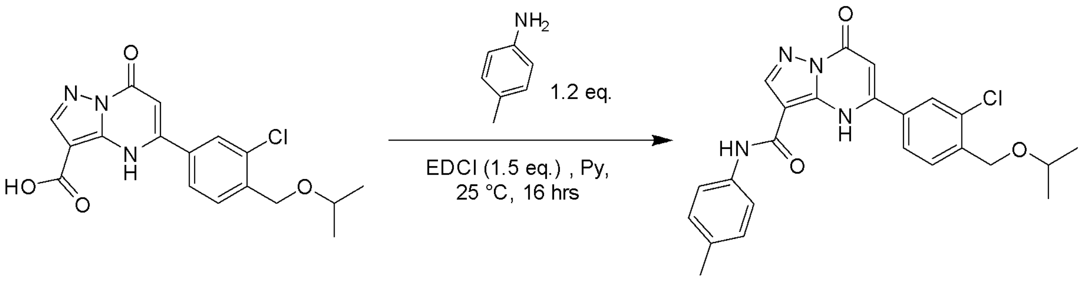

BRD5059-6 **BRD5059**

To a solution of 5-[3-chloro-4-(isopropoxymethyl)phenyl]-7-hydroxy-pyrazolo[1,5-a]pyrimidine-3-carboxylic acid **BRD5059-6** (200.0 mg, 552.82 umol, 1.0 *eq.*) and 4-methylaniline (71.08 mg, 663.39 umol, 73.06 uL, 1.2 *eq.*) in Py (3.0 mL), EDCI (158.97 mg, 829.24 umol, 1.5 *eq.*) was added. The mixture was stirred at 25 °C for 16 hours. The reaction mixture was quenched by addition H_2O 30 mL at 25 °C, and then diluted with EA 30 mL and extracted with EA 150 mL (50 mL × 3). The combined organic layers were washed with brine 100 mL (50 mL × 2), dried over Na_2SO_4, filtered and concentrated under reduced pressure to give a residue. The residue was purified by prep-HPLC (column: Phenomenex C_{18} 150×25mm×10um;mobile phase: [water (NH_4HCO_3)-ACN]; B%: 38%-68%,8min) to give 5-[3-chloro-4-(isopropoxymethyl)phenyl]-7-hydroxy-N-(p-tolyl)pyrazolo[1,5-a]pyrimidine-3-carboxamide **BRD5059** (50.63 mg, 111.86 umol, 20.23% yield, 99.624% purity) as a yellow solid.

LCMS*:* t_R = 0.542 min, m/z: 451.2 $[M+H]^+$.

^{1}H NMR (400 MHz, DMSO-*d*$_6$) δ 10.67 (s, 1H), 8.21 (s, 1H), 8.11 (s, 1H), 7.94 (d, *J* = 8.0 Hz, 1H), 7.66 - 7.62 (m, 3H), 7.16 (d, *J* = 8.0 Hz, 2H), 6.19 (s, 1H), 4.62 (s, 2H), 3.82 - 3.73 (m, 1H), 2.29 (s, 3H), 1.22 (d, *J* = 6.0 Hz, 6H).

^{13}C NMR (101 MHz, DMSO-*d*$_6$) δ 161.01, 158.96, 155.29, 154.99, 149.89, 142.35, 139.65, 137.30, 132.21, 131.34, 129.38, 127.00, 125.16, 124.40, 118.25, 101.24, 92.09, 71.19, 66.31, 22.03, 20.42.

HRMS $C_{24}H_{23}ClN_4O_3$; calculated $(M+H)^+$ = 451.1531; observed $(M+H)^+$ = 451.1525

HPLC purity: 99.720%

BRD5067

Synthetic Scheme for BRD5067

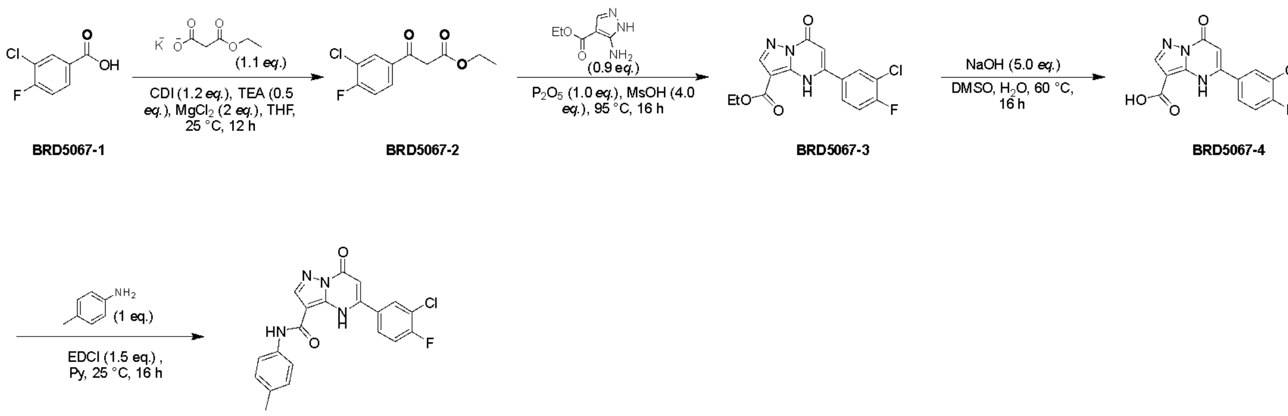

BRD5067-1 BRD5067-2 BRD5067-3 BRD5067-4

BRD5067

1.1 Procedure for preparation of BRD5067-2

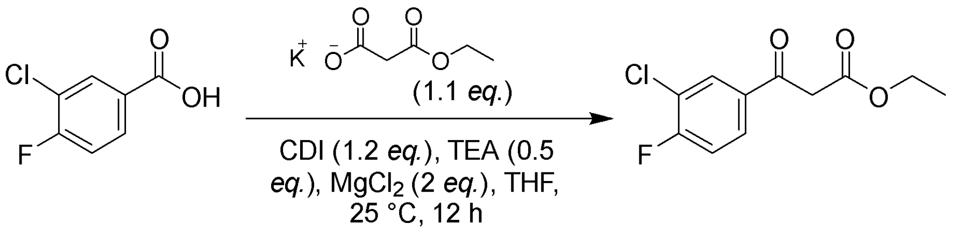

BRD5067-1 **BRD5067-2**

A solution of 3-chloro-4-fluoro-benzoic acid **BRD5067-1** (4 g, 22.92 mmol, 1 *eq.*) and CDI (4.46 g, 27.50 mmol, 1.2 *eq.*) in THF (20 mL) was stirred for 3 hrs, followed by addition of a solution of potassium 3-ethoxy-3-oxo-propanoate (4.29 g, 25.21 mmol, 1.1 *eq.*), TEA (1.16 g, 11.46 mmol, 1.59 mL, 0.5 *eq.*) and $MgCl_2$ (4.36 g, 45.83 mmol, 1.88 mL, 2 *eq.*) in THF. The mixture was stirred at 25 °C for 12 hours. The reaction mixture was partitioned between EA (500 mL) and H_2O (300 mL). The organic phase was separated, washed with saturated brine 90 mL (30 mL × 3), then filtered and concentrated under reduced pressure to give a residue. The residue was purified by flash silica gel chromatography (ISCO®; 40 g SepaFlash® Silica Flash Column, Eluent of 10~35% Ethyl acetate/Petroleum ether gradient @100mL/min) to give ethyl 3-(3-chloro-4-fluoro-phenyl)-3-oxo-propanoate **BRD5067-2** (3.6 g, 14.72 mmol, 64.22% yield) as a brown oil.

^{1}H NMR (400 MHz, DMSO-*d_6*) δ 8.16 (dd, *J*=7.2, 1.6 Hz, 1H), 7.96 (ddd, *J*=8.66, 4.72, 2.13 Hz, 1H), 7.62 - 7.55 (m, 1H), 4.23 (s, 2H), 4.12 (q, *J*=7.2 Hz, 2H), 1.16 (t, *J*=7.2 Hz, 3H)

^{19}F NMR (377 MHz, DMSO-*d_6*) δ -108.357 (s, 1F).

1.2 Procedure for preparation of BRD5067-3

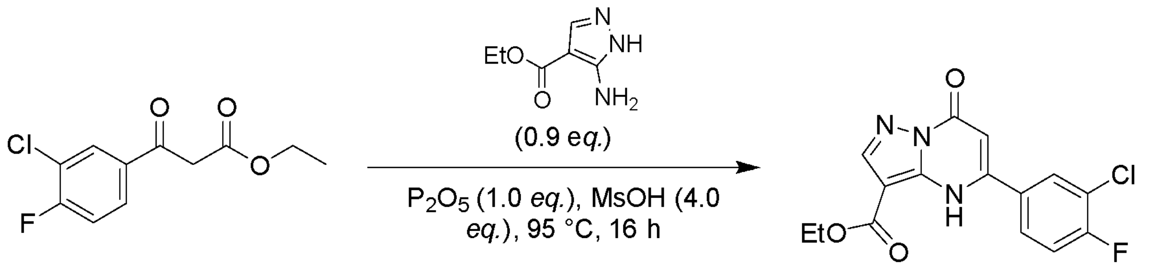

BRD5067-2 **BRD5067-3**

A mixture of ethyl 3-(3-chloro-4-fluoro-phenyl)-3-oxo-propanoate **BRD5067-2** (2.6 g, 10.63 mmol, 1 *eq.*), ethyl 5-amino-1*H*-pyrazole-4-carboxylate (1.48 g, 9.56 mmol, 0.9 *eq.*), P_2O_5 (1.51 g, 10.63 mmol, 655.88 uL, 1 *eq.*), MsOH (4.09 g, 42.51 mmol, 3.03 mL, 4 *eq.*) was degassed and purged with N_2 for 3 times, and then the mixture was stirred at 95 °C for 16 hours under N_2 atmosphere. The reaction mixture was partitioned between EA (300 mL) and H_2O (100mL), filtered and concentrated under reduced pressure to give ethyl 5-(3-chloro-4-fluoro-phenyl)-7-hydroxy-pyrazolo[1,5-a] pyrimidine-3-carboxylate **BRD5067-3** (500 mg, crude) as an off-white solid.

^{1}H NMR (400 MHz, DMSO-d_6) δ 12.04 (s, 1H), 8.35 - 8.15 (m, 1H), 8.09 - 7.95 (m, 1H), 7.87 - 7.74 (m, 1H), 7.70 - 7.55 (m, 1H), 6.34 - 6.21 (m, 1H), 4.44-4.15 (m, 2H), 1.43 - 1.25 (m, 3H)

^{19}F NMR (377 MHz, DMSO-d_6) δ = -112.897 (s, 1F).

1.3 Procedure for preparation of BRD5067-4

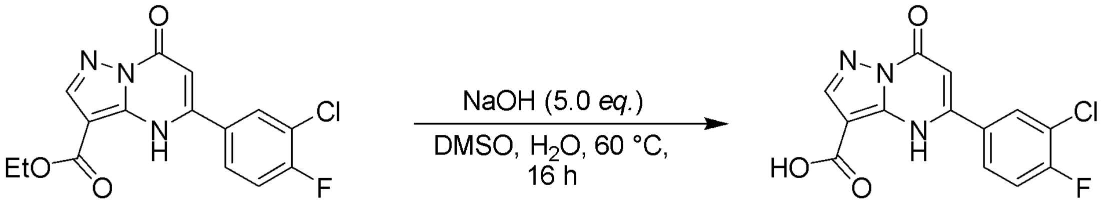

BRD5067-3 **BRD5067-4**

A mixture of ethyl 5-(3-chloro-4-fluoro-phenyl)-7-hydroxy-pyrazolo[1,5-a]pyrimidine-3-carboxylate **BRD5067-3** (1 g, 2.98 mmol, 1 *eq.*) and NaOH (595.74 mg, 14.89 mmol, 5 *eq.*) in DMSO (6 mL) and H_2O (3 mL) was degassed and purged with N_2 for 3 times, and then the mixture was stirred at 60 °C for 16 hours under N_2 atmosphere. The mixture was diluted with H_2O (50 mL) and was adjusted to pH 3 using 1M HCl. The precipitated solids are filtered to give 5-(3-chloro-4-fluoro-phenyl)-7-hydroxy-pyrazolo[1,5-a]pyrimidine-3-carboxylic acid **BRD5067-4** (1.2 g, crude) as a

white solid.

^{1}H NMR (400 MHz, DMSO-*d$_6$*) *δ* 11.87 (m, 1H), 8.24 (s, 1H), 8.00 (dd, *J*=7.2, 2.4 Hz, 1H), 7.83 - 7.72 (m, 1H), 7.60 (t, *J*=8.8 Hz, 1H), 6.24 (s, 1H)

^{19}F NMR (377 MHz, DMSO-*d$_6$*) *δ* = -113.032 (s, 1F).

1.4 Procedure for preparation of BRD5067

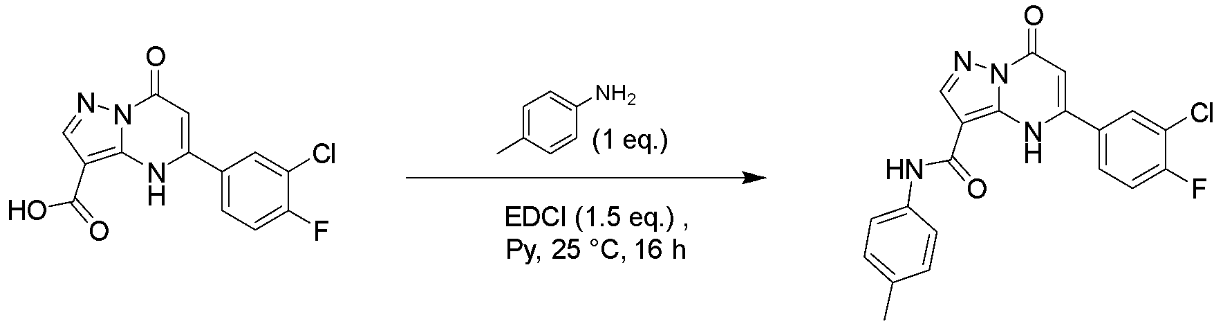

BRD5067-4 **BRD5067**

A mixture of 5-(3-chloro-4-fluoro-phenyl)-7-hydroxy-pyrazolo[1,5-a]pyrimidine-3-carboxylic acid **BRD5067-4** (500 mg, 1.63 mmol, 1 *eq.*), 4-methylaniline (174.14 mg, 1.63 mmol, 178.97 uL, 1 *eq.*) and EDCI (467.32 mg, 2.44 mmol, 1.5 *eq.*) in pyridine (10 mL) was degassed and purged with N_2 for 3 times, and then the mixture was stirred at 25 °C for 16 hours under N_2 atmosphere. After adding H_2O (15mL), the reaction mixture filtered under reduced pressure to give a filter cake. The residue was purified by prep-HPLC (column: Welch Xtimate C18 150×25mm×5um;mobile phase: [water(NH_3H_2O)-ACN];B%: 15%-45%,8min) to give 5-(3-chloro-4-fluoro-phenyl)-7-hydroxy-N-(p-tolyl)pyrazolo[1,5-a] pyrimidine-3-carboxamide **BRD5067** (77.57 mg, 193.65 umol, 11.92% yield, 99.06% purity) as a pink solid.

LC-MS (EC8679-45-P1S): t_R = 0.504 min, m/z: 397.0 $[M+H]^+$.

HPLC (EC8679-45-P1Z): t_R = 2.31 min, 99.06% purity (220 nm).

^{1}H NMR (400 MHz, DMSO-*d$_6$*) δ 10.80 (s, 1H), 8.32 (dd, *J*=7.2, 2.0 Hz, 1H), 8.11 - 8.04 (m, 2H), 7.63 -7.51 (m, 3H), 7.16 (d, *J*=8.4 Hz, 2H), 7.12-7.07 (m, 1H), 6.20 (s, 1H), 2.28 (s, 3H)

^{19}F NMR (377 MHz, DMSO-*d$_6$*) δ -116.328 (s, 1F)

^{13}C NMR (100 MHz, DMSO-d$_6$) δ = 161.57, 158.50, 157.02, 154.82, 152.98, 137.74, 131.77, 129.90, 129.10, 127.63, 120.39, 120.22, 118.74, 117.69, 117.48, 101.86, 92.57, 20.88

HRMS $C_{20}H_{14}ClFN_4O_2$; calculated $(M+H)^+$ = 397.0862; observed $(M+H)^+$ = 397.0861

HPLC purity: 98.881%

BRD5075

1.1 Procedure for preparation of BRD5075

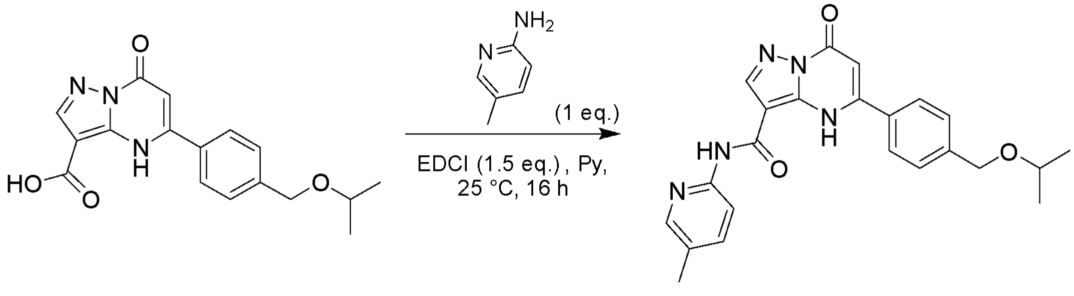

A mixture of 7-hydroxy-5-[4-(isopropoxymethyl)phenyl]pyrazolo[1,5-a]pyrimidine-3-carboxylic acid **BRD5928-6** (180 mg, 549.90 umol, 1 *eq.*), 5-methylpyridin-2-amine (59.47 mg, 549.90 umol, 1 *eq.*), EDCI (158.12 mg, 824.85 umol, 1.5 *eq.*), in Py (1 mL) was degassed and purged with N_2 for 3 times, and then the mixture was stirred at 25 °C for 16 hours under N_2 atmosphere. The reaction mixture was concentrated under reduced pressure to remove Py and the resulting residue was purified by prep-HPLC (column: Waters xbridge 150 × 25mm 10um;mobile phase: [water (ammonia hydroxide v/v)-ACN];B%: 15%-45%,9min), and then, the desired compound was purified by reversed-phase HPLC (0.1% NH_3•H_2O) to give 7-hydroxy-5-[4-(isopropoxymethyl)phenyl]-N-(5-methyl-2-pyridyl)pyrazolo[1,5-a]pyrimidine-3-carboxamide **BRD5075** (76.14 mg, 180.97 umol, 32.91% yield, 99.22% purity) as a yellow solid.

LC-MS (EC8679-45-P1S): t_R = 0.360 min, m/z: 418.2 $[M+H]^+$.

HPLC (EC8679-45-P1Z): t_R = 1.542 min, 99.22% purity (220 nm).

^{1}H NMR (400 MHz, DMSO-*d₆*) δ 11.56 (s, 1H), 8.26 - 8.12 (m, 3H), 7.64 (d, *J*=7.2 Hz, 1H), 7.54 - 7.36 (m, 2H), 7.20 (s, 1H), 7.12 (s, 1H), 6.96 (s, 1H), 6.29 - 6.19 (m, 1H), 4.56 (s, 2H), 3.75 - 3.62 (m, 1H), 2.28 (s, 3H), 1.19 (d, *J*=6.1 Hz, 6H)

^{13}C NMR (101 MHz, DMSO-d_6) δ 161.27, 158.50, 156.62, 150.37, 147.93, 142.31, 140.81, 138.50, 137.27, 129.71, 129.30, 127.48, 126.72, 112.81, 100.53, 92.08, 70.50, 68.64, 22.09, 17.31.

HRMS $C_{23}H_{23}N_5O_3$; calculated $(M+H)^+$ = 418.1874; observed $(M+H)^+$ = 418.1873

HPLC purity: 99.113%

BRD5078

1.1 Procedure for preparation of BRD5078

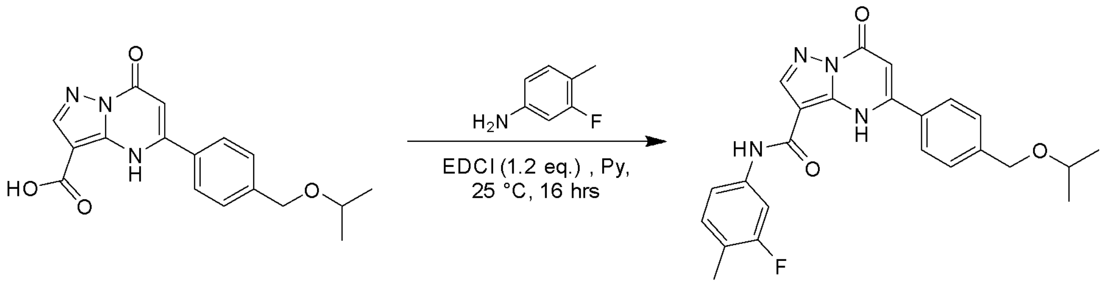

BRD5928-6 **BRD5078**

To a solution of 7-hydroxy-5-[4-(isopropoxymethyl)phenyl]pyrazolo[1,5-a]pyrimidine-3-carboxylic acid **BRD5928-6** (200 mg, 611.00 umol, 1.0 *eq.*) and 3-fluoro-4-methyl-aniline (114.69 mg, 916.50 umol, 1.5 *eq.*) in Py (3.0 mL) was added EDCI (175.69 mg, 916.50 umol, 1.5 *eq.*). The mixture was stirred at 25 °C for 16 hours. The reaction mixture was quenched by addition **H_2O 30** mL at **25** °C, and then diluted with **EA 30.0** mL and extracted with **EA 150** mL (**50** mL × **3).** The combined organic layers were washed with **brine 100** mL (**50** mL × **2**), dried over **Na_2SO_4**, filtered and concentrated under reduced pressure to give a residue. The residue was purified by prep-HPLC (column: Phenomenex luna C_{18} 150×25mm×10um; mobile phase: [water(FA)-ACN]; B%:55%-85%,10min) to give N-(3-fluoro-4-methyl-phenyl)-7-hydroxy-5- [4-(isopropoxymethyl)phenyl]pyrazolo[1,5-a]pyrimidine-3-carboxamide **BRD5078** (66.12 mg, 150.67 umol, 24.66% yield, 99% purity) as an off-white solid.

LCMS: t_R = 0.505 min, m/z: 435.2 $[M+H]^+$.

^{1}H NMR (400 MHz, DMSO-*d_6*) δ 11.15 (s, 1H), 10.12 (s, 1H), 8.54 (s, 1H), 7.82 (d, *J* = 7.2 Hz, 2H), 7.65 (d, *J* = 12.4 Hz, 1H), 7.54 (d, *J* = 7.6 Hz, 2H), 7.39 (d, *J* = 8.0 Hz, 1H), 7.24 (t, *J* = 8.4 Hz, 1H), 6.26 (s, 1H), 4.57 (s, 2H), 3.75 - 3.69 (m, 1H), 2.22 (s, 3H), 1.19 (d, *J* = 6.1 Hz, 6H).

^{19}F NMR (377 MHz, DMSO-*d_6*) δ -116.258 (s, 1F)

^{13}C NMR (100 MHz, DMSO-d_6) δ = 162.01, 161.49, 159.61, 156.07, 143.59, 141.67, 138.63, 138.52, 131.77, 131.71, 128.25, 127.30, 119.45, 119.29, 116.16, 107.54, 107.28, 100.80, 96.99, 71.23, 69.03, 22.492, 13.92

HRMS $C_{24}H_{23}FN_4O_3$; calculated $(M+H)^+$ = 435.1827; observed $(M+H)^+$ = 435.1828

HPLC purity: 98.508%

BRD5079

1.1 Procedure for preparation of BRD5079

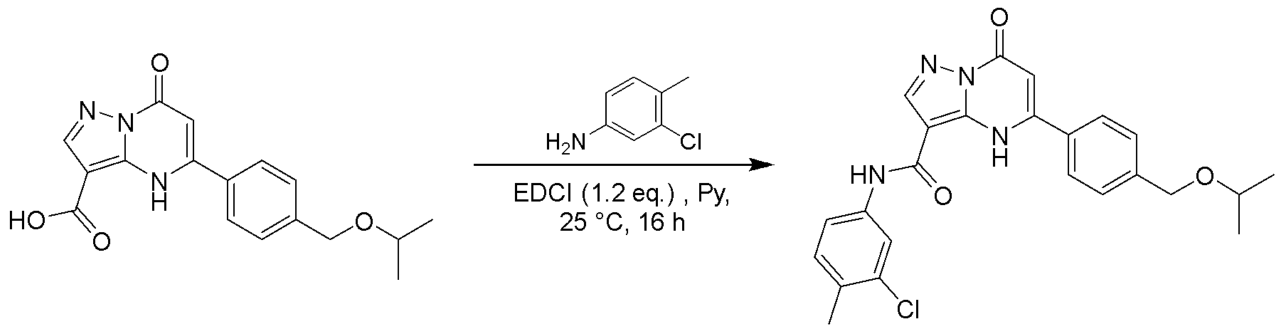

BRD5928-6

BRD5079

To a solution of 3-chloro-4-methyl-aniline **BRD5928-6** (93.44 mg, 659.88 umol, 80.55 uL, 1.2 *eq.*) and 7-hydroxy-5-[4-(isopropoxymethyl) phenyl]pyrazolo[1,5-a]pyrimidine-3-carboxylic acid (180 mg, 549.90 umol, 1.0 *eq.*) in Py (3 mL), EDCI (158.12 mg, 824.85 umol, 1.5 *eq.*) was added. The mixture was stirred at 25 °C for 16 hours. The reaction mixture was filtered and concentrated under reduced, and the resulting residue was purified by prep-HPLC (column: Welch Xtimate C18 150×25mm×5um; mobile phase: [water (NH_3•H_2O)-ACN]; B%: 20%-50%, 8min) to give N-(3-chloro-4-methyl-phenyl)-7-hydroxy-5-[4-(isopropoxymethyl)phenyl]pyrazolo[1,5-a]pyrimidine-3-carboxamide **BRD5079** (164.91 mg, 351.39 umol, 63.90% yield, 96.08% purity) as a pink solid.

LCMS: t_R = 0.585 min, m/z: 451.4 $[M+H]^+$.

^{1}H NMR (400 MHz, DMSO-d_6) δ 11.19 (s, 1H), 8.08 - 8.03 (m, 3H), 7.52 - 7.38 (m, 2H), 7.35 (s, 1H), 7.26 - 7.05 (m, 2H), 6.94 (s, 1H), 6.15 (s, 1H), 4.55 (s, 2H), 3.72 - 3.65 (m, 1H), 2.30 (s, 3H), 1.18 (d, J = 6.0 Hz, 6H)

^{13}C NMR (101 MHz, DMSO) δ 161.35, 144.99, 142.31, 138.94, 133.27, 132.28, 131.51, 128.82, 128.00, 127.43, 126.43, 125.27, 123.45, 118.44, 116.90, 100.75, 92.32, 70.46, 68.71, 22.09, 18.93.

HRMS $C_{24}H_{23}ClN_4O_3$; calculated $(M+H)^+$ = 451.1531; observed $(M+H)^+$ = 451.1529

HPLC purity: 96.088%

BRD5080

1.1 Procedure for preparation of BRD5080

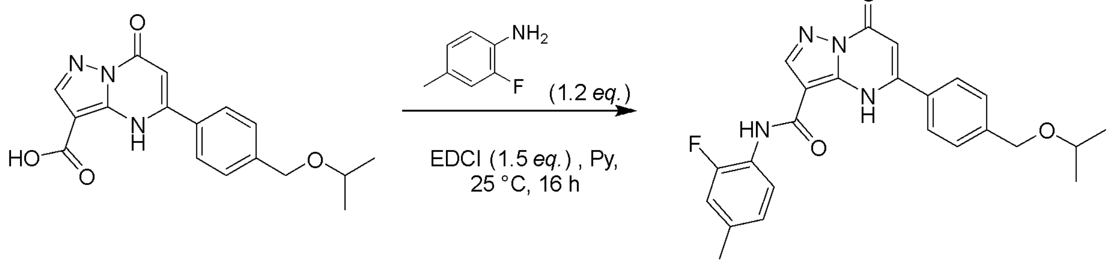

BRD5928-6

BRD5080

To a mixture of 7-hydroxy-5-[4-(isopropoxymethyl)phenyl]pyrazolo[1,5-a]pyrimidine-3-carboxylic acid **BRD5928-6** (180 mg, 549.90 umol, 1.0 *eq.*), 2-fluoro-4-methyl-aniline (82.58 mg, 659.88 umol, 74.40 uL, 1.2 *eq.*) and in Py (2 mL), EDCI (158.12 mg, 824.85 umol, 1.5 *eq.*) was added. The mixture was stirred at 25 °C for 16 hours. The reaction mixture was concentrated under reduced pressure to give a residue. The residue was purified prep-HPLC (column: Waters X bridge BEH C^{18}100 × 30 mm × 10 um; mobile phase: [water (ammonia hydroxide v/v) - ACN]; B%: 21% - 51%, 2 min) to give *N*-(2-fluoro-4-methyl-phenyl)-7-hydroxy-5-[4-(isopropoxymethyl) phenyl]pyrazolo[1,5-*a*]pyrimidine-3-carboxamide **BRD5080** (72.82 mg, 165.60 umol, 30.11% yield, 98.80% purity) as a white solid.

LCMS: t_R = 0.516 min, m/z: 435.0 $[M+H]^+$.

^{1}H NMR (400 MHz, DMSO-d_6) δ 8.46 (s, 1H), 8.09 (s, 2H), 7.52 - 7.42 (m, 2H), 7.22 - 7.09 (m, 3H), 7.01 - 6.97 (m, 2H), 6.16 (s, 1H), 4.55 (s, 2H), 3.72 - 3.66 (m, 1H), 2.31 (s, 3H), 1.18 (d, *J* = 6.4 Hz, 6H).

^{13}C NMR (101 MHz, DMSO-d_6) δ 161.69, 158.44, 157.59, 150.85, 142.91, 140.76, 138.17, 132.65, 127.71, 127.14, 125.86, 125.34, 125.31, 120.97, 115.97, 101.49, 92.68, 70.95, 69.14, 22.55, 20.80.

^{19}F NMR (377 MHz, DMSO-d_6) δ -130.602 (s, 1F)

HRMS $C_{24}H_{23}FN_4O_3$; calculated $(M+H)^+$ = 435.1827; observed $(M+H)^+$ = 435.1829

HPLC purity: 99.304%

NMR Spectra

^{1}**H NMR** (400 MHz, DMSO-d_6) of **BRD2813**

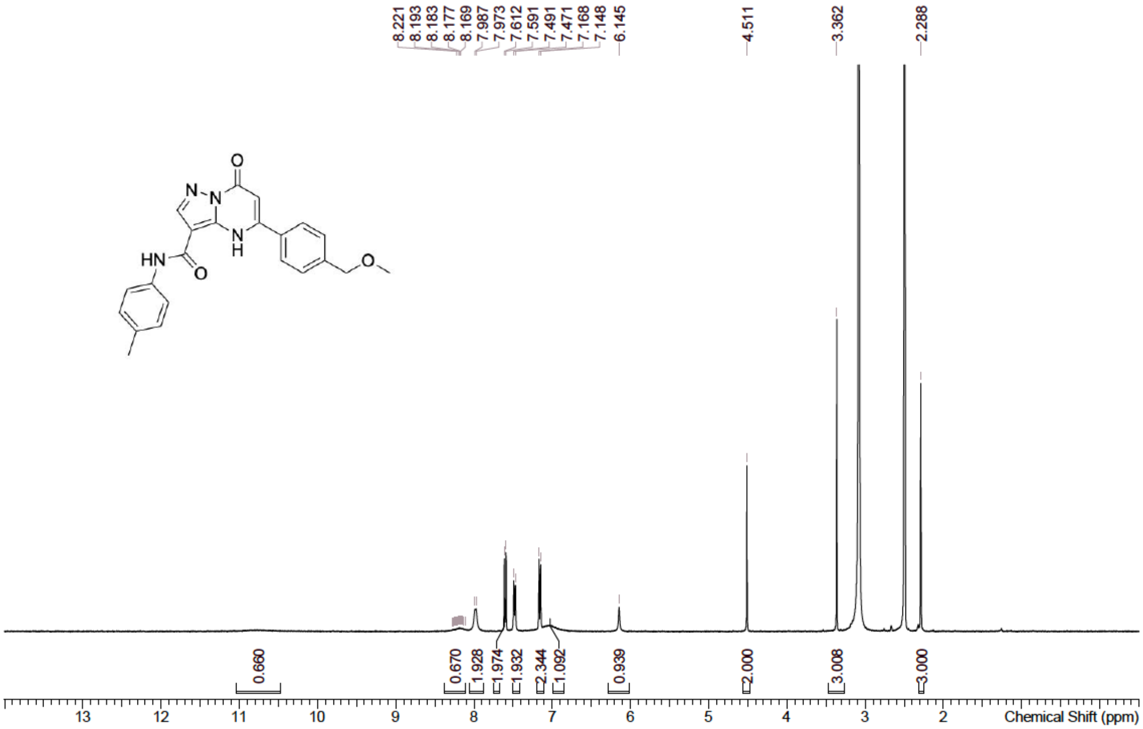

^{13}**C NMR** (101 MHz, DMSO-d_6) of **BRD2813**

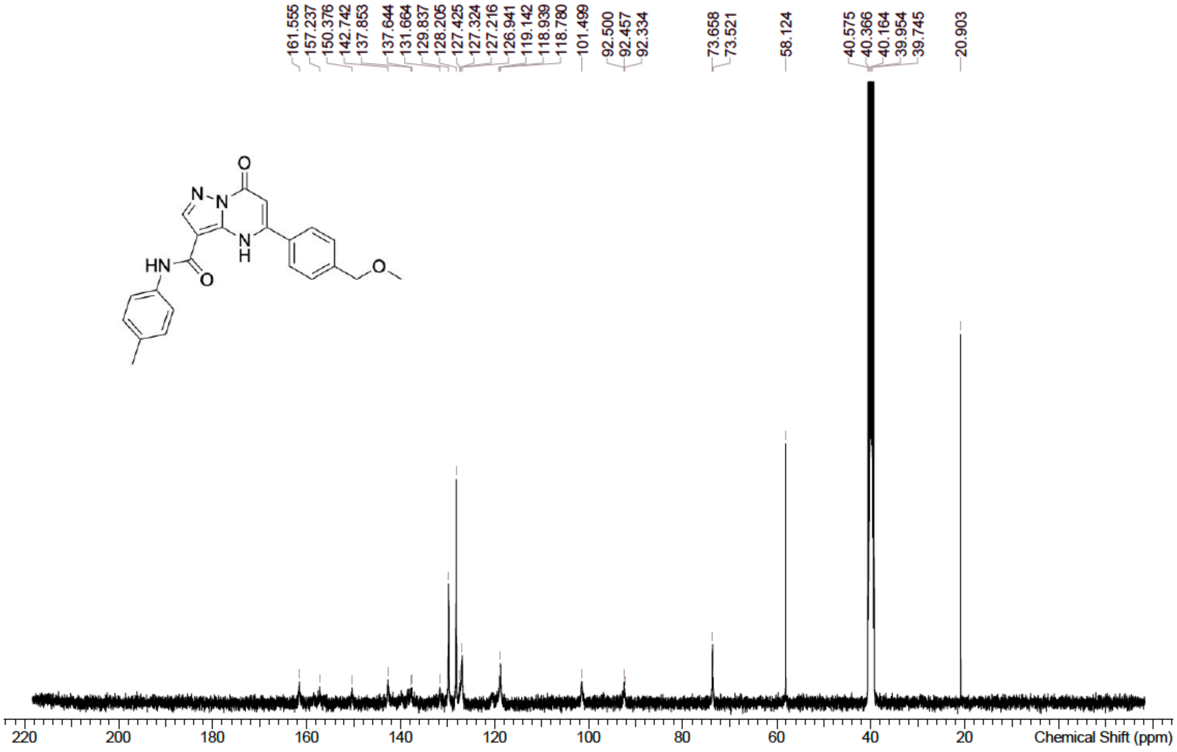

^{1}H NMR (400 MHz, DMSO-*d*6) of **BRD2166**

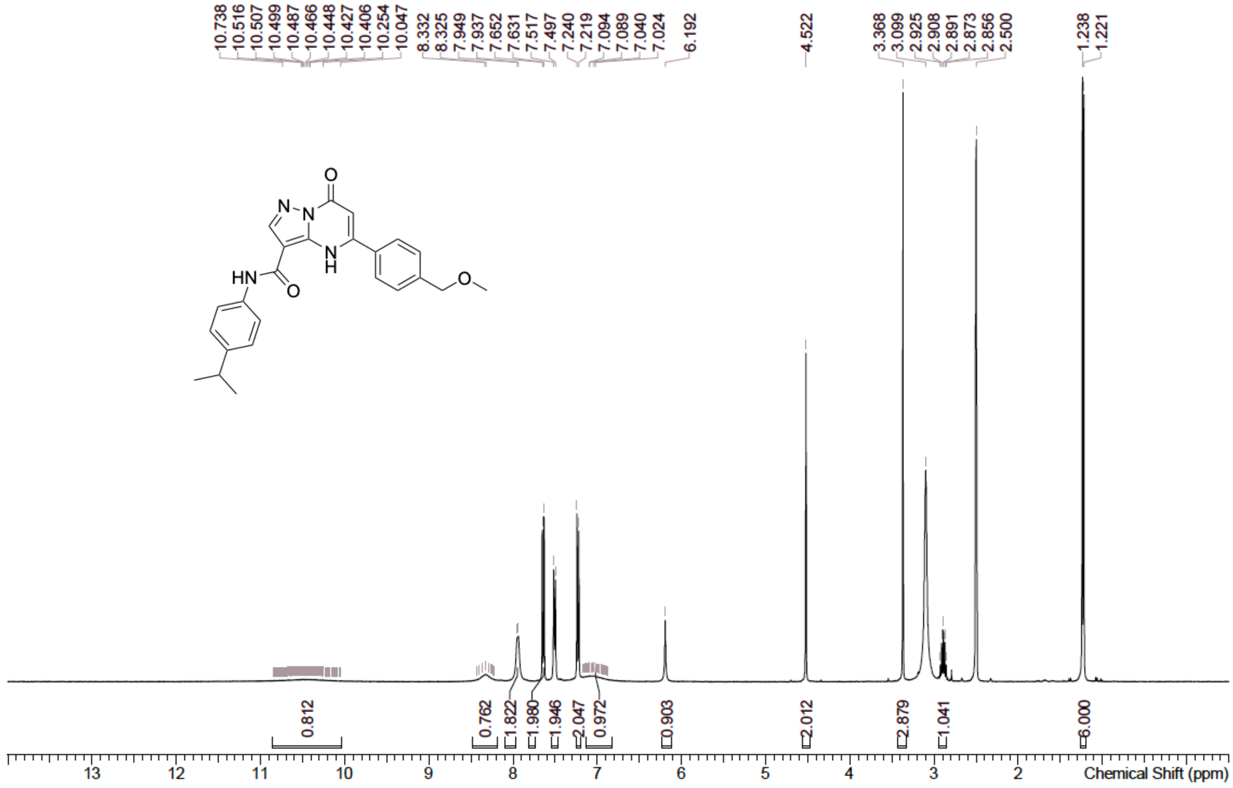

^{13}C NMR (101 MHz, DMSO-*d*6) of **BRD2166**

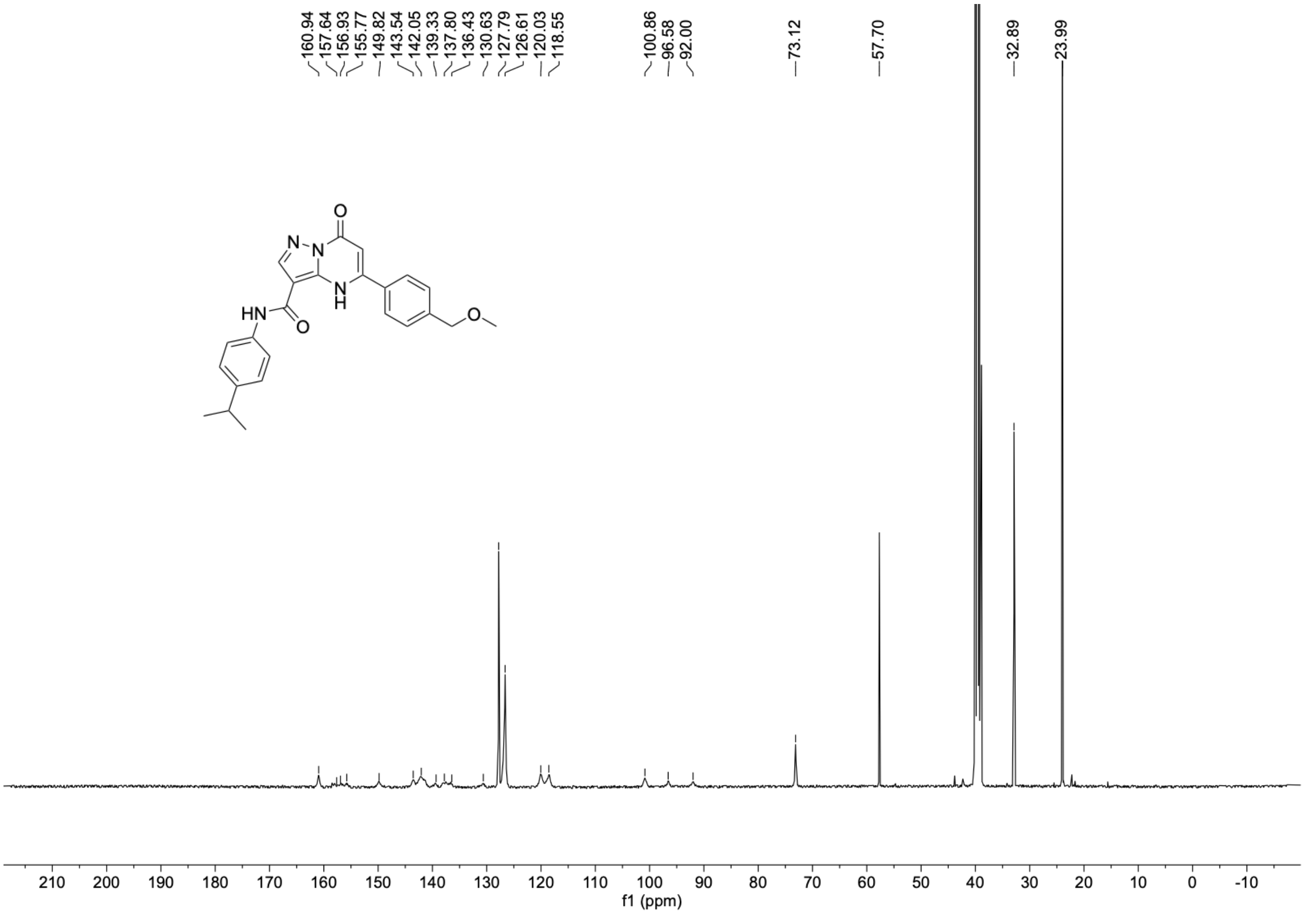

[1]**H NMR** (400 MHz, DMSO-d_6) of **BRD5928**

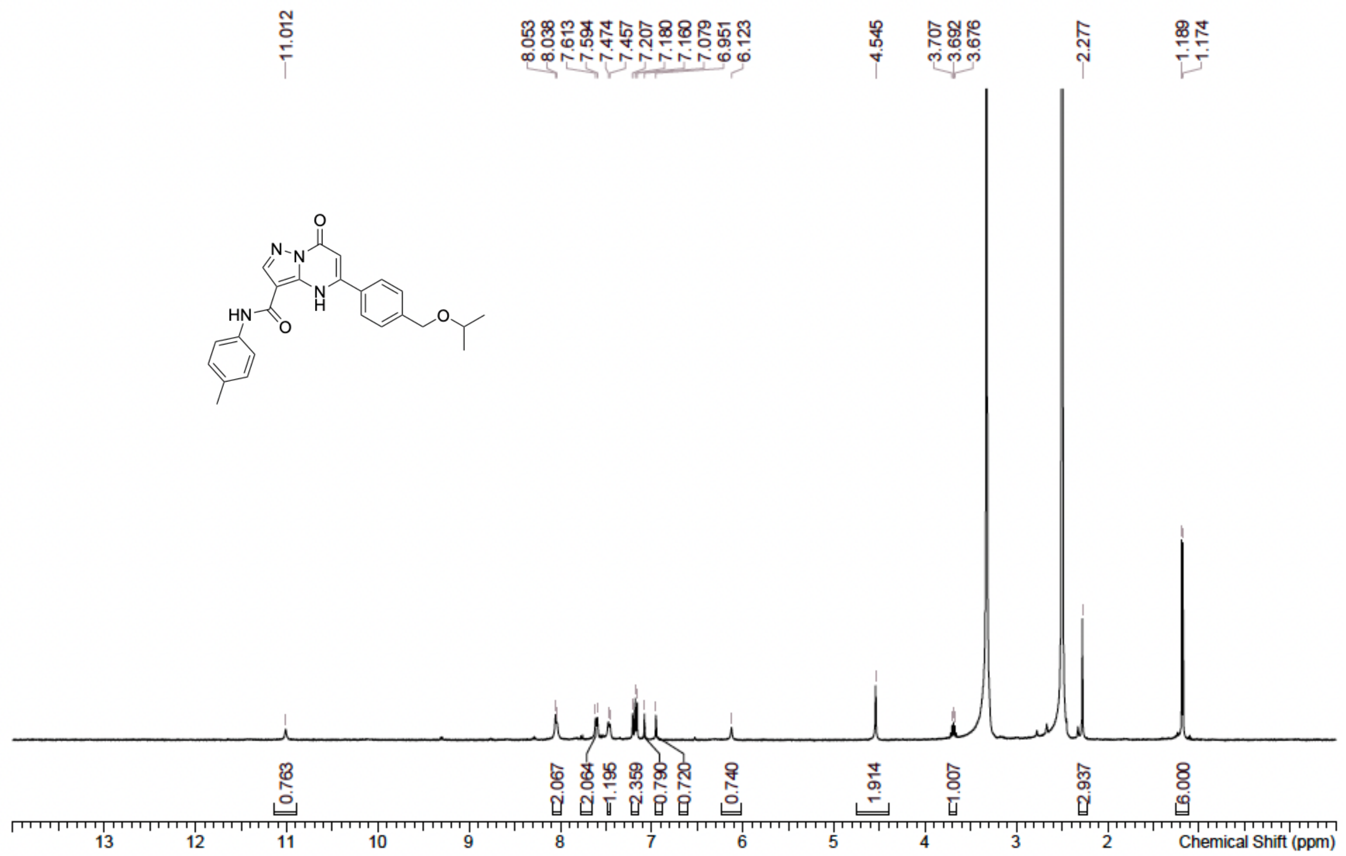

^{13}C NMR (101 MHz, DMSO-d_6) of **BRD5928**

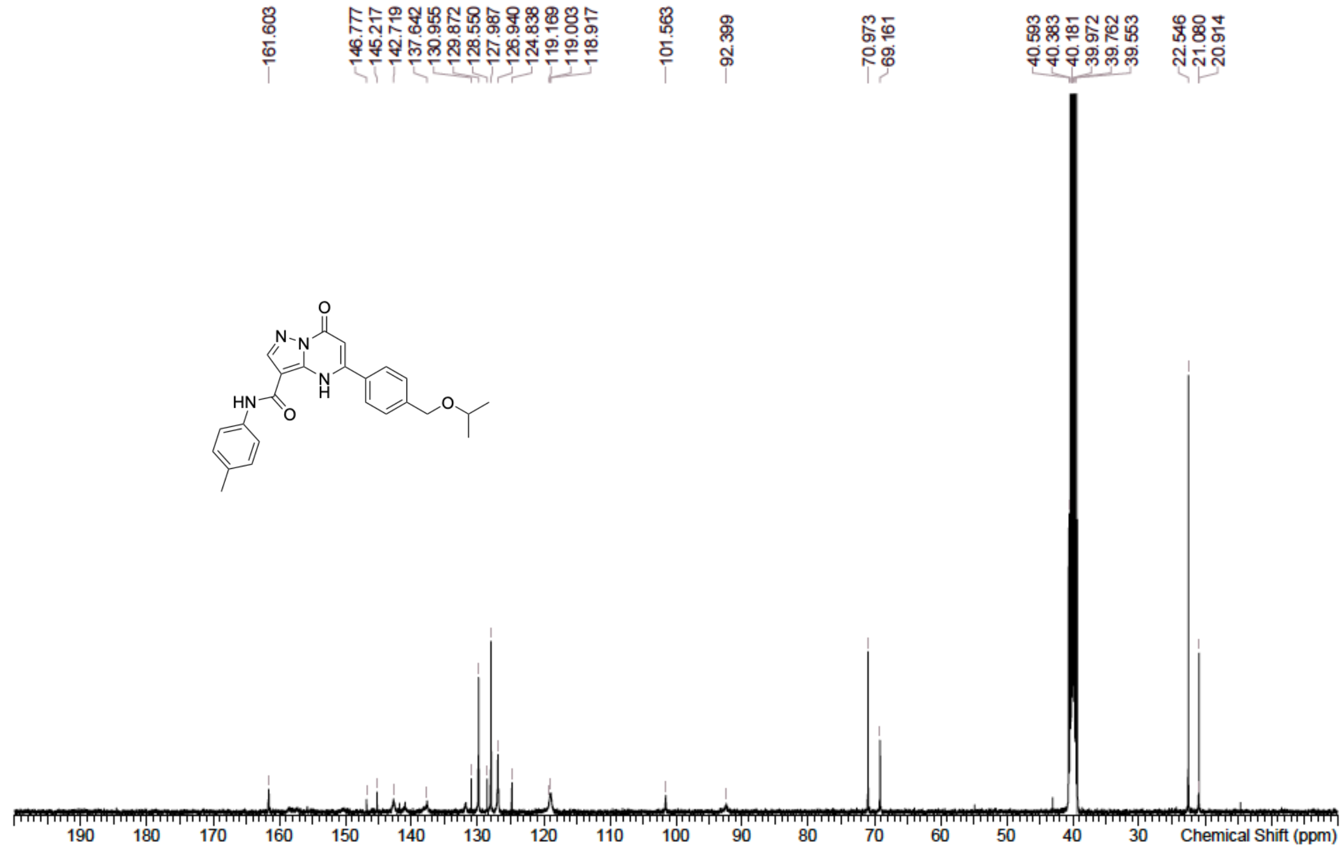

^{1}H NMR (400 MHz, DMSO-d_6) of **BRD5059**

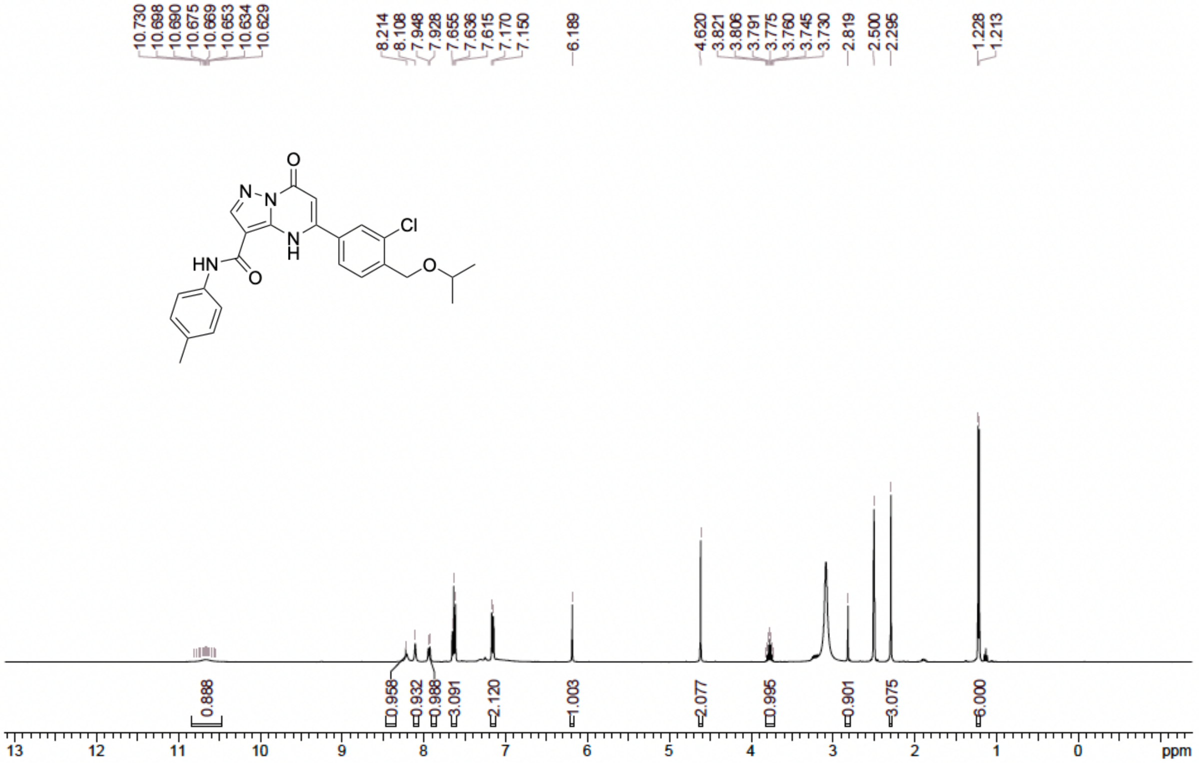

^{13}C NMR (101 MHz, DMSO-*d*$_6$) of **BRD5059**

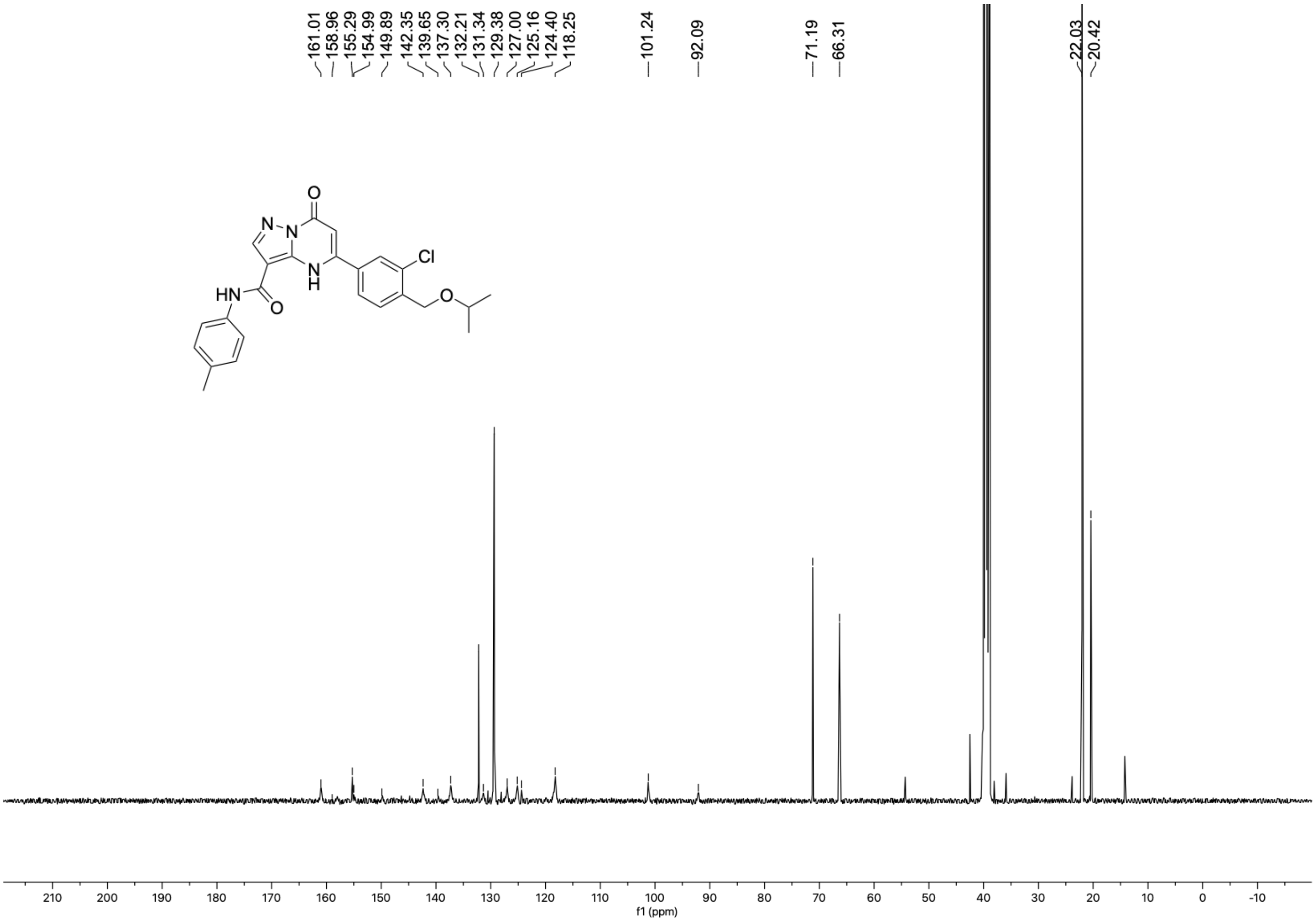

^{1}H NMR (400 MHz, DMSO-*d*$_6$) of **BRD5067**

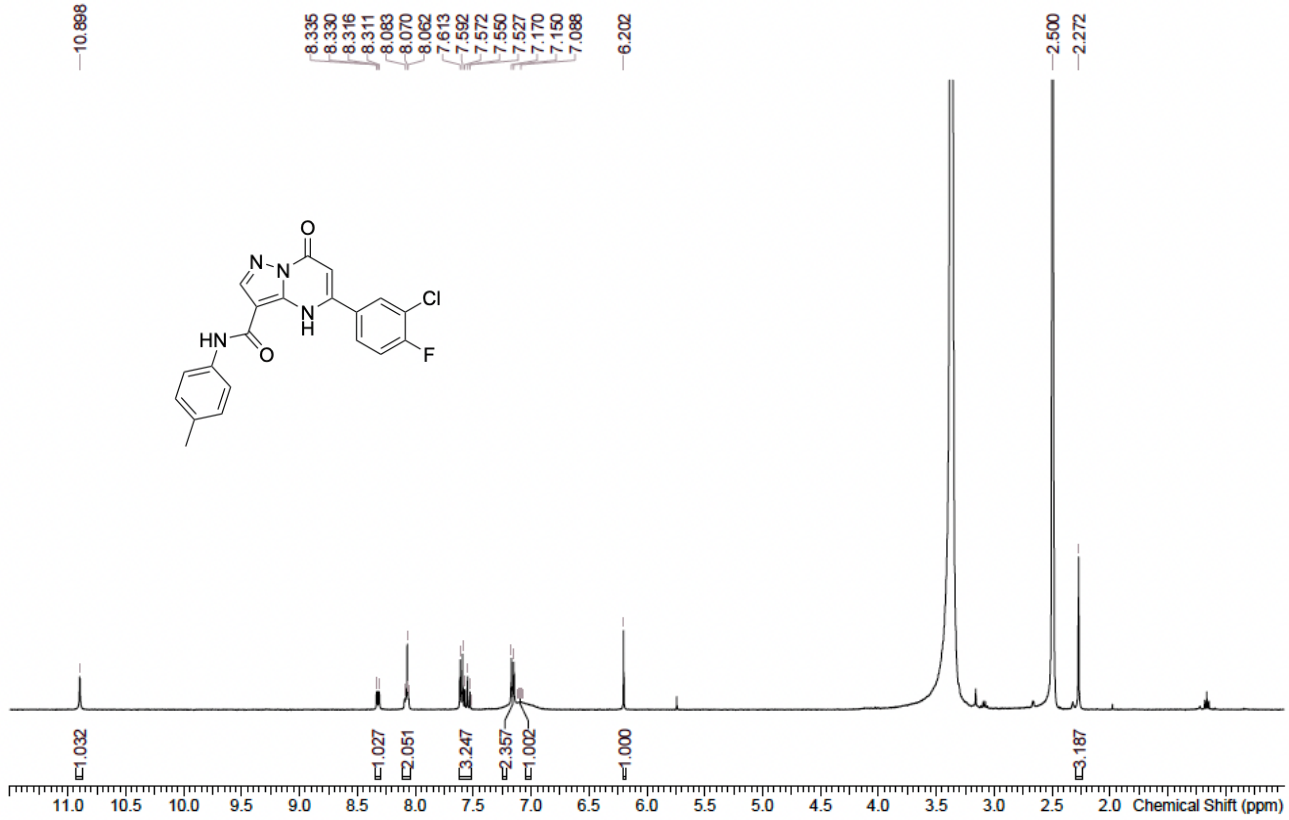

^{19}F NMR (377 MHz, DMSO-d_6) of **BRD5067**

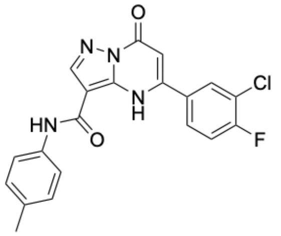

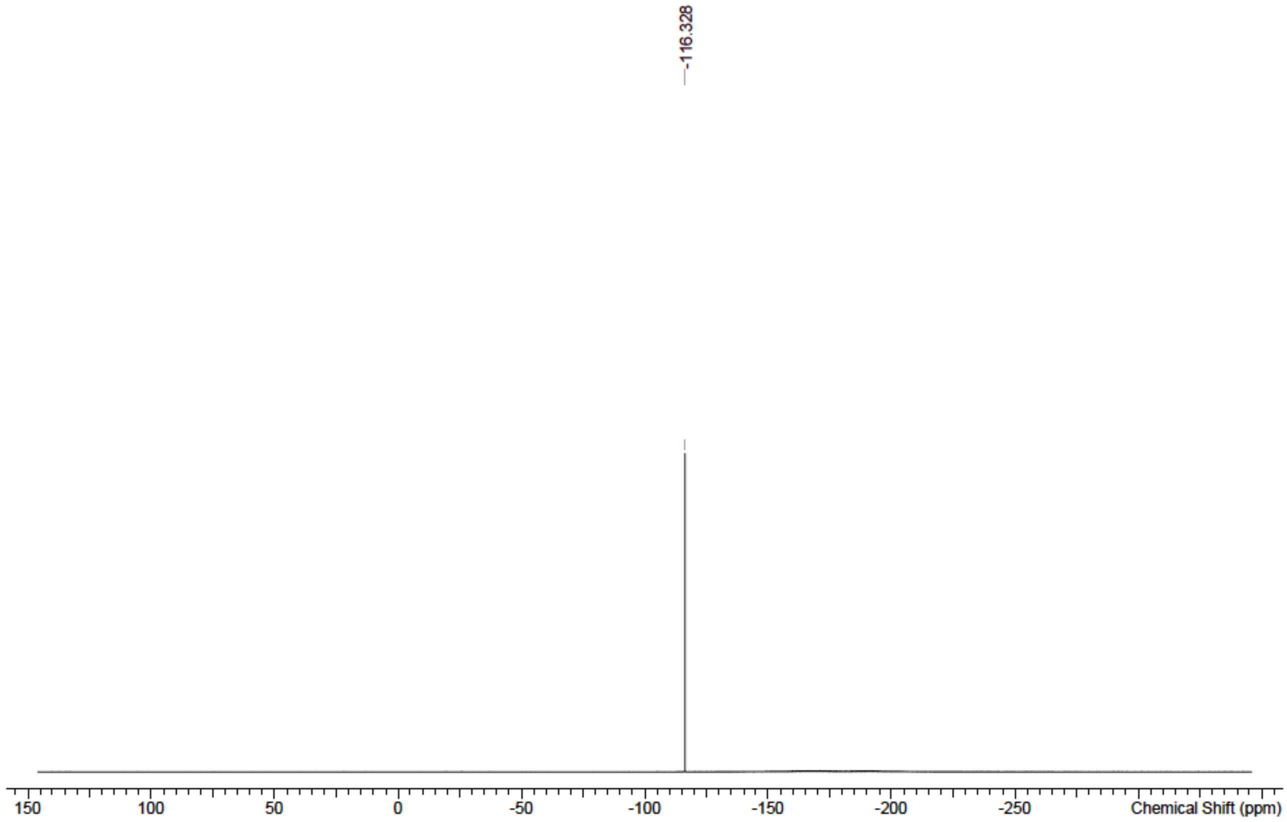

^{13}C NMR (101 MHz, DMSO-d_6) of **BRD5067**

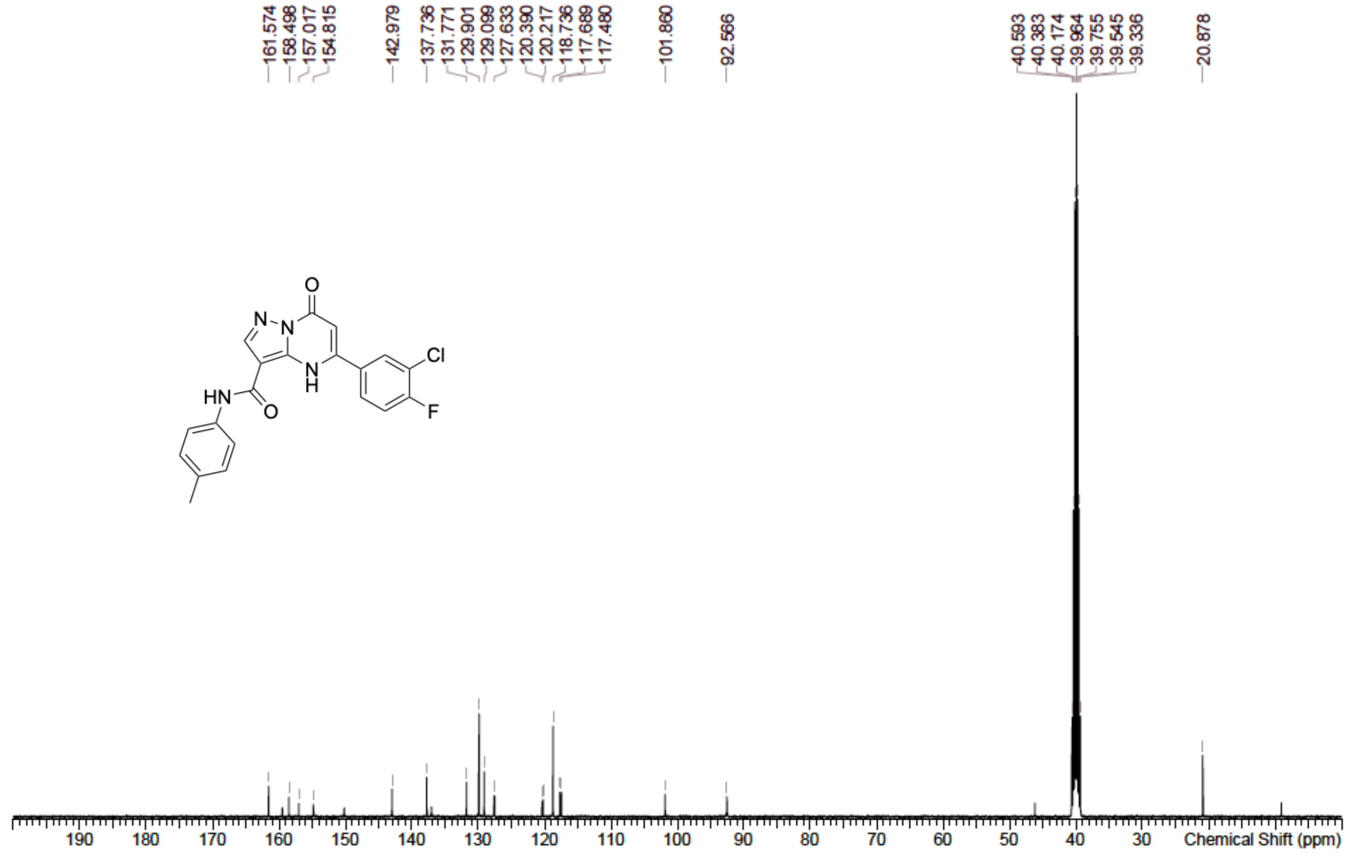

^{1}H NMR (400 MHz, DMSO-d_6) of **BRD5075**

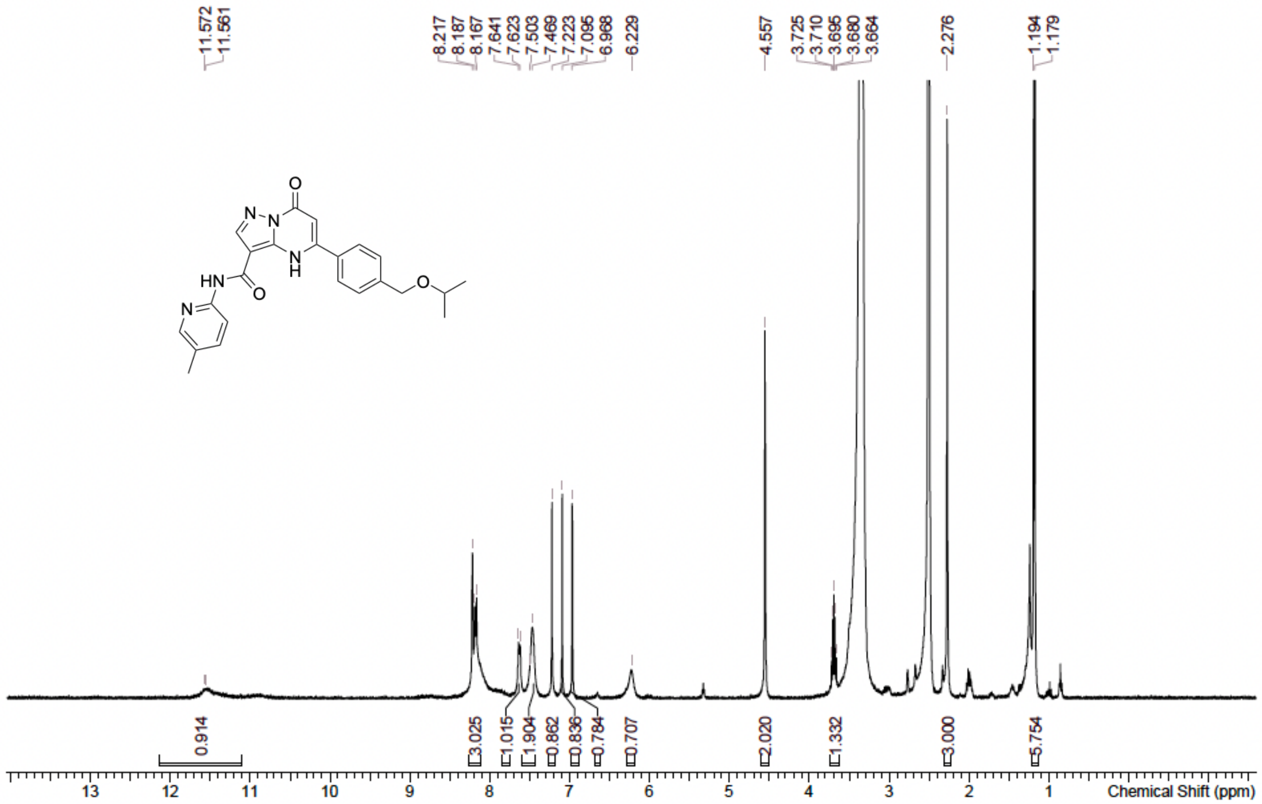

^{13}C NMR (101 MHz, DMSO-d_6) of **BRD5075**

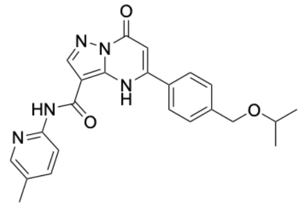

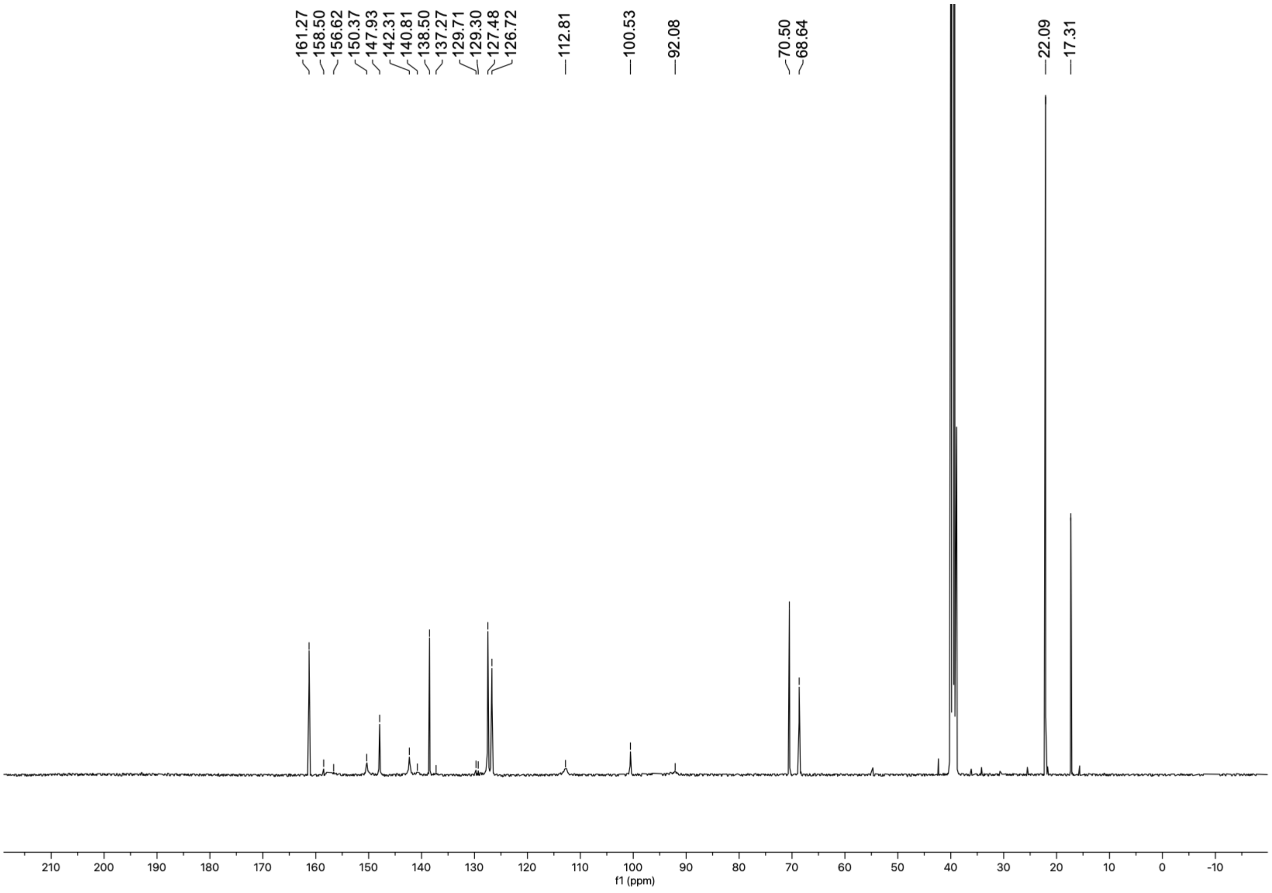

^{1}H NMR (400 MHz, DMSO-d_6) of **BRD5078**

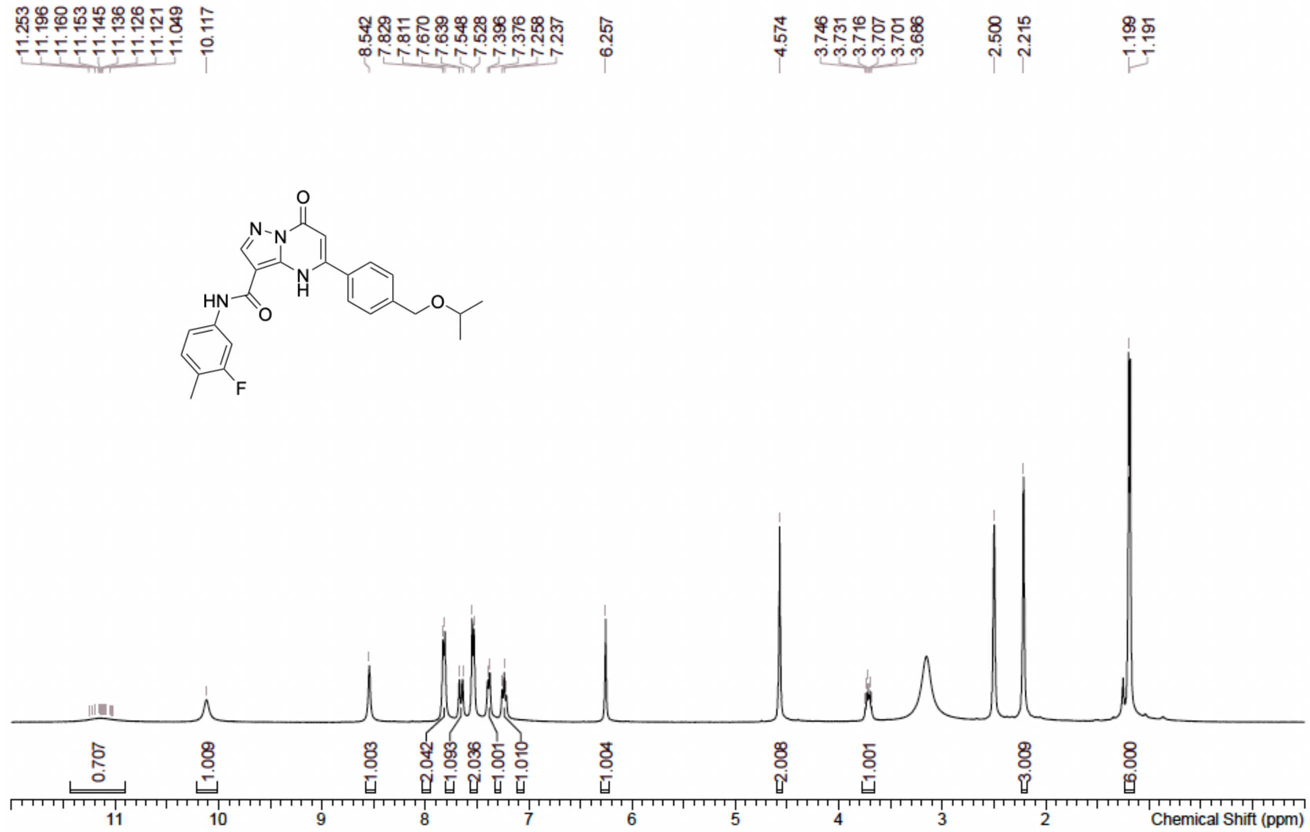

^{19}F NMR (377 MHz, DMSO-d_6) of **BRD5078**

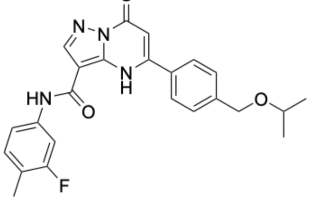

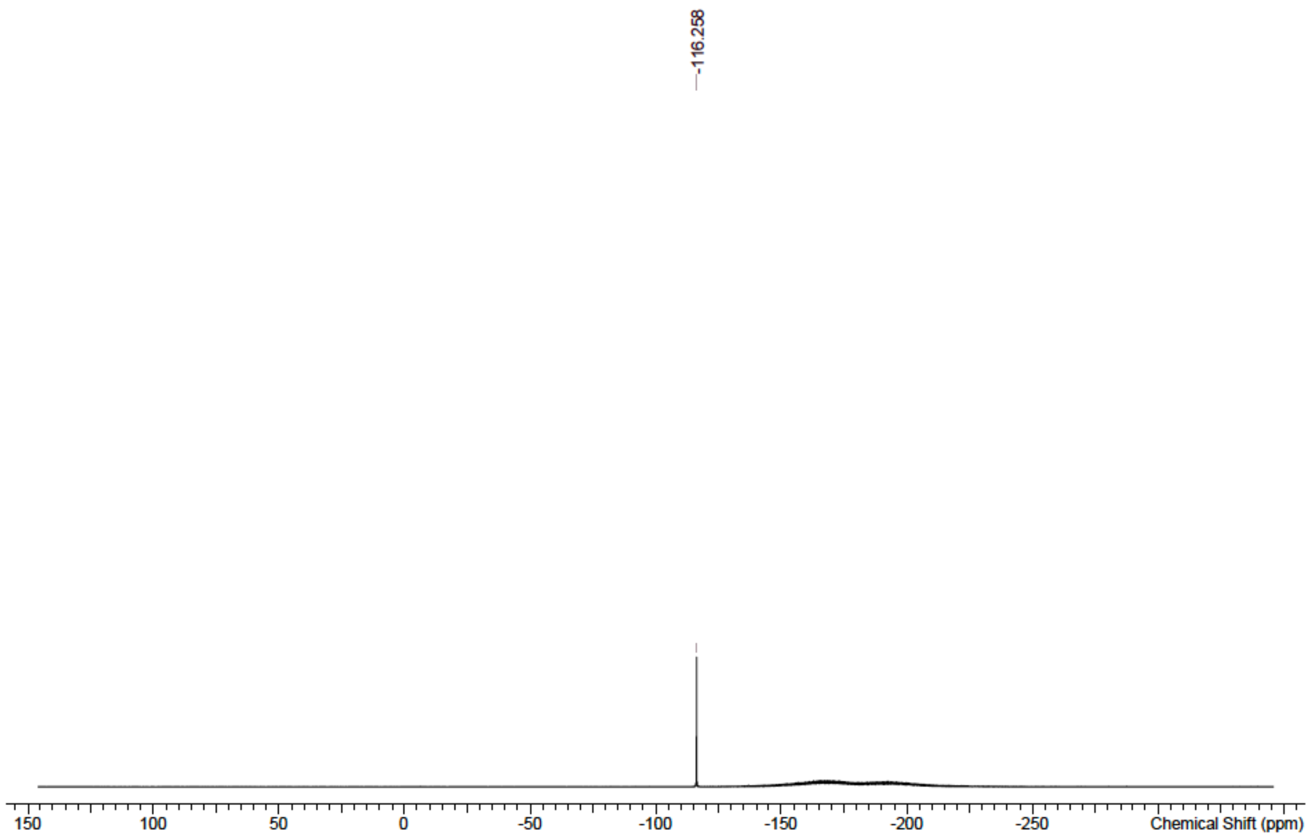

^{13}C NMR (101 MHz, DMSO-d_6) of **BRD5078**

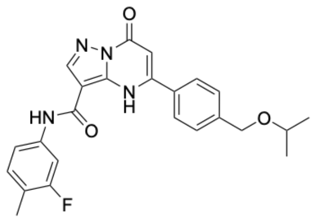

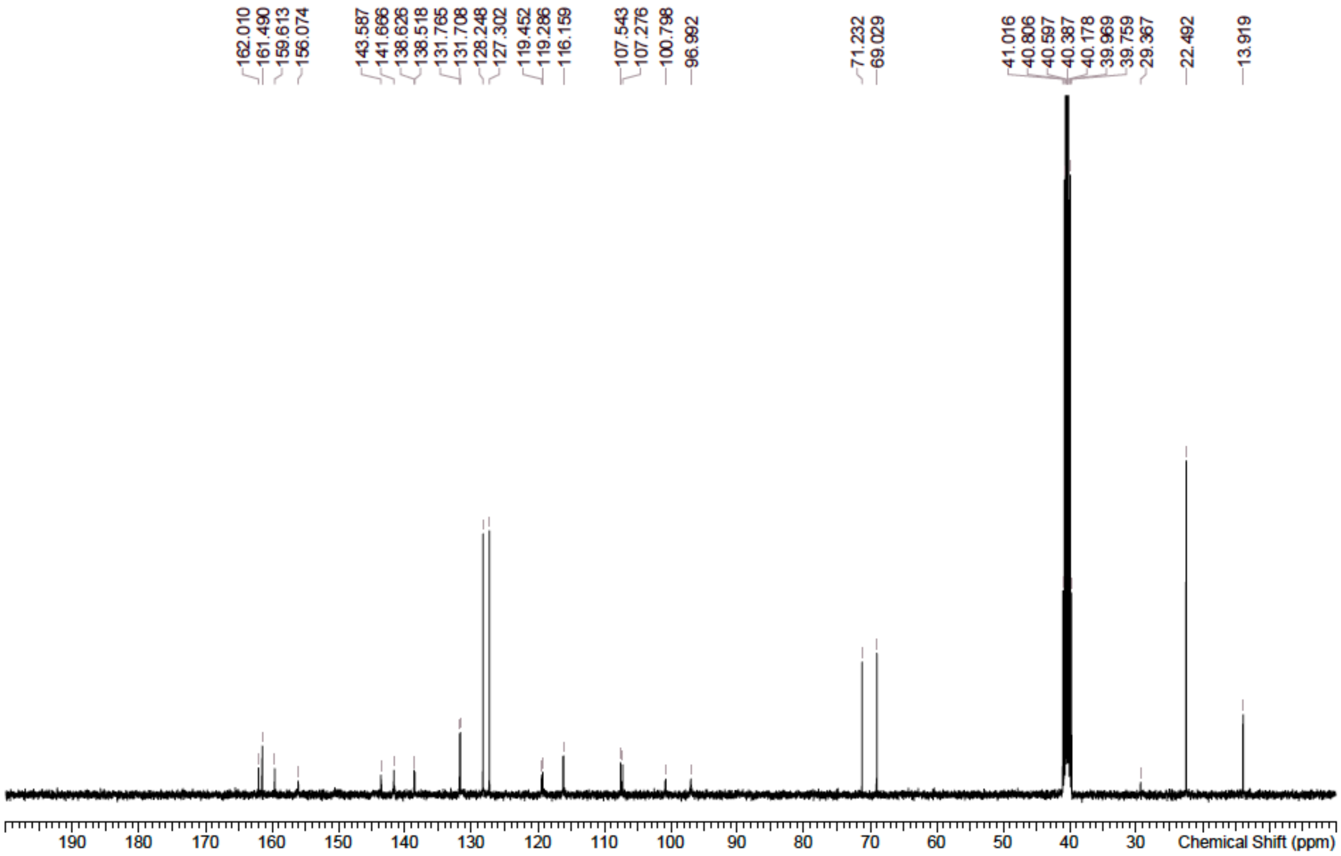
162.010
161.490
159.613
156.074
143.587
141.666
138.626
138.518
131.765
131.708
128.248
127.302
119.452
119.286
116.159
107.543
107.276
100.798
96.992
71.232
69.029
41.016
40.806
40.597
40.387
40.178
39.969
39.759
29.367
22.492
13.919
190
180
170
160
150
140
130
120
110
100
90
80
70
60
50
40
30
Chemical Shift (ppm)

^{1}H NMR (400 MHz, DMSO-d_6) of **BRD5079**

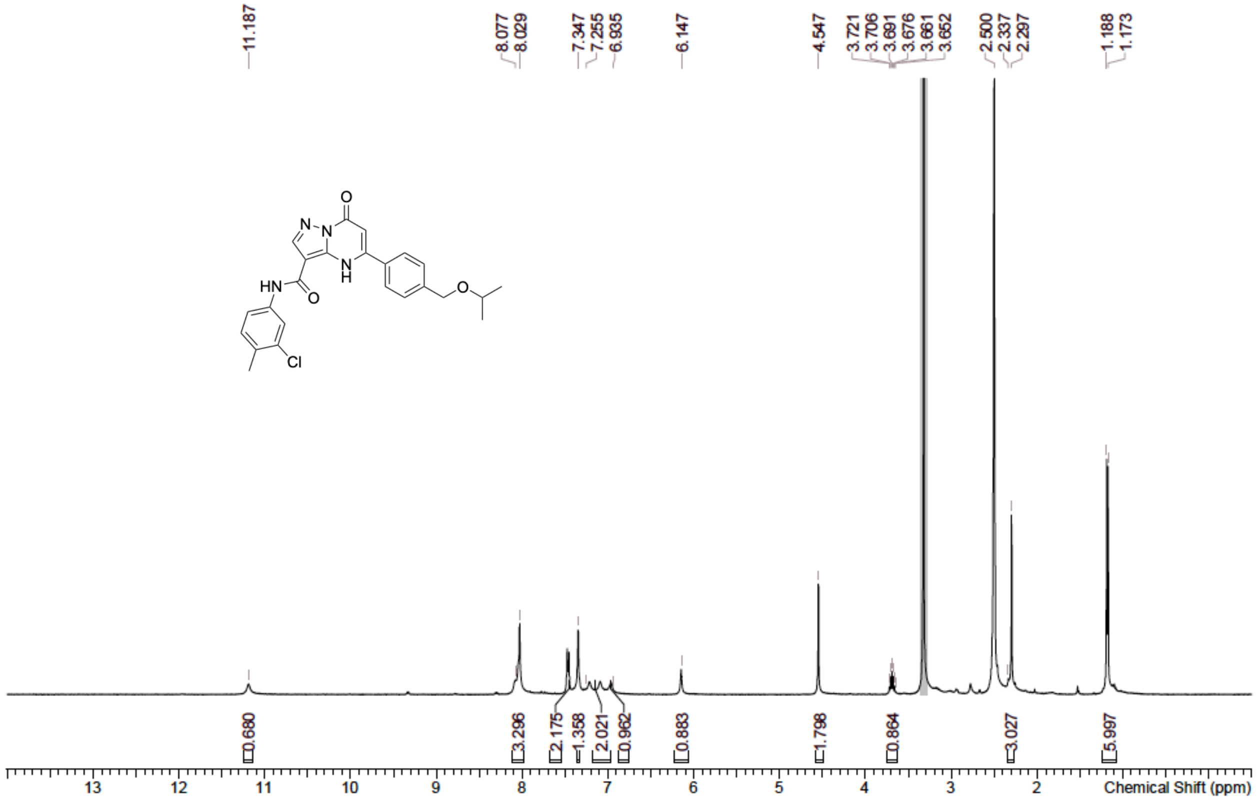

^{13}C NMR (101 MHz, DMSO-d_6) of **BRD5079**

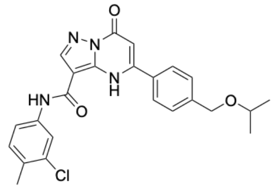

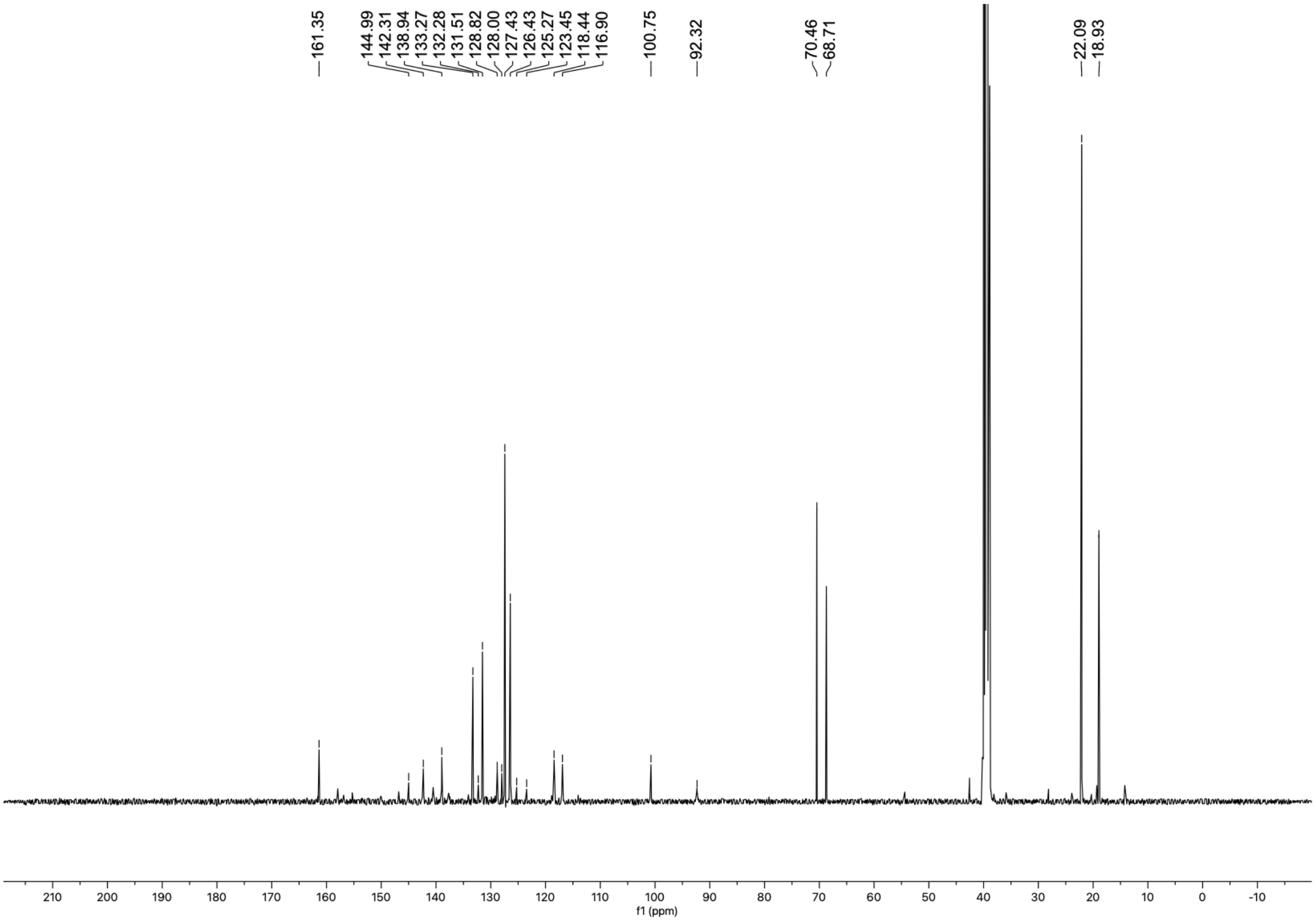

^{1}H NMR (400 MHz, DMSO-d_6) of **BRD5080**

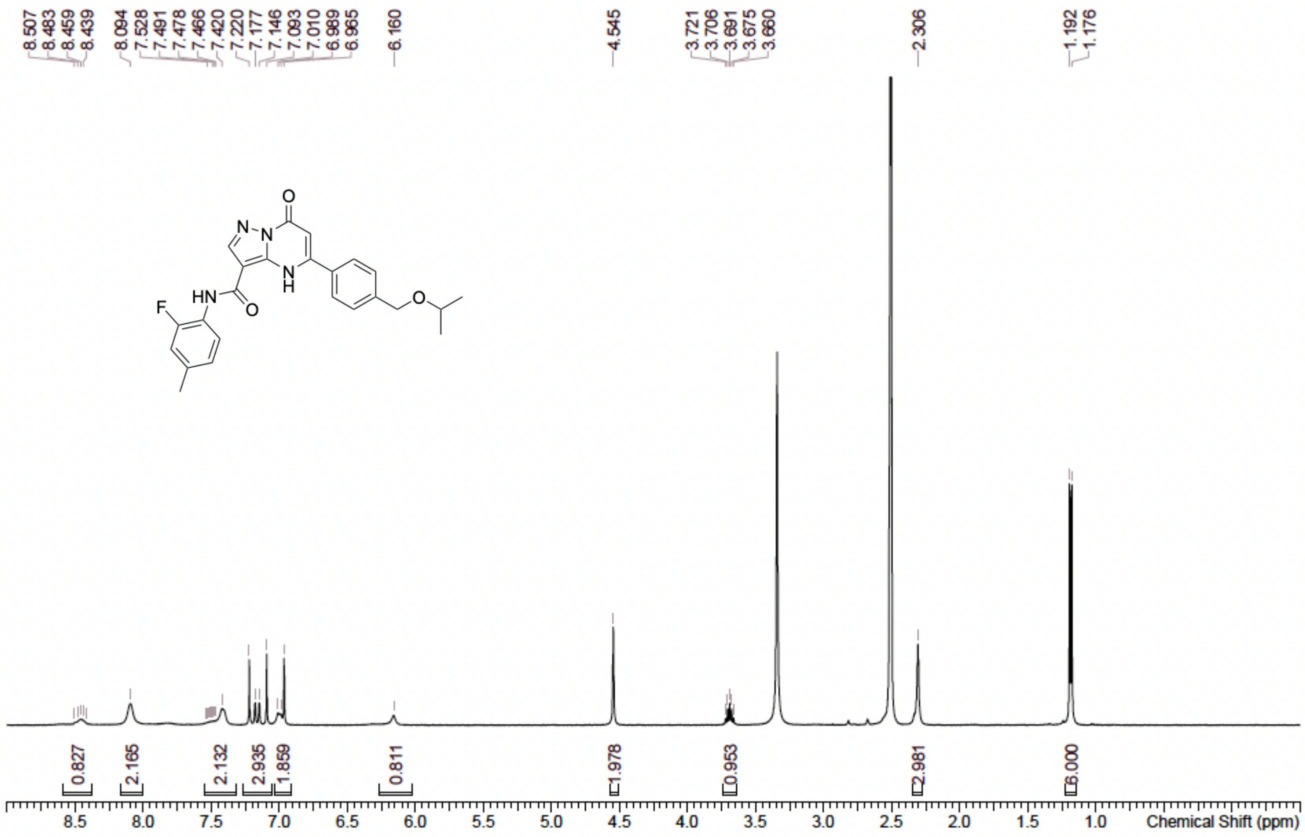

^{19}F NMR (377 MHz, DMSO-d_6) of **BRD5080**

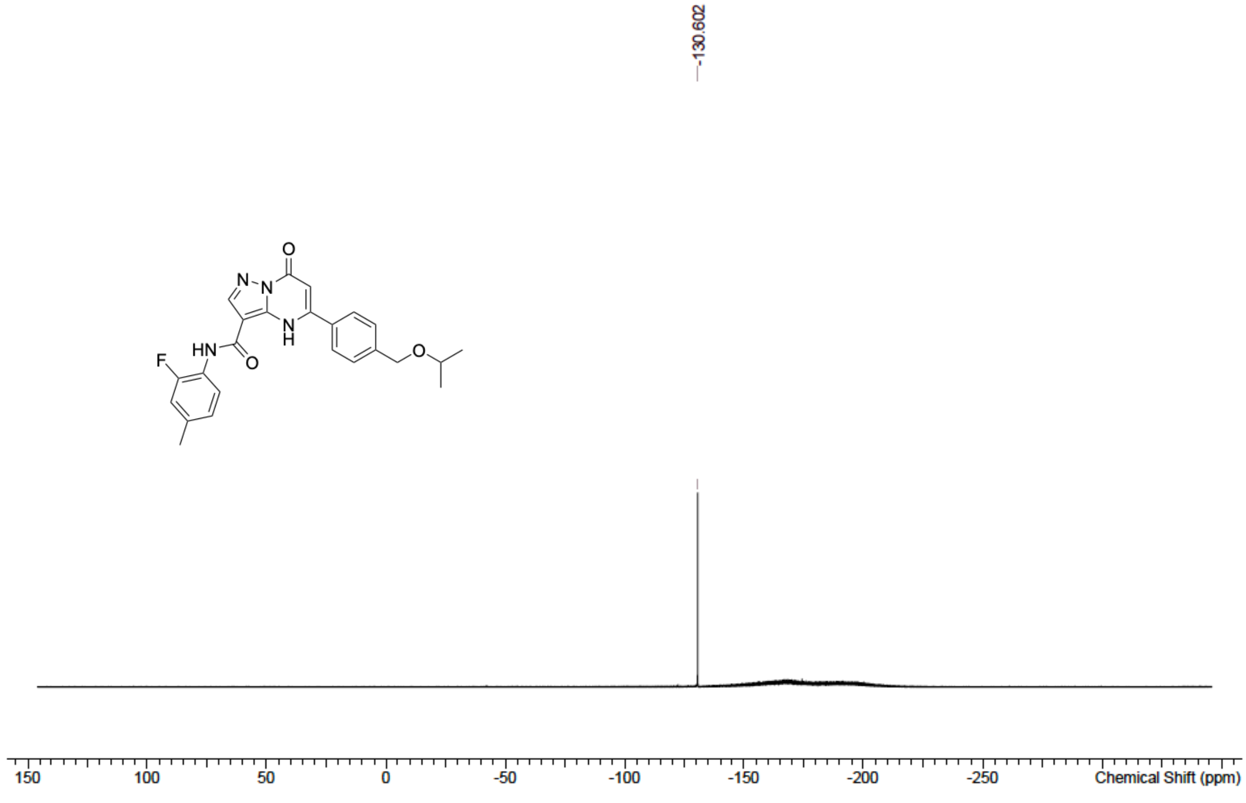

^{13}C NMR (101 MHz, DMSO-d_6) of **BRD5080**

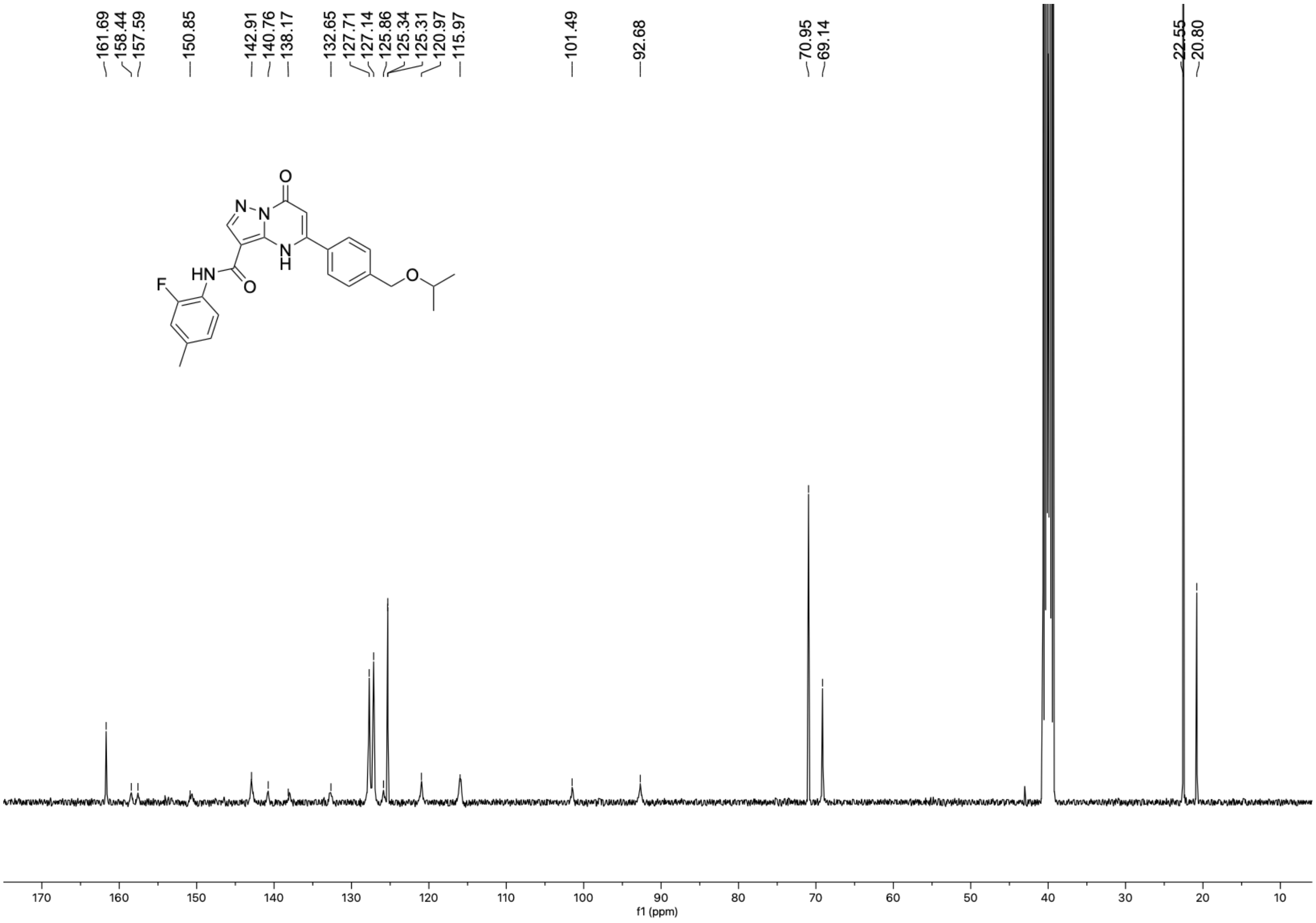

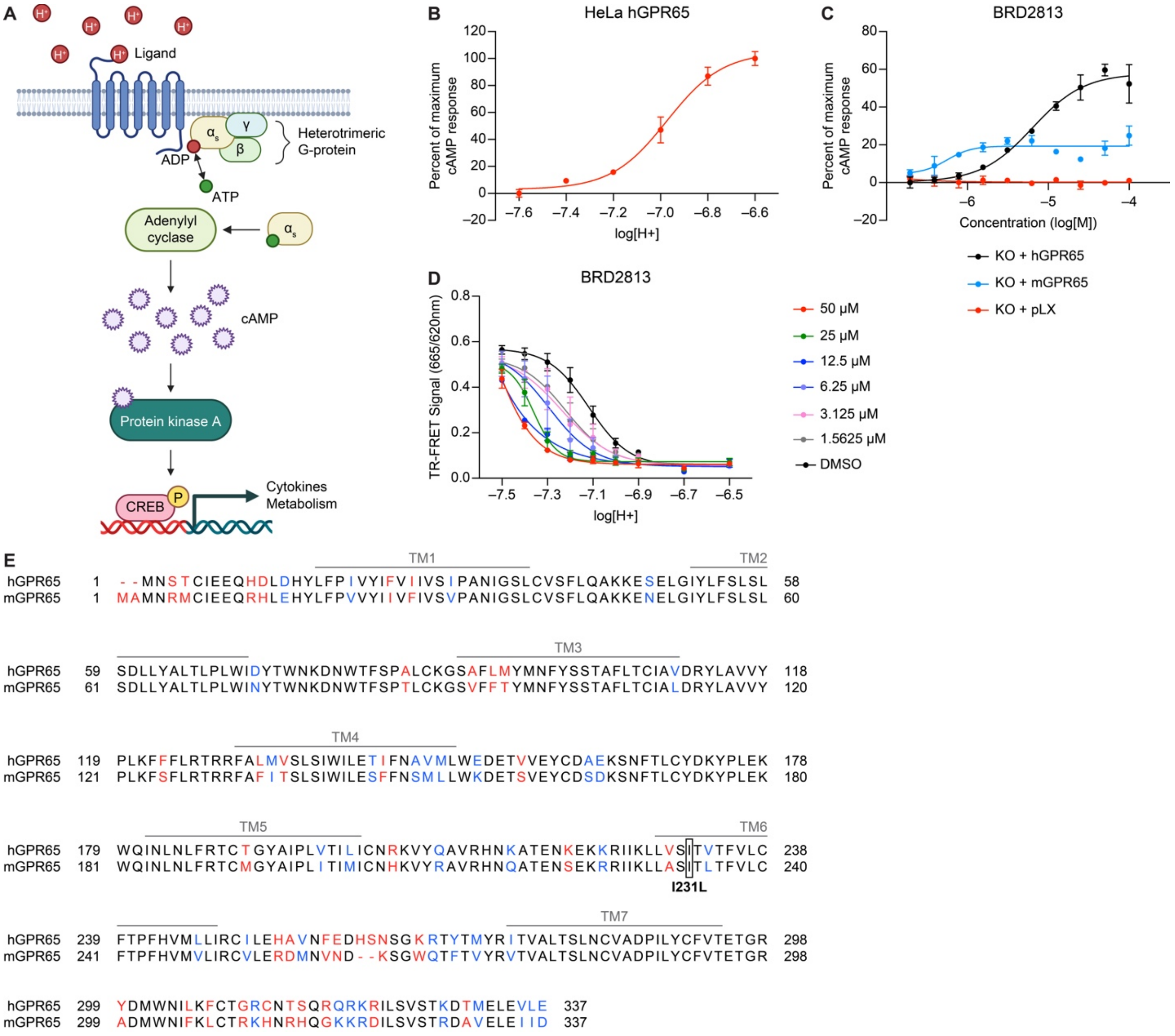

Fig. S1.

Low pH and BRD2813 induce cAMP in hGPR65-expressing cells. (A) Diagram of Gα_s-coupled signal transduction pathway. Created with BioRender.com. **(B)** cAMP response of hGPR65-expressing HeLa cells stimulated at pH 6.6-7.6 for 30 min. Responses were normalized to highest and lowest pH values (mean ± SD, n=8). **(C)** BRD2813 specificity for GPR65 in GPR65 KO HeLa cells reconstituted with hGPR65, mGPR65, or empty pLX vector at pH 7.2 (mean ± SD, n=2). **(D)** pH-dependent cAMP production in the presence of 0-50 μM BRD2813, showing positive allosteric modulation behavior. pH curves are plotted with raw TR-FRET signal (mean ± SD, n=2). **(E)** Sequence alignment of hGPR65 and mGPR65. The transmembrane domains (TM1-7) are annotated in gray. The location of the human I231L variant is marked with a box. Identical residues are in black, similar residues are in blue, and red indicates residues that are not similar. Computed sequence identity: 77.88%. Alignment was generated and analyzed using SnapGene.

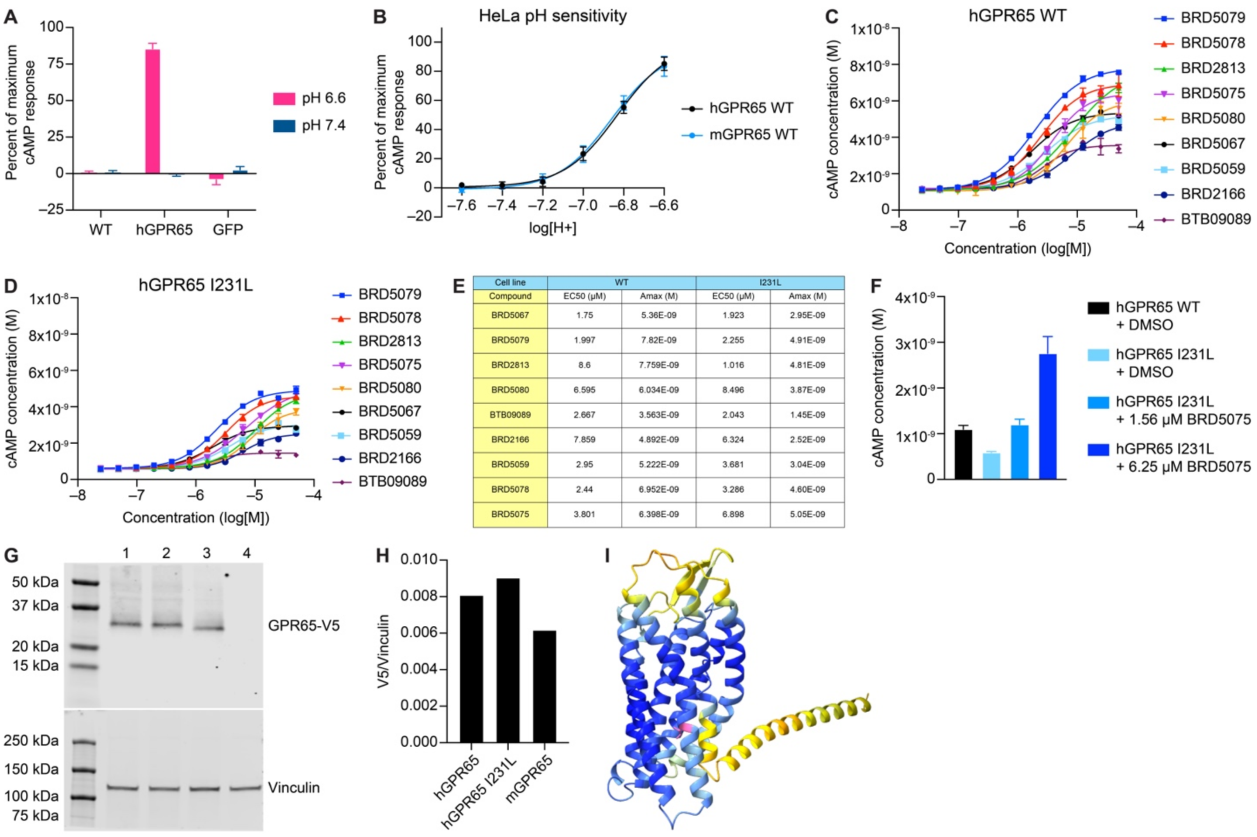

Cell line	WT		I231L	
Compound	EC50 (µM)	Amax (M)	EC50 (µM)	Amax (M)
BRD5067	1.75	5.36E-09	1.923	2.95E-09
BRD5079	1.997	7.82E-09	2.255	4.91E-09
BRD2813	8.6	7.759E-09	1.016	4.81E-09
BRD5080	6.595	6.034E-09	8.496	3.87E-09
BTB09089	2.667	3.563E-09	2.043	1.45E-09
BRD2166	7.859	4.892E-09	6.324	2.52E-09
BRD5059	2.95	5.222E-09	3.681	3.04E-09
BRD5078	2.44	6.952E-09	3.286	4.60E-09
BRD5075	3.801	6.398E-09	6.898	5.05E-09

Fig. S2.

BRD2813 analogs induce cAMP in GPR65 I231L-expressing cells. (A) pH-dependent cAMP response in WT HeLa cells and WT HeLa cells expressing hGPR65 or GFP. Cells were stimulated at pH 6.6 or 7.4 for 30 min. Responses were normalized to DMSO and 100 µM forskolin treatments at pH 7.4 (mean ± SD, n=8). **(B)** cAMP response of hGPR65-expressing and mGPR65-expressing HeLa cells stimulated at pH 6.6-7.6 for 30 min. Responses were normalized to highest and lowest pH values (mean ± SD, n=8). **(C,D)** cAMP response of (C) hGPR65-expressing and (D) hGPR65 I231L-expressing HeLa cells stimulated at pH 7.2 with prioritized analogs and BTB09089 in 12-point dose. Data were calculated as absolute cAMP values from a standard curve (mean ± SD, n=3). **(E)** Summary table of EC_{50} and A_{max} from (C,D) **(F)** cAMP response of hGPR65-expressing and hGPR65 I231L-expressing HeLa cells at pH 7.2 (DMSO) and cAMP induction in hGPR65 I231L-expressing cells upon addition of 1.56 µM and 6.25 µM BRD5075 (mean ± SD, n=3). **(G)** Western blot showing GPR65 expression of (1) hGPR65-expressing, (2) hGPR65 I231L-expressing, (3) mGPR65-expressing and (4) GFP-expressing HeLa cell lines. **(H)** Quantification of (G). **(I)** AlphaFold 2 prediction of hGPR65 structure colored with AlphaFold confidence color scale (dark blue: very high, light blue: confident, yellow: low, orange: very low). The location of I231 is highlighted in pink. Image was generated with ChimeraX (*58*).

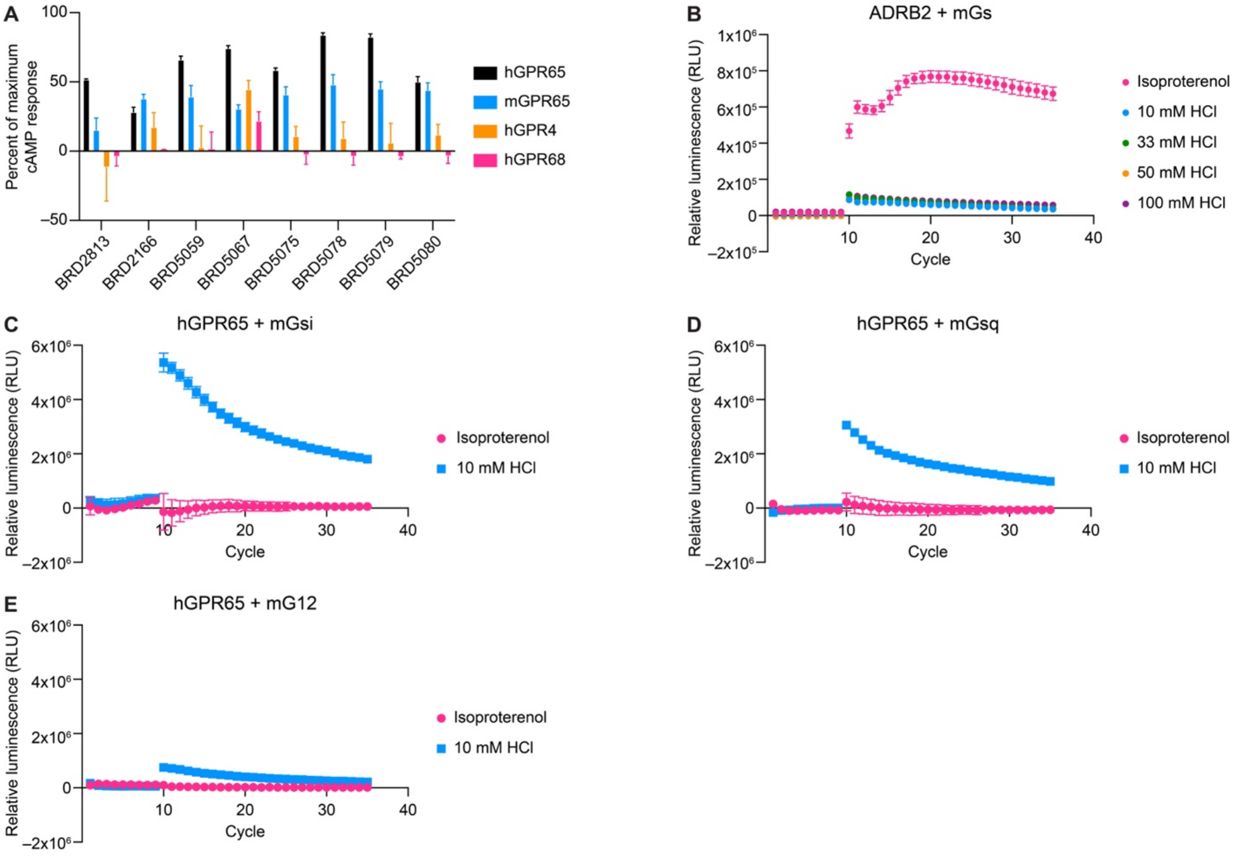

Fig. S3.

Specificity of prioritized BRD2813 analogs and miniG recruitment to ADRB2 and hGPR65. (A) cAMP response to 1.56 µM of prioritized compounds in hGPR65-expressing, mGPR65-expressing, hGPR4-expressing, and hGPR68-expressing HeLa cells at pH 7.2. Responses were normalized to DMSO for minimum and pH 6.6 for maximum response (mean ± SD, n=3). **(B)** MiniG recruitment in Expi283T cells co-expressing ADRB2-SmBiT and LgBiT-mGs in response to 10 µM isoproterenol or changes in pH at cycle 10 (mean ± SD, n=3). Data were normalized to cells treated with DMSO at cycle 10. **(C,D,E)** pH-dependent target engagement in Expi283T cells co-expressing hGPR65-SmBiT and (C) LgBiT-mGsi, (D) LgBiT-mGsq, and (E) LgBiT-mG12 (mean ± SD, n=3). 10 µM isoproterenol or 10mM HCl was added at cycle 10. Data were normalized to cells treated with DMSO at cycle 10. 1 cycle = 90s.

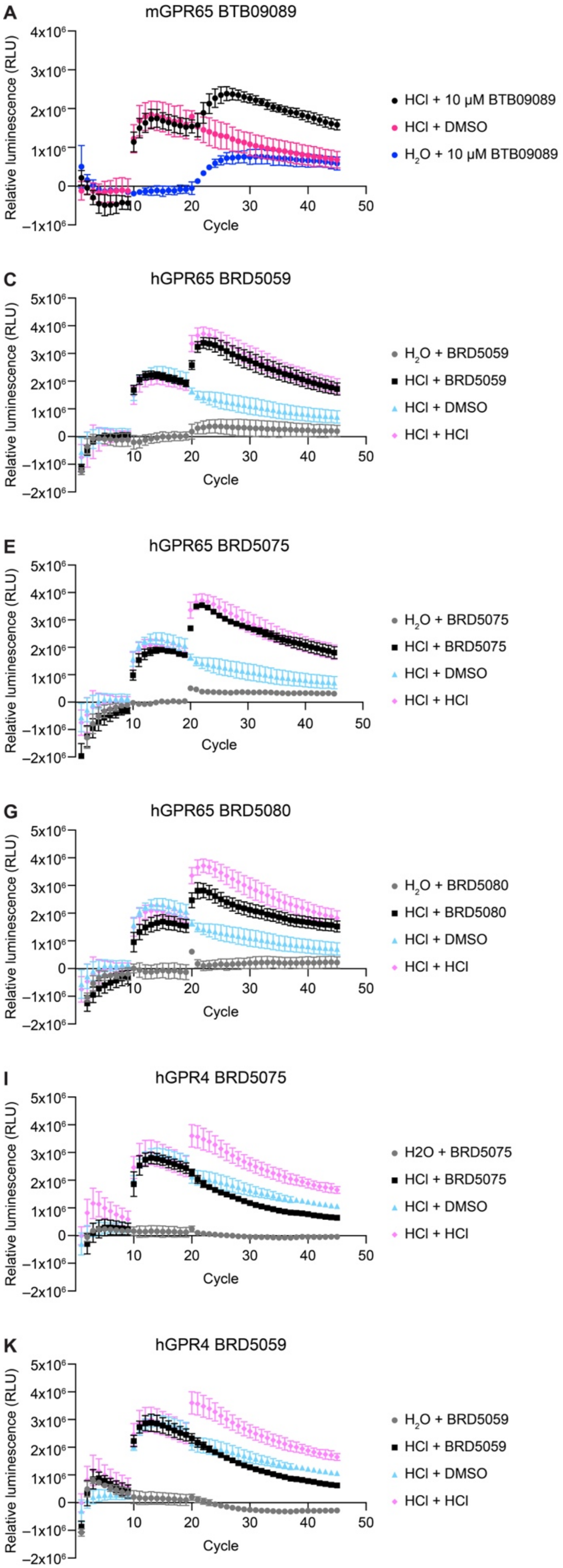

A
mGPR65 BTB09089
Relative luminescence (RLU)
Cycle
HCl + 10 µM BTB09089
HCl + DMSO
H2O + 10 µM BTB09089
B
hGPR65 BTB09089
Relative luminescence (RLU)
Cycle
HCl + 10 µM BTB09089
HCl + DMSO
H2O + 10 µM BTB09089
C
hGPR65 BRD5059
Relative luminescence (RLU)
Cycle
H2O + BRD5059
HCl + BRD5059
HCl + DMSO
HCl + HCl
D
mGPR65 BRD5059
Relative luminescence (RLU)
Cycle
H2O + BRD5059
HCl + BRD5059
HCl + DMSO
HCl + HCl
E
hGPR65 BRD5075
Relative luminescence (RLU)
Cycle
H2O + BRD5075
HCl + BRD5075
HCl + DMSO
HCl + HCl
F
mGPR65 BRD5075
Relative luminescence (RLU)
Cycle
H2O + BRD5075
HCl + BRD5075
HCl + DMSO
HCl + HCl
G
hGPR65 BRD5080
Relative luminescence (RLU)
Cycle
H2O + BRD5080
HCl + BRD5080
HCl + DMSO
HCl + HCl
H
mGPR65 BRD5080
Relative luminescence (RLU)
Cycle
H2O + BRD5080
HCl + BRD5080
HCl + DMSO
HCl + HCl
I
hGPR4 BRD5075
Relative luminescence (RLU)
Cycle
H2O + BRD5075
HCl + BRD5075
HCl + DMSO
HCl + HCl
J
hGPR4 BRD5080
Relative luminescence (RLU)
Cycle
H2O + BRD5080
HCl + BRD5080
HCl + DMSO
HCl + HCl
K
hGPR4 BRD5059
Relative luminescence (RLU)
Cycle
H2O + BRD5059
HCl + BRD5059
HCl + DMSO
HCl + HCl

Fig. S4.

BRD2813 analogs display positive allosteric modulation of hGPR65 and mGPR65 but not hGPR4. (A,B) LgBiT-mGs recruitment in Expi293T cells expressing (A) mGPR65-SmBiT and (B) hGPR65-SmBiT. H_2O or HCl was added at cycle 10, followed by DMSO or 10 μM BTB09089 at cycle 20 (mean ± SD, n=3). Data were normalized to cells treated with H_2O at cycle 10 and DMSO at cycle 20. **(C-H)** LgBiT-mGs recruitment in Expi293T cells expressing (C,E,G) hGPR65-SmBiT and (D,F,H) mGPR65-SmBiT. H_2O or HCl was added at cycle 10, followed by 10 μM BRD5075, 10 μM BRD5080, DMSO, or HCl at cycle 20 (mean ± SD, n=3). Data were normalized to cells treated with H_2O at cycle 10 and DMSO at cycle 20. **(I,J,K)** LgBiT-mGs recruitment in Expi293T cells expressing hGPR4-SmBiT. H_2O or HCl was added at cycle 10, followed by DMSO, HCl, or (I) 10 μM BRD5075, (J) 10 μM BRD5080, and (K) 10 μM BRD5059 at cycle 20 (mean ± SD, n=3). Data were normalized to cells treated with H_2O at cycle 10 and DMSO at cycle 20. 1 cycle = 90s.

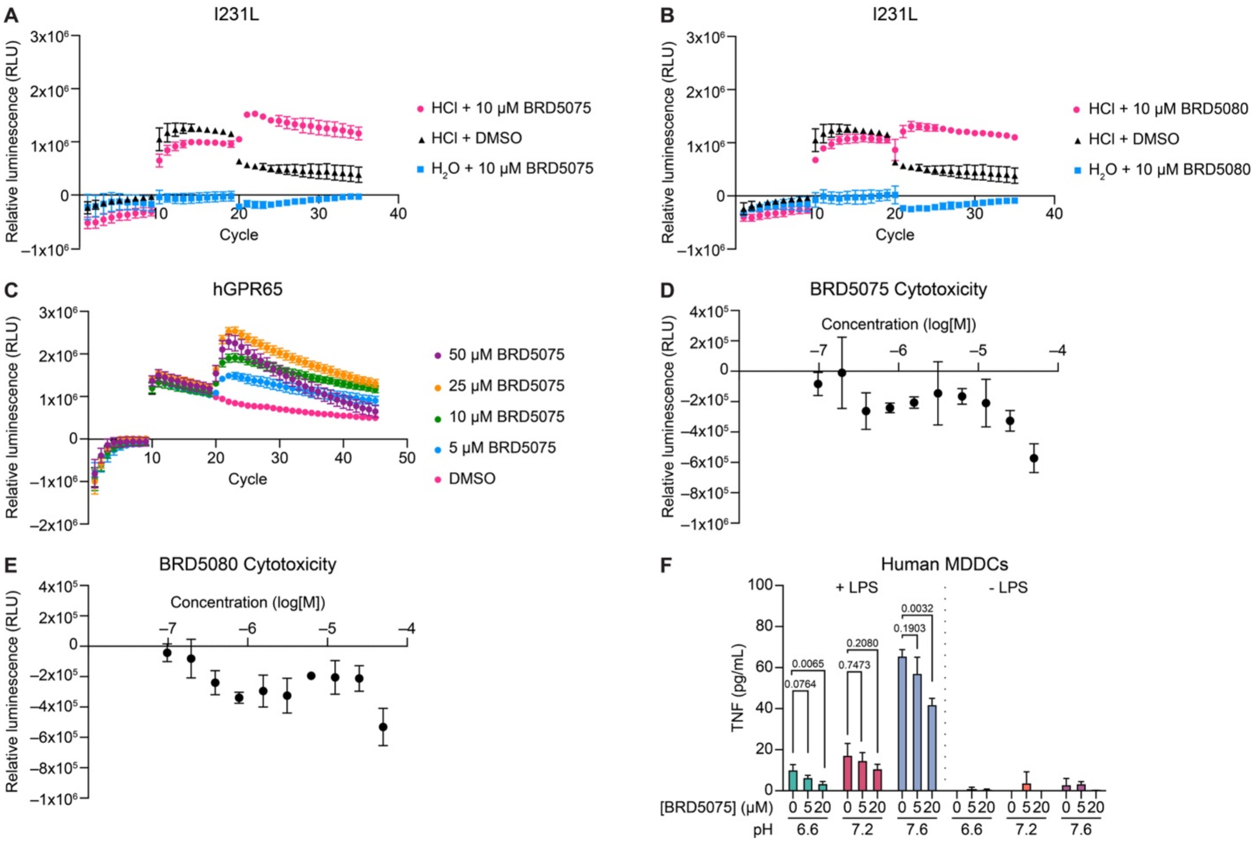

Fig. S5.

BRD5075 recruits miniG protein to GPR65 I231L and decreases TNF production in MDDCs. (A,B) LgBiT-mGs recruitment in Expi293T cells expressing hGPR65 I231L-SmBiT. H_2O or HCl was added at cycle 10, followed by 10 µM (A) BRD5075 or (B) BRD5080 at cycle 20. Data were normalized to cells treated with H_2O at cycle 10 and DMSO at cycle 20 (mean ± SD, n=3). **(C)** LgBiT-mGs recruitment in Expi293T cells expressing hGPR65-SmBiT. H_2O or HCl was added at cycle 10, followed by 0-50 µM BRD5075 at cycle 20. Data were normalized to cells treated with H_2O at cycle 10 and DMSO at cycle 20 (mean ± SD, n=3).1 cycle = 90s. **(D,E)** CellTiter-Glo assay after 72 hr treatment of hGPR65-expressing HeLa cells with 0-50 µM (A) BRD5075 and (B) BRD5080 (mean ± SD, n=3). Relative luminescence was calculated relative to DMSO. **(F)** TNF secretion by human MDDCs co-stimulated with 100 ng/mL LPS and 0-20 µM BRD5075 for 3 h at varying pH (mean ± SD, n=3 technical replicates from 1 donor). P values indicated above bars were determined using a one-way ANOVA.

Data S1. (separate file)

Specificity of primary hits from 21K screen. Cyclic AMP production after 30 min compound stimulation in GPR65 KO HeLa cells reconstituted with hGPR65 or empty vector at pH 7.1. Responses were normalized to DMSO and 100 μM forskolin treatments (mean ± SD, n=2).

Data S2. (separate file)

SAR table of all BRD2813 analogs. Cyclic AMP production after 30 min compound stimulation in HeLa cells expressing hGPR65, mGPR65, or GFP at pH 7.2. Responses were normalized to DMSO and 100 μM forskolin treatments (mean ± SD, n=2). NA = no activity, N/A = not applicable.

Data S3. (separate file)

Specificity and physical properties of prioritized BRD2813 analogs. Cyclic AMP production after 30 min compound stimulation in HeLa cells expressing hGPR65, mGPR65, hGPR4, or hGPR68. Responses were normalized to DMSO and pH 6.6 treatments (mean ± SD, n=2). NA = no activity, N/A = not applicable.

Data S4. (separate file)

GPR65-dependent DEGs. Significant DEGs unique to WT BMDCs or shared between WT and I231L BMDCs treated with BRD5075 compared to DMSO at pH 7.2. $p_{adj} < 0.1$.

REFERENCES AND NOTES

1. M. R. Nelson, H. Tipney, J. L. Painter, J. Shen, P. Nicoletti, Y. Shen, A. Floratos, P. C. Sham, M. J. Li, J. Wang, L. R. Cardon, J. C. Whittaker, P. Sanseau, The support of human genetic evidence for approved drug indications. Nat. Genet.**47**, 856–860 (2015).

2. M. C. Sadler, C. Auwerx, P. Deelen, Z. Kutalik, Multi-layered genetic approaches to identify approved drug targets. *Cell Genom.* **3**, 100341 (2023).

3. L. Kong, V. Pokatayev, A. Lefkovith, G. T. Carter, E. A. Creasey, C. Krishna, S. Subramanian, B. Kochar, O. Ashenberg, H. Lau, A. N. Ananthakrishnan, D. B. Graham, J. Deguine, R. J. Xavier, The landscape of immune dysregulation in Crohn's disease revealed through single-cell transcriptomic profiling in the ileum and colon. *Immunity* **56**, 444–458.e5 (2023).

4. C. S. Smillie, M. Biton, J. Ordovas-Montanes, K. M. Sullivan, G. Burgin, D. B. Graham, R. H. Herbst, N. Rogel, M. Slyper, J. Waldman, M. Sud, E. Andrews, G. Velonias, A. L. Haber, K. Jagadeesh, S. Vickovic, J. Yao, C. Stevens, D. Dionne, L. T. Nguyen, A.-C. Villani, M. Hofree, E. A. Creasey, H. Huang, O. Rozenblatt-Rosen, J. J. Garber, H. Khalili, A. N. Desch, M. J. Daly, A. N. Ananthakrishnan, A. K. Shalek, R. J. Xavier, A. Regev, Intra- and inter-cellular rewiring of the human colon during ulcerative colitis. *Cell***178**, 714–730.e22 (2019).

5. J. B. Rowe, N. J. Kapolka, G. J. Taghon, W. M. Morgan, D. G. Isom, The evolution and mechanism of GPCR proton sensing. J. Biol. Chem.**296**, 100167 (2021).

6. J.-Q. Wang, J. Kon, C. Mogi, M. Tobo, A. Damirin, K. Sato, M. Komachi, E. Malchinkhuu, N. Murata, T. Kimura, A. Kuwabara, K. Wakamatsu, H. Koizumi, T. Uede, G. Tsujimoto, H. Kurose, T. Sato, A. Harada, N. Misawa, H. Tomura, F. Okajima, TDAG8 is a proton-sensing and psychosine-sensitive G-protein-coupled receptor. J. Biol. Chem.**279**, 45626–45633 (2004).

7. M.-G. Ludwig, M. Vanek, D. Guerini, J. A. Gasser, C. E. Jones, U. Junker, H. Hofstetter, R. M. Wolf, K. Seuwen, Proton-sensing G-protein-coupled receptors. *Nature***425**, 93–98 (2003).

8. Y. Timasheva, T. R. Nasibullin, I. A. Tuktarova, V. V. Erdman, T. R. Galiullin, O. V. Zaplakhova, K. Z. Bakhtiiarova, Multilocus evaluation of genetic predictors of multiple sclerosis. *Gene* **809**, 146008 (2022).

9. K. M. de Lange, L. Moutsianas, J. C. Lee, C. A. Lamb, Y. Luo, N. A. Kennedy, L. Jostins, D. L. Rice, J. Gutierrez-Achury, S.-G. Ji, G. Heap, E. R. Nimmo, C. Edwards, P. Henderson, C. Mowat, J. Sanderson, J. Satsangi, A. Simmons, D. C. Wilson, M. Tremelling, A. Hart, C. G. Mathew, W. G. Newman, M. Parkes, C. W. Lees, H. Uhlig, C. Hawkey, N. J. Prescott, T. Ahmad, J. C. Mansfield, C. A. Anderson, J. C. Barrett, Genome-wide association study implicates immune activation of multiple integrin genes in inflammatory bowel disease. Nat. Genet. **49**, 256–261 (2017).

10. L. Jostins, S. Ripke, R. K. Weersma, R. H. Duerr, D. P. McGovern, K. Y. Hui, J. C. Lee, L. P. Schumm, Y. Sharma, C. A. Anderson, J. Essers, M. Mitrovic, K. Ning, I. Cleynen, E. Theatre, S. L. Spain, S. Raychaudhuri, P. Goyette, Z. Wei, C. Abraham, J.-P. Achkar, T. Ahmad, L. Amininejad, A. N. Ananthakrishnan, V. Andersen, J. M. Andrews, L. Baidoo, T. Balschun, P. A. Bampton, A. Bitton, G. Boucher, S. Brand, C. Büning, A. Cohain, S. Cichon, M. D'Amato, D. De Jong, K. L. Devaney, M. Dubinsky, C. Edwards, D. Ellinghaus, L. R. Ferguson, D. Franchimont, K. Fransen, R. Gearry, M. Georges, C. Gieger, J. Glas, T. Haritunians, A. Hart, C. Hawkey, M. Hedl, X. Hu, T. H. Karlsen, L. Kupcinskas, S. Kugathasan, A. Latiano, D. Laukens, I. C. Lawrance, C. W. Lees, E. Louis, G. Mahy, J. Mansfield, A. R. Morgan, C. Mowat, W. Newman, O. Palmieri, C. Y. Ponsioen, U. Potocnik, N. J. Prescott, M. Regueiro, J. I. Rotter, R. K. Russell, J. D. Sanderson, M. Sans, J. Satsangi, S. Schreiber, L. A. Simms, J. Sventoraityte, S. R. Targan, K. D. Taylor, M. Tremelling, H. W. Verspaget, M. De Vos, C. Wijmenga, D. C. Wilson, J. Winkelmann, R. J. Xavier, S. Zeissig, B. Zhang, C. K. Zhang, H. Zhao; International IBD Genetics Consortium (IIBDGC), M. S. Silverberg, V. Annese, H. Hakonarson, S. R. Brant, G. Radford-Smith, C. G. Mathew, J. D. Rioux, E. E. Schadt, M. J. Daly, A. Franke, M. Parkes, S. Vermeire, J. C. Barrett, J. H. Cho, Host-microbe interactions have shaped the genetic architecture of inflammatory bowel disease. *Nature* **491**, 119–124 (2012).

11. J. Z. Liu, S. van Sommeren, H. Huang, S. C. Ng, R. Alberts, A. Takahashi, S. Ripke, J. C. Lee, L. Jostins, T. Shah, S. Abedian, J. H. Cheon, J. Cho, N. E. Dayani, L. Franke, Y. Fuyuno, A.

Hart, R. C. Juyal, G. Juyal, W. H. Kim, A. P. Morris, H. Poustchi, W. G. Newman, V. Midha, T. R. Orchard, H. Vahedi, A. Sood, J. Y. Sung, R. Malekzadeh, H.-J. Westra, K. Yamazaki, S.-K. Yang; International Multiple Sclerosis Genetics Consortium; International IBD Genetics Consortium, J. C. Barrett, B. Z. Alizadeh, M. Parkes, T. Bk, M. J. Daly, M. Kubo, C. A. Anderson, R. K. Weersma, Association analyses identify 38 susceptibility loci for inflammatory bowel disease and highlight shared genetic risk across populations. Nat. Genet. **47**, 979–986 (2015).

12. Y. Yuan, Y. Ma, X. Zhang, R. Han, X. Hu, J. Yang, M. Wang, S.-Y. Guan, G. Pan, S.-Q. Xu, S. Jiang, F. Pan, Genetic polymorphisms of G protein-coupled receptor 65 gene are associated with ankylosing spondylitis in a Chinese Han population: A case-control study. Hum. Immunol. **80**, 146–150 (2019).

13. L. Xie, C. I. McKenzie, X. Qu, Y. Mu, Q. Wang, N. Bing, K. Naidoo, M. J. Alam, D. Yu, F. Gong, C. Ang, R. Robert, F. Z. Marques, N. Furlotte, D. Hinds, O. Gasser; 23andMe Research Team, R. J. Xavier, C. R. Mackay, pH and proton sensor GPR65 determine susceptibility to atopic dermatitis. J. Immunol. **207**, 101–109 (2021).

14. H. Tsurumaki, C. Mogi, H. Aoki-Saito, M. Tobo, Y. Kamide, M. Yatomi, K. Sato, K. Dobashi, T. Ishizuka, T. Hisada, M. Yamada, F. Okajima, Protective role of proton-sensing TDAG8 in lipopolysaccharide-induced acute lung injury. Int. J. Mol. Sci. **16**, 28931–28942 (2015).

15. Y. Onozawa, T. Komai, T. Oda, Activation of T cell death-associated gene 8 attenuates inflammation by negatively regulating the function of inflammatory cells. Eur. J. Pharmacol. **654**, 315–319 (2011).

16. R. C. Wirasinha, D. Vijayan, N. J. Smith, G. P. Parnell, A. Swarbrick, R. Brink, C. King, G. Stewart, D. R. Booth, M. Batten, GPR65 inhibits experimental autoimmune encephalomyelitis through CD4+ T cell independent mechanisms that include effects on iNKT cells. Immunol. Cell Biol. **96**, 128–136 (2018).

17. J. T. Gaublomme, N. Yosef, Y. Lee, R. S. Gertner, L. V. Yang, C. Wu, P. P. Pandolfi, T. Mak, R. Satija, A. K. Shalek, V. K. Kuchroo, H. Park, A. Regev, Single-cell genomics unveils critical regulators of Th17 cell pathogenicity. *Cell* **163**, 1400–1412 (2015).

18. K. G. Lassen, C. I. McKenzie, M. Mari, T. Murano, J. Begun, L. A. Baxt, G. Goel, E. J. Villablanca, S.-Y. Kuo, H. Huang, L. Macia, A. K. Bhan, M. Batten, M. J. Daly, F. Reggiori, C. R. Mackay, R. J. Xavier, Genetic coding variant in GPR65 alters lysosomal pH and links lysosomal dysfunction with colitis risk. *Immunity* **44**, 1392–1405 (2016).

19. X. Chen, A. Jaiswal, Z. Costliow, P. Herbst, E. A. Creasey, N. Oshiro-Rapley, M. J. Daly, K. L. Carey, D. B. Graham, R. J. Xavier, pH sensing controls tissue inflammation by modulating cellular metabolism and endo-lysosomal function of immune cells. Nat. Immunol. **23**, 1063–1075 (2022).

20. I. Tcymbarevich, S. M. Richards, G. Russo, J. Kühn-Georgijevic, J. Cosin-Roger, K. Baebler, S. Lang, S. Bengs, K. Atrott, C. Bettoni, S. Gruber, I. Frey-Wagner, M. Scharl, B. Misselwitz, C. A. Wagner, K. Seuwen, G. Rogler, P. A. Ruiz, M. Spalinger, C. de Vallière, Lack of the pH-sensing receptor TDAG8 [GPR65] in macrophages plays a detrimental role in murine models of inflammatory bowel disease. *J. Crohns Colitis***13**, 245–258 (2019).

21. Y. Wang, C. de Vallière, P. H. Imenez Silva, I. Leonardi, S. Gruber, A. Gerstgrasser, H. Melhem, A. Weber, K. Leucht, L. Wolfram, M. Hausmann, C. Krieg, K. Thomasson, O. Boyman, I. Frey-Wagner, G. Rogler, C. A. Wagner, The proton-activated receptor GPR4 modulates intestinal inflammation. *J. Crohns Colitis* **12**, 355–368 (2018)

22. C. de Vallière, K. Bäbler, P. Busenhart, M. Schwarzfischer, C. Maeyashiki, C. Schuler, K. Atrott, S. Lang, M. R. Spalinger, M. Scharl, P. A. Ruiz-Castro, M. Hausmann, G. Rogler, A novel OGR1 (GPR68) inhibitor attenuates inflammation in murine models of colitis. *Inflamm. Intest. Dis.* **6**, 140–153 (2021).

23. H. Huang, M. Fang, L. Jostins, M. U. Mirkov, G. Boucher, C. A. Anderson, V. Andersen, I. Cleynen, A. Cortes, F. Crins, M. D'Amato, V. Deffontaine, J. Dmitrieva, E. Docampo, M. Elansary, K. K.-H. Farh, A. Franke, A.-S. Gori, P. Goyette, J. Halfvarson, T. Haritunians, J.

Knight, I. C. Lawrance, C. W. Lees, E. Louis, R. Mariman, T. Meuwissen, M. Mni, Y. Momozawa, M. Parkes, S. L. Spain, E. Théâtre, G. Trynka, J. Satsangi, S. van Sommeren, S. Vermeire, R. J. Xavier; International Inflammatory Bowel Disease Genetics Consortium, R. K. Weersma, R. H. Duerr, C. G. Mathew, J. D. Rioux, D. P. B. McGovern, J. H. Cho, M. Georges, M. J. Daly, J. C. Barrett, Fine-mapping inflammatory bowel disease loci to single-variant resolution. *Nature* **547**, 173–178 (2017).

24. Z. Liu, R. Liu, H. Gao, S. Jung, X. Gao, R. Sun, X. Liu, Y. Kim, H.-S. Lee, Y. Kawai, M. Nagasaki, J. Umeno, K. Tokunaga, Y. Kinouchi, A. Masamune, W. Shi, C. Shen, Z. Guo, K. Yuan; FinnGen; International Inflammatory Bowel Disease Genetics Consortium; Chinese Inflammatory Bowel Disease Genetics Consortium, S. Zhu, D. Li, J. Liu, T. Ge, J. Cho, M. J. Daly, D. P. B. McGovern, B. D. Ye, K. Song, Y. Kakuta, M. Li, H. Huang, Genetic architecture of the inflammatory bowel diseases across East Asian and European ancestries. Nat. Genet. **55**, 796–806 (2023).

25. V. Mercier, G. Boucher, D. Devost, K. Bourque, A. Alikashani, C. Beauchamp, A. Bitton, S. Foisy, P. Goyette, G. Charron, T. E. Hébert, J. D. Rioux, IBD-associated G protein-coupled receptor 65 variant compromises signalling and impairs key functions involved in inflammation. Cell. Signal. **93**, 110294 (2022).

26. L. M. Morales Rodríguez, S. E. Crilly, J. B. Rowe, D. G. Isom, M. A. Puthenveedu, Location-biased activation of the proton-sensor GPR65 is uncoupled from receptor trafficking. *Proc. Natl. Acad. Sci. U.S.A.***120**, e2302823120 (2023).

27. A. L. Martin, M. A. Steurer, R. S. Aronstam, Constitutive activity among orphan class-A G protein coupled receptors. *PLOS ONE* **10**, e0138463 (2015).

28. R. Lin, W. Wu, H. Chen, H. Gao, X. Wu, G. Li, Q. He, H. Lu, M. Sun, Z. Liu, GPR65 promotes intestinal mucosal Th1 and Th17 cell differentiation and gut inflammation through downregulating NUAK2. Clin. Transl. Med.**12**, e771 (2022).

29. C. Mogi, M. Tobo, H. Tomura, N. Murata, X.-D. He, K. Sato, T. Kimura, T. Ishizuka, T. Sasaki, T. Sato, Y. Kihara, S. Ishii, A. Harada, F. Okajima, Involvement of proton-sensing

TDAG8 in extracellular acidification-induced inhibition of proinflammatory cytokine production in peritoneal macrophages. J. Immunol.**182**, 3243–3251 (2009).

30. H. Wu, V. Estrella, M. Beatty, D. Abrahams, A. El-Kenawi, S. Russell, A. Ibrahim-Hashim, D. L. Longo, Y. K. Reshetnyak, A. Moshnikova, O. A. Andreev, K. Luddy, M. Damaghi, K. Kodumudi, S. R. Pillai, P. Enriquez-Navas, S. Pilon-Thomas, P. Swietach, R. J. Gillies, T-cells produce acidic niches in lymph nodes to suppress their own effector functions. Nat. Commun. **11**, 4113 (2020).

31. L. R. Levin, J. Buck, Physiological roles of acid-base sensors. Annu. Rev. Physiol.**77**, 347–362 (2015).

32. C. W. Lindsley, K. A. Emmitte, C. R. Hopkins, T. M. Bridges, K. J. Gregory, C. M. Niswender, P. J. Conn, Practical strategies and concepts in GPCR allosteric modulator discovery: Recent advances with metabotropic glutamate receptors. Chem. Rev. **116**, 6707–6741 (2016).

33. Y. Onozawa, Y. Fujita, H. Kuwabara, M. Nagasaki, T. Komai, T. Oda, Activation of T cell death-associated gene 8 regulates the cytokine production of T cells and macrophages in vitro. Eur. J. Pharmacol. **683**, 325–331 (2012).

34. Q. Wan, N. Okashah, A. Inoue, R. Nehmé, B. Carpenter, C. G. Tate, N. A. Lambert, Mini G protein probes for active G protein-coupled receptors (GPCRs) in live cells. J. Biol. Chem. **293**, 7466–7473 (2018).

35. I. V. Tcymbarevich, J. J. Eloranta, J.-B. Rossel, N. Obialo, M. Spalinger, J. Cosin-Roger, S. Lang, G. A. Kullak-Ublick, C. A. Wagner, M. Scharl, K. Seuwen, P. A. Ruiz, G. Rogler, C. de Vallière, B. Misselwitz; Swiss IBD Cohort Study Group, The impact of the rs8005161 polymorphism on G protein-coupled receptor GPR65 (TDAG8) pH-associated activation in intestinal inflammation. BMC Gastroenterol. **19**, 2 (2019).

36. C. Ponti, D. Gibellini, F. Boin, E. Melloni, F. A. Manzoli, L. Cocco, G. Zauli, M. Vitale, Role of CREB transcription factor in c-fos activation in natural killer cells. Eur. J. Immunol. **32**, 3358–3365 (2002).

37. D. D. Ginty, A. Bonni, M. E. Greenberg, Nerve growth factor activates a Ras-dependent protein kinase that stimulates c-fos transcription via phosphorylation of CREB. *Cell* **77**, 713–725 (1994).

38. A. L. Hopkins, C. R. Groom, The druggable genome. Nat. Rev. Drug Discov. **1**, 727–730 (2002).

39. B. L. Roth, W. K. Kroeze, Integrated approaches for genome-wide interrogation of the druggable non-olfactory G protein-coupled receptor superfamily. J. Biol. Chem. **290**, 19471–19477 (2015).

40. M. Rask-Andersen, S. Masuram, H. B. Schiöth, The druggable genome: Evaluation of drug targets in clinical trials suggests major shifts in molecular class and indication. Annu. Rev. Pharmacol. Toxicol. **54**, 9–26 (2014).

41. X.-P. Huang, J. Karpiak, W. K. Kroeze, H. Zhu, X. Chen, S. S. Moy, K. A. Saddoris, V. D. Nikolova, M. S. Farrell, S. Wang, T. J. Mangano, D. A. Deshpande, A. Jiang, R. B. Penn, J. Jin, B. H. Koller, T. Kenakin, B. K. Shoichet, B. L. Roth, Allosteric ligands for the pharmacologically dark receptors GPR68 and GPR65. *Nature* **527**, 477–483 (2015).

42. W. K. Kroeze, M. F. Sassano, X.-P. Huang, K. Lansu, J. D. McCorvy, P. M. Giguère, N. Sciaky, B. L. Roth, PRESTO-Tango as an open-source resource for interrogation of the druggable human GPCRome. Nat. Struct. Mol. Biol. **22**, 362–369 (2015).

43. N. J. Kapolka, G. J. Taghon, J. B. Rowe, W. M. Morgan, J. F. Enten, N. A. Lambert, D. G. Isom, DCyFIR: A high-throughput CRISPR platform for multiplexed G protein-coupled receptor profiling and ligand discovery. *Proc. Natl. Acad. Sci. U.S.A.***117**, 13117–13126 (2020).

44. M. A. Marie, E. J. Sanderlin, S. Satturwar, H. Hong, K. Lertpiriyapong, D. Donthi, L. V. Yang, GPR65 (TDAG8) inhibits intestinal inflammation and colitis-associated colorectal cancer development in experimental mouse models. Biochim. Biophys. Acta Mol. Basis Dis. **1868**, 166288 (2022).

45. L. Wang, L. Sun, H. Sun, Y. Xing, S. Zhou, G. An, J. Li, K. Ren, J. Sun, GPR65 as a potential immune checkpoint regulates the immune microenvironment according to pan-cancer analysis. *Heliyon* **9**, e13617 (2023).

46. V. H. Wu, B. S. Yung, F. Faraji, R. Saddawi-Konefka, Z. Wang, A. T. Wenzel, M. J. Song, M. S. Pagadala, L. M. Clubb, J. Chiou, S. Sinha, M. Matic, F. Raimondi, T. S. Hoang, R. Berdeaux, D. A. A. Vignali, R. Iglesias-Bartolome, H. Carter, E. Ruppin, J. P. Mesirov, J. S. Gutkind, The GPCR-Gαs-PKA signaling axis promotes T cell dysfunction and cancer immunotherapy failure. Nat. Immunol. **24**, 1318–1330 (2023).

47. H. P. Cho, D. W. Engers, D. F. Venable, C. M. Niswender, C. W. Lindsley, P. J. Conn, K. A. Emmitte, A. L. Rodriguez, A novel class of succinimide-derived negative allosteric modulators of metabotropic glutamate receptor subtype 1 provides insight into a disconnect in activity between the rat and human receptors. *ACS Chem. Nerosci.* **5**, 597–610 (2014).

48. E. Sigel, B. P. Lüscher, A closer look at the high affinity benzodiazepine binding site on GABAA receptors. Curr. Top. Med. Chem. **11**, 241–246 (2011).

49. A. N. Edinoff, A. S. Odisho, K. Lewis, A. Kaskas, G. Hunt, E. M. Cornett, A. D. Kaye, A. Kaye, J. Morgan, P. S. Barrilleaux, D. Lewis, O. Viswanath, I. Urits, Brexanolone, a GABAA modulator, in the treatment of postpartum depression in adults: A comprehensive review. *Front. Psych.***12**, 699740 (2021).

50. L. D. Leader, M. O'Connell, A. VandenBerg, Brexanolone for postpartum depression: Clinical evidence and practical considerations. *Pharmacotherapy***39**, 1105–1112 (2019).

51. M. F. Neurath, Cytokines in inflammatory bowel disease. Nat. Rev. Immunol. **14**, 329–342 (2014).

52. L. Massimino, L. A. Lamparelli, Y. Houshyar, S. D'Alessio, L. Peyrin-Biroulet, S. Vetrano, S. Danese, F. Ungaro, The inflammatory bowel disease transcriptome and metatranscriptome meta-analysis (IBD TaMMA) framework. *Nat. Comput. Sci.* **1**, 511–515 (2021).

53. H. Nie, P. Lin, Y. Zhang, Y. Wan, J. Li, C. Yin, L. Zhang, Single-cell meta-analysis of inflammatory bowel disease with scIBD. *Nat. Comput. Sci.* **3**, 522–531 (2023).

54. T. K. Pedersen, E. M. Brown, D. R. Plichta, J. Johansen, S. W. Twardus, T. M. Delorey, H. Lau, H. Vlamakis, J. J. Moon, R. J. Xavier, D. B. Graham, The CD4+ T cell response to a commensal-derived epitope transitions from a tolerant to an inflammatory state in Crohn's disease. *Immunity* **55**, 1909–1923.e6 (2022).

55. A. Dobin, C. A. Davis, F. Schlesinger, J. Drenkow, C. Zaleski, S. Jha, P. Batut, M. Chaisson, T. R. Gingeras, STAR: Ultrafast universal RNA-seq aligner. *Bioinformatics* **29**, 15–21 (2013).

56. B. Li, C. N. Dewey, RSEM: Accurate transcript quantification from RNA-Seq data with or without a reference genome. *BMC Bioinformatics* **12**, 323 (2011).

57. M. I. Love, W. Huber, S. Anders, Moderated estimation of fold change and dispersion for RNA-seq data with DESeq2. Genome Biol. **15**, 550 (2014).

58. E. C. Meng, T. D. Goddard, E. F. Pettersen, G. S. Couch, Z. J. Pearson, J. H. Morris, T. E. Ferrin, UCSF ChimeraX: Tools for structure building and analysis. Protein Sci. **32**, e4792 (2023).
